# Supplementary material for: Mobile origin-licensing factors confer resistance to conflicts with RNA polymerase
Source: Cell Rep. 2022 Mar 22;38(12):110531. doi: 10.1016/j.celrep.2022.110531 (PMC8961423; doi:10.1016/j.celrep.2022.110531)
Supplement: Document S1. Figures S1–S7 and Table S1 [file mmc1.pdf]

**Cell Reports, Volume *38***

## Supplemental information

# Mobile origin-licensing factors confer resistance to conflicts with RNA polymerase

**Matthias J. Scherr, Syafiq Abd Wahab, Dirk Remus, and Karl E. Duderstadt**

## Supplemental Information

**Table S1. Sequences of oligonucleotides used in this study, Related to STAR methods.**

| Name | Sequence (5´–3´) |
|---|---|
| MS_144 | GGT TTT CCA TGG GTT CCA GCC ATC ACC ACC ACC ATC ATG GCA GTT CGA TGG ACA AAG ATT GCG AAA TGA AAC G |
| MS_145 | GGT TTT CTC GAG TCC CAG ACC CGG TTT ACC CAG AC |
| MS_180 | [PHO]CTA GTC CCG CGA AAT TAA TAC GAC TCA CTA TAG GGA GAC CAC AAC GGT TTG CGA T |
| MS_181 | [PHO]CGC AAA CCG TTG TGG TCT CCC TAT AGT GAG TCG TAT TAA TTT CGC GGG A |
| MS_200 | [PHO]GGC CGG TGA GTG TGT TTG AGT TGA TTT TGT GTG G |
| MS_201 | [BITEG]CCA CAC AAA ATC AAC TCA AAC ACA CTC ACC |
| MS_202 | [PHO]CTA GGG TGA GTG TGT TTG AGT TGA TTT TGT GTG GGC GTC GGT AAG TGA GAG G |
| MS_203 | CCA CAC AAA ATC AAC TCA AAC ACA CTC ACC |
| MS_204 | [BITEG]CCT CTC ACT TAC CGA CGC |
| MS_221 | [PHO]CAT GGA TAG CCT GGA GTT CAT CGC CTC GAA GTT AGC CGG CAG TTC CCA CCA TCA CCA CCA TCA TGG TGG TGG TGG CTC TC |
| MS_222 | [PHO]TCG AGA GAG CCA CCA CCA CCA TGA TGG TGG TGA TGG TGG GAA CTG CCG GCT AAC TTC GAG GCG ATG AAC TCC AGG CTA TC |
| MS_226 | [BITEG]GCT GCT GCG TGT GGA TGA G |
| MS_227 | CCG CGT GCC TGA GTG TTC |

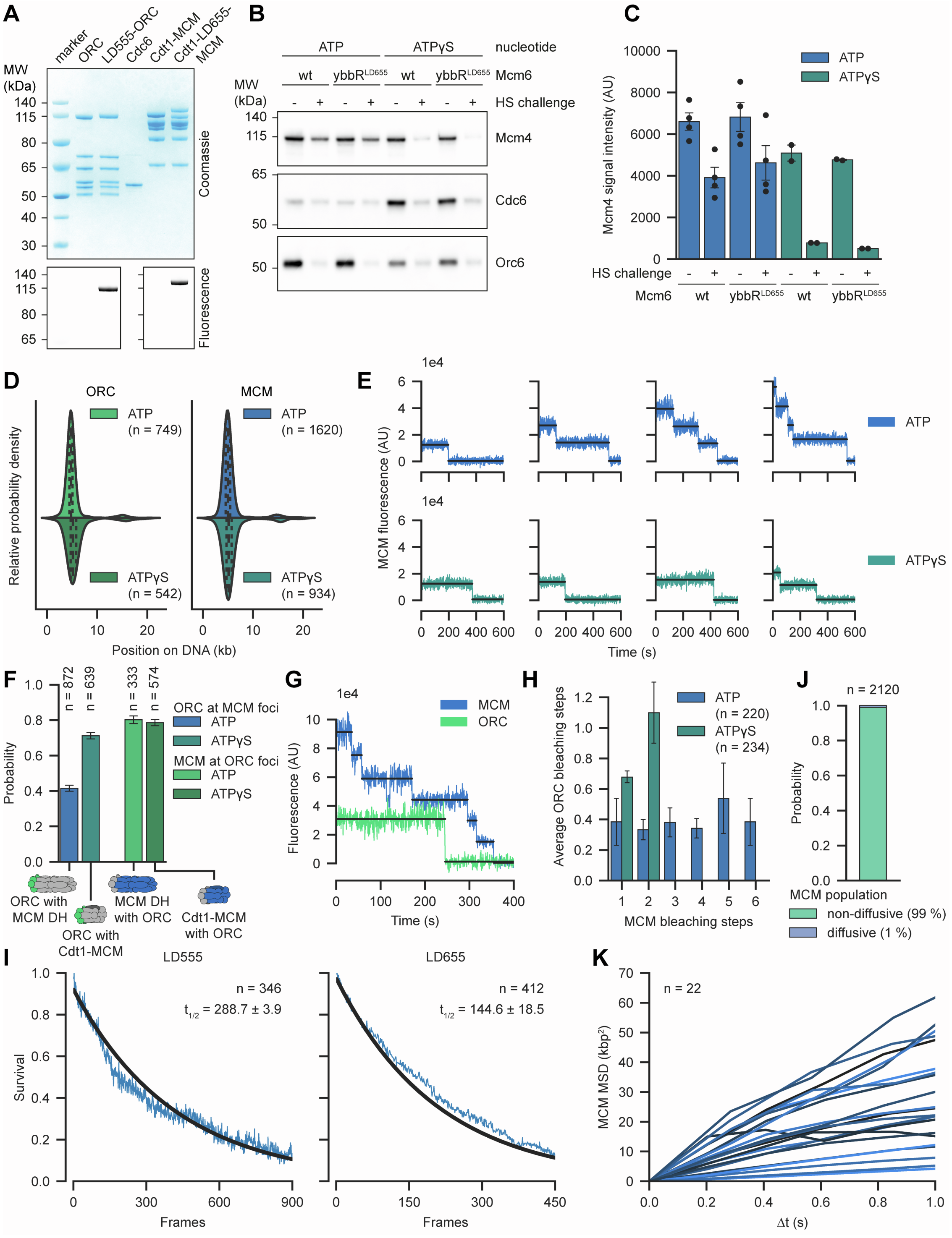


**Figure S1. Additional characterization of MCM loading and diffusion, Related to Figure 1**

(A) SDS-PAGE analysis of purified licensing factors ORC, LD555-ORC, Cdc6, Cdt1-MCM and Cdt1-LD655-MCM by Coomassie Blue staining and fluorescence detection.

(B and C) Ensemble helicase loading assay on bead-bound linear ARS1-DNA with low- or high salt (HS) challenge after licensing in the presence of ATP or ATPγS. (B) Eluted protein was analyzed by Western Blot using protein specific primary antibodies for Orc6, Cdc6 and Mcm4. (C) Integration of Mcm4 signals showed no difference in loading efficiency for Cdt1-MCM containing wildtype or ybbR-LD655 Mcm6 ($n = 4$ and $n = 2$ for ATP and ATPγS condition, respectively).

(D) ORC and MCM binding distribution by KDE on ARS1-DNA after helicase loading in the presence of ATP or ATPγS. Lines represent the quartiles of the distribution with the middle line corresponding to the median. Data depicted is the same as shown in Figure 1B.

(E) Example MCM bleaching trajectories after helicase loading in the presence of ATP or ATPγS.

(F) Fraction of MCM foci colocalizing with ORC and ORC foci colocalizing with MCM after helicase loading in the presence of ATP or ATPγS.

(G) Example bleaching trajectory of ATP-loaded, colocalizing MCM and ORC showing six- and one-step bleaching, respectively.
(H) Average number of ORC molecules colocalizing with MCM foci containing one to six (ATP) or one and two (ATPγS) MCM after helicase loading in the presence of ATP or ATPγS.
(I) Lifetimes of single, surface bound LD555 (left) and LD655 (right) dyes. Data from two independent experiments are shown. These were used to determine the number of frames after which half of the dyes bleached which is displayed as mean ± SD as derived from the fit (black lines).
(J) Observed populations of MCM DHs in the presence of 0.5 M NaCl showed 99 % and 1 % in non-diffusive and diffusive DNA binding mode, respectively.
(K) Plot showing the mean squared displacement (MSD) vs Δt of 22 diffusing MCM DHs in the presence of 0.5 M NaCl. Bar plots in (C), (F), and (H) display the mean and SEM.

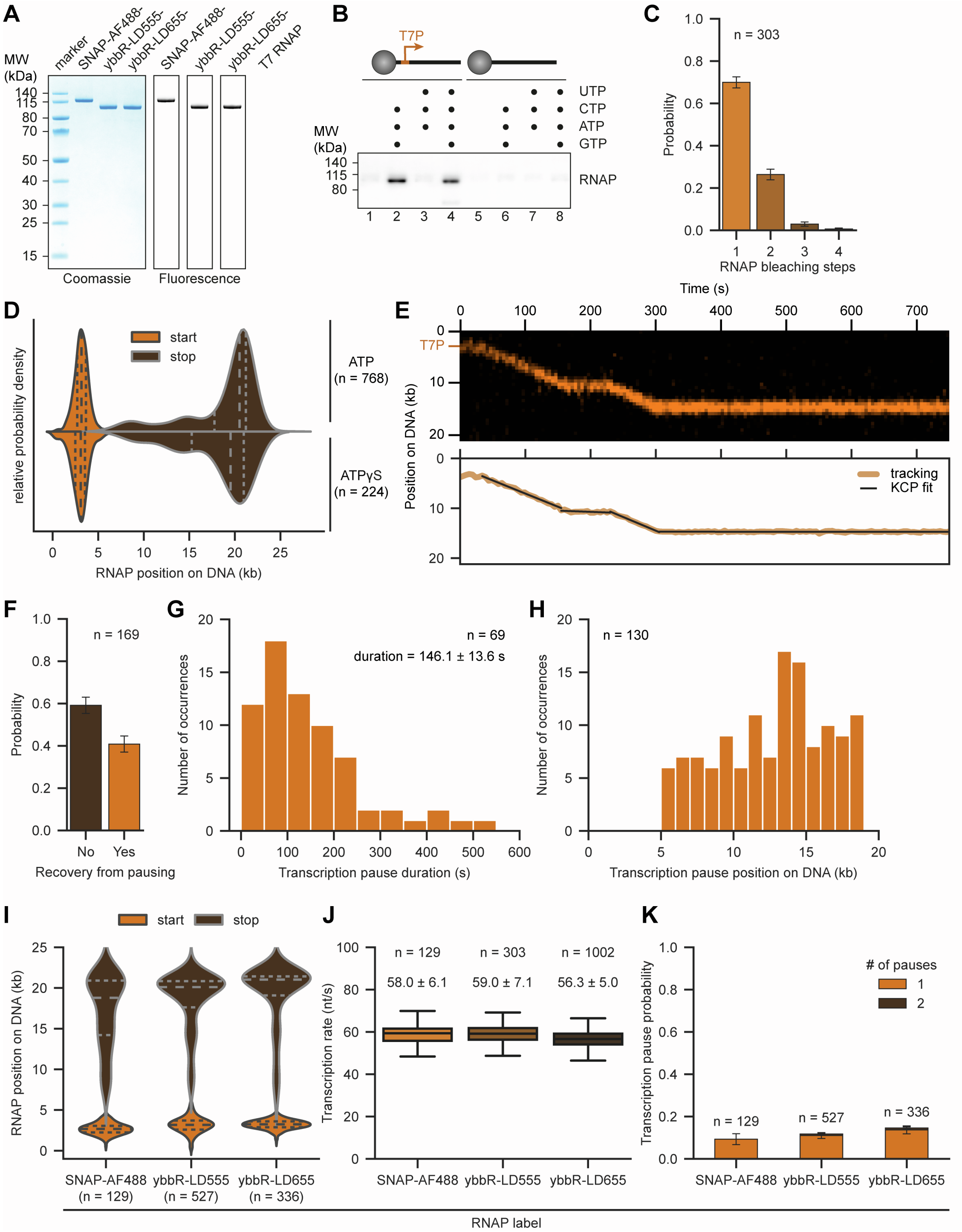


**Figure S2. Additional characterization of the time-coordinated single-molecule transcription assay, Related to Figure 2**

(A) SDS-PAGE analysis of purified SNAP-AF488-, ybbR-LD555- and ybbR-LD655-T7 RNAP by Coomassie Blue staining and fluorescence detection.

(B) Ensemble RNAP loading assay on bead-bound linear DNA with (lanes 1-4) or without (lanes 5-8) an integrated T7P. Formation of stable RNAP elongation complexes were only observed in the presence of an integrated T7P and GTP, ATP and CTP (lanes 2 and 4) but not in the absence of T7P or GTP (lanes 1, 3, 5-8). Eluted RNAP was analyzed by Western Blot using a primary antibody against His tag present in RNAP.

(C) Distribution of RNAP bleaching steps for those loaded at T7P as stalled elongation complexes.

(D) RNAP transcription start and stop sites distribution by KDE on T7P-DNA in the presence of ATP or ATPγS. Lines represent the quartiles of the distribution with the middle line corresponding to the median. Data depicted are the same as shown in Figure 2C.

(E) Representative kymograph (top) with corresponding tracking result (bottom) showing transcribing RNAP with one recovered and one permanent pause detected by a fit with the kinetic change-point (KCP) algorithm (black line).

(F) Fraction of permanent and recovered transcription pauses determined by recovery from pausing within the imaging time.
(G) Distribution of transcription pause durations determined from recovered pauses. Values indicate the mean pause duration ± SEM.
(H) Distribution of transcription pause positions on DNA of permanent and recovered pauses.
(I–K) Transcription start and stop sites (I), rates (J) and pause probability (K) do not differ between SNAP-AF488-, ybbR-LD655- and ybbR-LD655-T7 RNAP. (I) Lines in the violin plot represent the quartiles of the distribution with the middle line corresponding to the median. (J) Values above the box plots indicate the mean ± SD in the presence of ATP derived from a Gaussian fit. Data depicted in (I), (J), and (K) are the same as shown in Figure 2C, 2D, and 2E, respectively. Bar plots in (C), (F), and (K) display the mean and SEM.

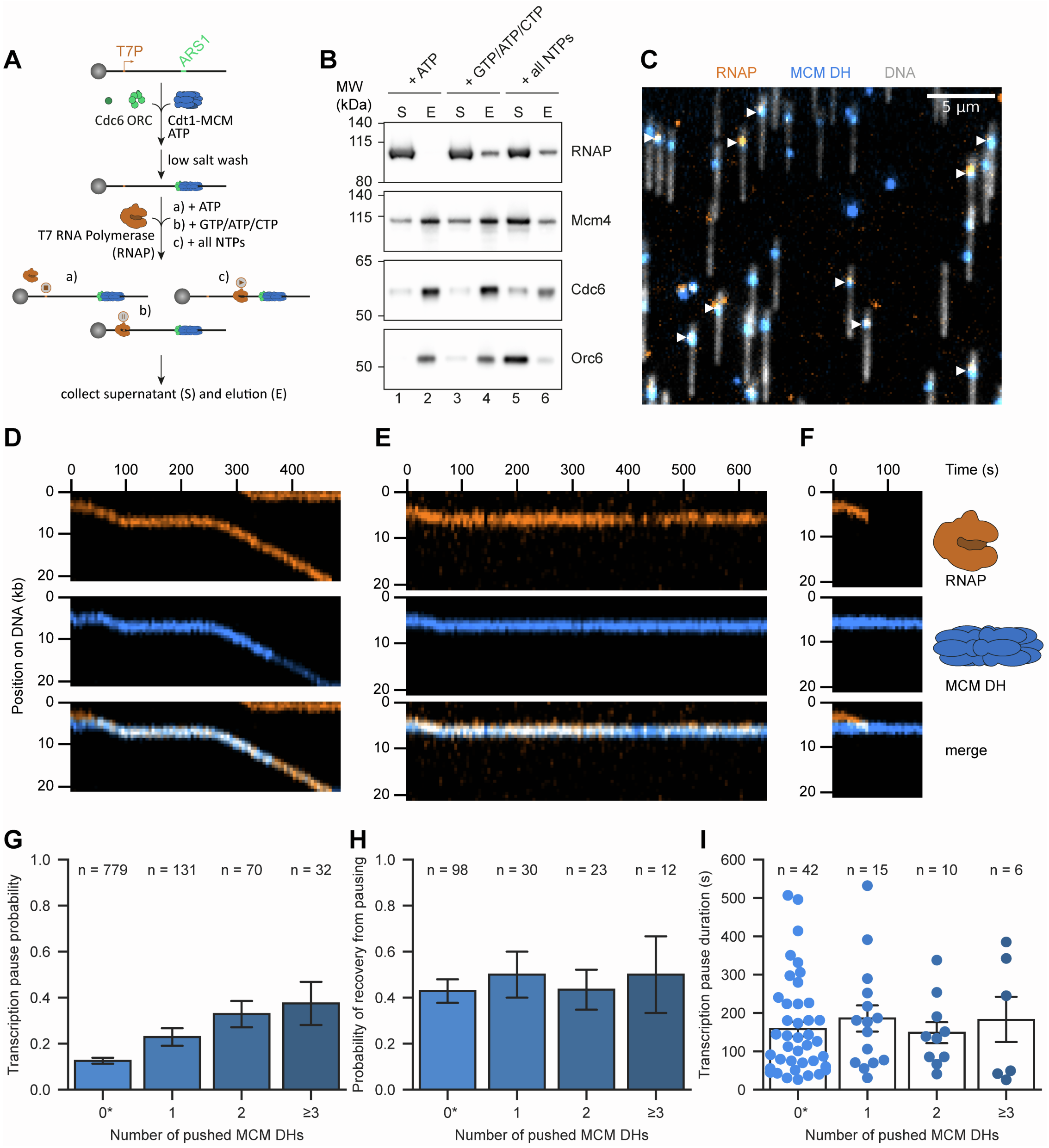


**Figure S3. Additional characterization of transcription-driven MCM DH repositioning, Related to Figure 3**

(A) Schematic of the ensemble RNAP-MCM DH collision assay. MCM DHs were loaded on bead-bound T7P-ARS1-DNA and unbound protein was removed. The reaction was split and incubated with RNAP in the presence of ATP, GTP/ATP/CTP or all NTPs. Finally, the supernatant (S) and elution (E) were collected, corresponding to displaced and DNA-bound protein, respectively.

(B) Western Blot analysis of an ensemble RNAP-MCM DH collision assay as described in (A). In the presence of ATP alone, RNAP could not engage with T7P (lanes 1+2) while in the presence of GTP/ATP/CTP, RNAP formed a stalled elongation complex (lanes 3+4). Both conditions did not show extensive MCM, Cdc6 or ORC displacement from DNA. In the presence of all NTPs, allowing for active transcription, MCM and ORC were efficiently displaced from linear DNA (lanes 5+6).

(C) Example of a partial field of view, showing simultaneous loading of RNAP (amber) and MCM DHs (blue) to the same DNA molecule (gray) as marked by white arrows.

(D–F) Representative kymographs showing recovery from transcription pausing (D), transcription stalling (E) or RNAP ejection (F) upon collision between RNAP (amber) and MCM DH (blue).

(G–I) Influence of MCM DHs on transcription pausing. Transcription pause probability (G), probability of recovery from pausing (H) and transcription pause duration (I) in the absence (0) or presence of (1 to ≥5) pushed MCM DHs. Only DNA molecules with one MCM foci located at ARS1 were analyzed. Bar plots display the mean and SEM. *Data displayed for 0 pushed MCM DHs in (G), (H), and (I) were combined with data shown in panels 2E, S2F, and S2G (ATP condition only), respectively.

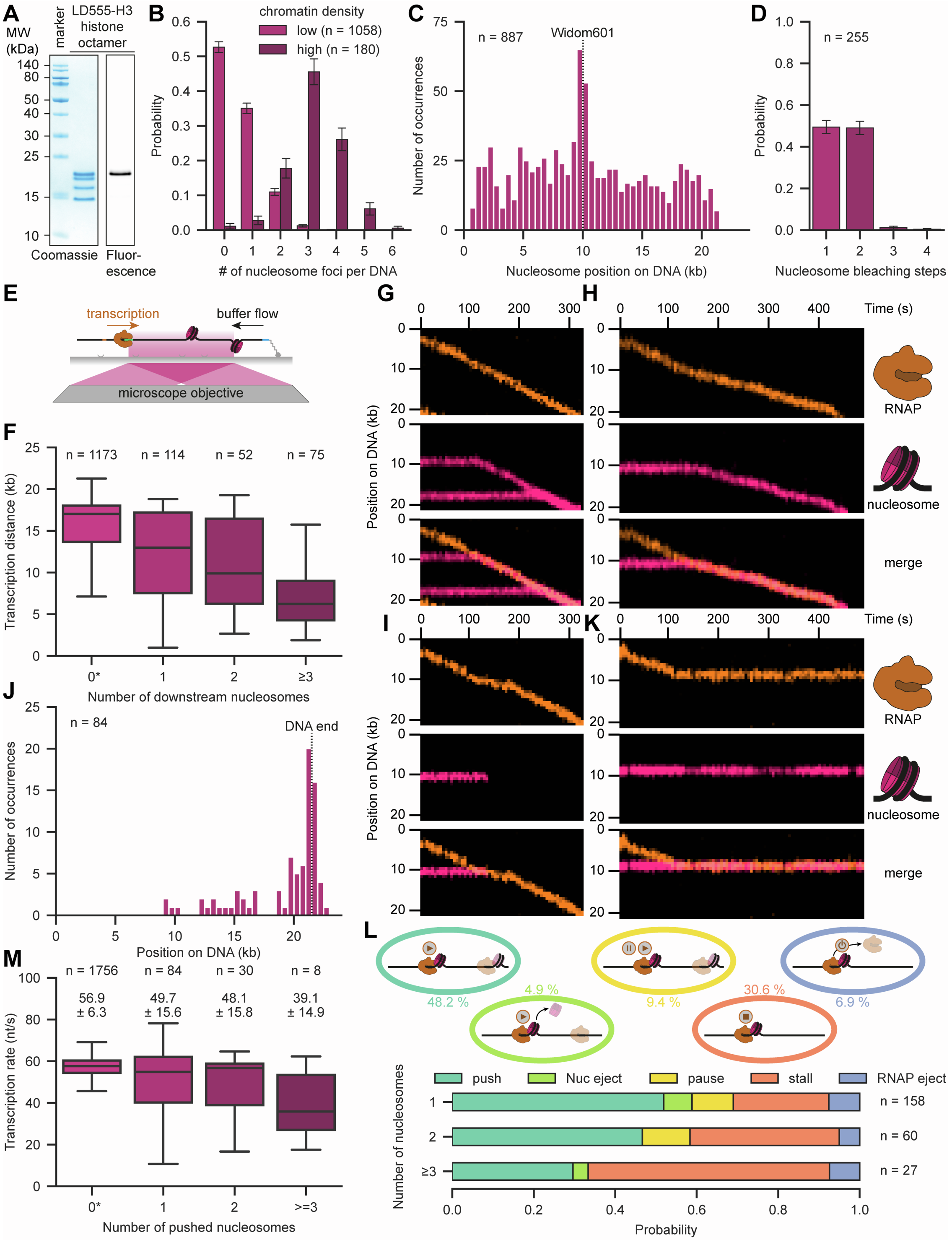


**Figure S4. RNA polymerase can transcribe through individual nucleosomes, Related to Figure 4**

(A) SDS-PAGE analysis of purified LD555-H3 histone octamers by Coomassie Blue staining and fluorescence detection.

(B) Number of nucleosome foci per DNA in low- high density chromatin.

(C) Nucleosome binding distribution histogram on T7P-ARS1-Widom601-DNA for low-density chromatin.

(D) Distribution of nucleosome bleaching steps in low-density chromatin.

(E) Schematic of the single-molecule transcription assay in the presence of nucleosomes. Time-coordinated transcription was set up as described in Figure 2A but on chromatinized DNA.

(F) Boxplot of the global transcription distance in the absence (0) or presence of (1 to ≥3) nucleosomes downstream of T7P. MCMs were not present on molecules analyzed.

(G–I) Representative kymographs demonstrating that RNAP (amber) could transcribe through one or multiple nucleosomes (pink) by nucleosome pushing (G and H) or ejection (I).

(J) Distribution of DNA positions of pushed nucleosomes after transcription stop. Only DNA molecules with one nucleosome foci were considered.

(K) Representative kymograph displaying transcription stalling upon collision between RNAP (amber) and nucleosome (pink).

(L) Quantification of the outcomes of RNAP collisions with a total of 1, 2 or ≥3 nucleosomes. Displayed percentages represent the combined probability irrespective of the number of nucleosomes.

(M) Boxplot of transcription rates in the absence (0) or presence of (1 to ≥3) pushed nucleosomes. Values above the box plots indicate the mean ± SD derived from a Gaussian fit. Bar plots in (B) and (D) display the mean and SEM. *Data displayed for 0 downstream and pushed nucleosomes in (F) and (M) were combined with data shown in Figure 2C and 2D, respectively.

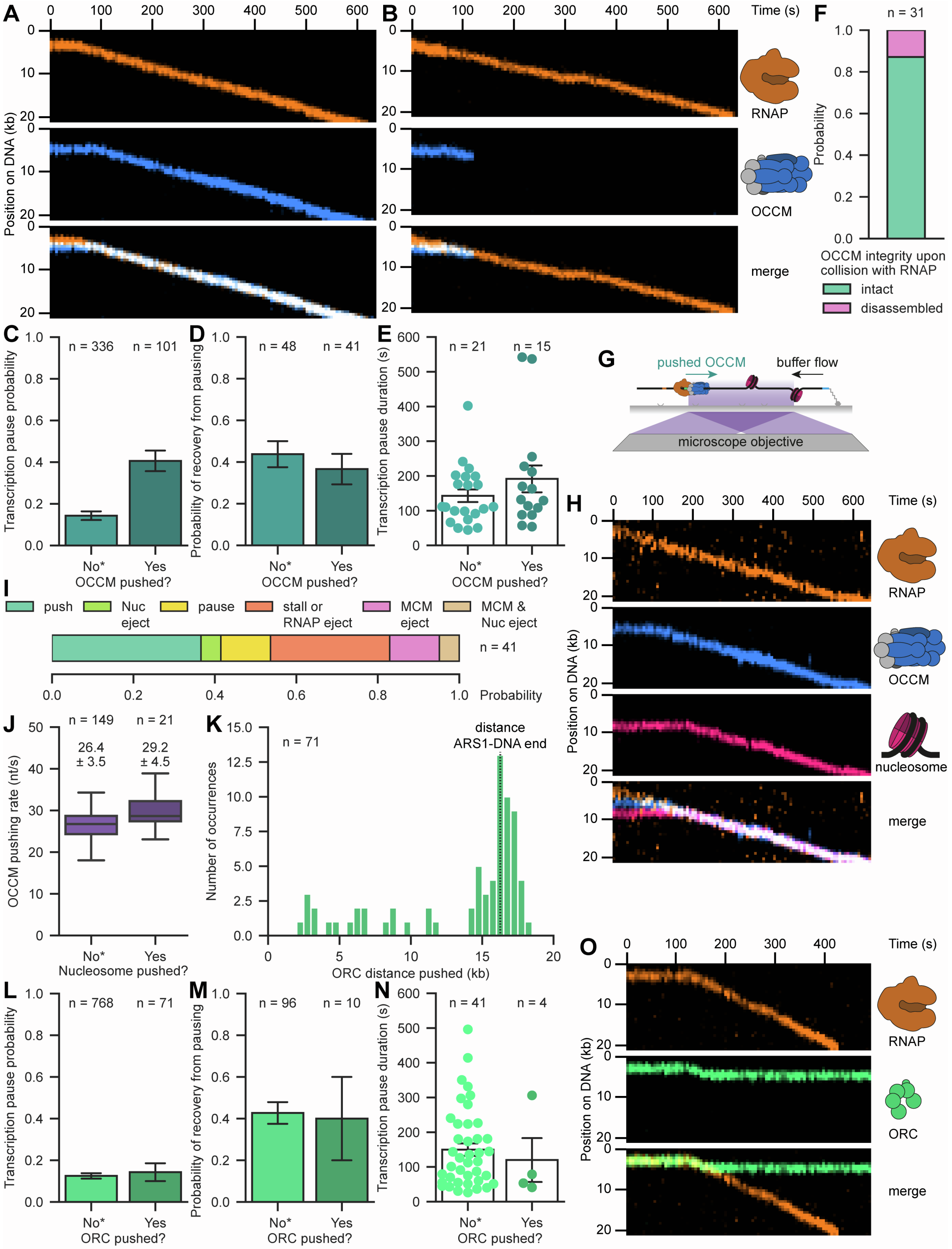


**Figure S5. Additional characterization of transcription-driven OCCM and ORC displacement, Related to Figure 5**

(A and B) Representative kymographs showing that RNAP (amber) could push OCCM (blue) upon collision (A) but OCCM was ejected in some cases (B).

(C–E) Influence of OCCM on transcription pausing. Transcription pause probability (C), probability of recovery from pausing (D) and transcription pause duration (E) in the absence or presence of OCCM. *Data displayed for non-pushed OCCM in (C), (D), and (E) were combined with data shown in Figure 2E, S2F, and S2G (ATPγS condition only), respectively.

(F) OCCM integrity upon collision with RNAP. Probability of OCCM staying intact or being disassembled as judged by the presence of ORC and Cdt1-MCM.

(G) Schematic of the single-molecule OCCM displacement assay in the presence of nucleosomes. OCCM was loaded and pushed by RNAP as described in Figure 5A but on chromatinized DNA.

(H) Representative kymograph demonstrating that RNAP (amber) could displace OCCM (blue) through nucleosomes (pink) by nucleosome pushing.
(I) Quantification of the outcomes of pushed OCCM collisions with nucleosomes.
(J) Boxplot of OCCM pushing rates in the absence or presence of nucleosomes. Values above the box plots indicate the mean ± SD derived from a Gaussian fit. *Data displayed for non-pushed nucleosomes were combined with data shown in Figure 5D.
(K) Distribution histogram of ORC distance pushed by RNAP.
(L–N) Influence of ORC on transcription pausing. Transcription pause probability (L), probability of recovery from pausing (M) and transcription pause duration (N) in the absence or presence of ORC. *Data displayed for no pushed ORC in (L), (M), and (N) were combined with data shown in Figure 2E, S2F, and S2G (ATP condition only), respectively.
(O) Representative kymograph displaying that ORC (green) which colocalized with RNAP (amber) could initially be pushed to ARS1 where it then was bypassed by RNAP. Bar plots in (C), (D), (E), (L), (M), and (N) display the mean and SEM.

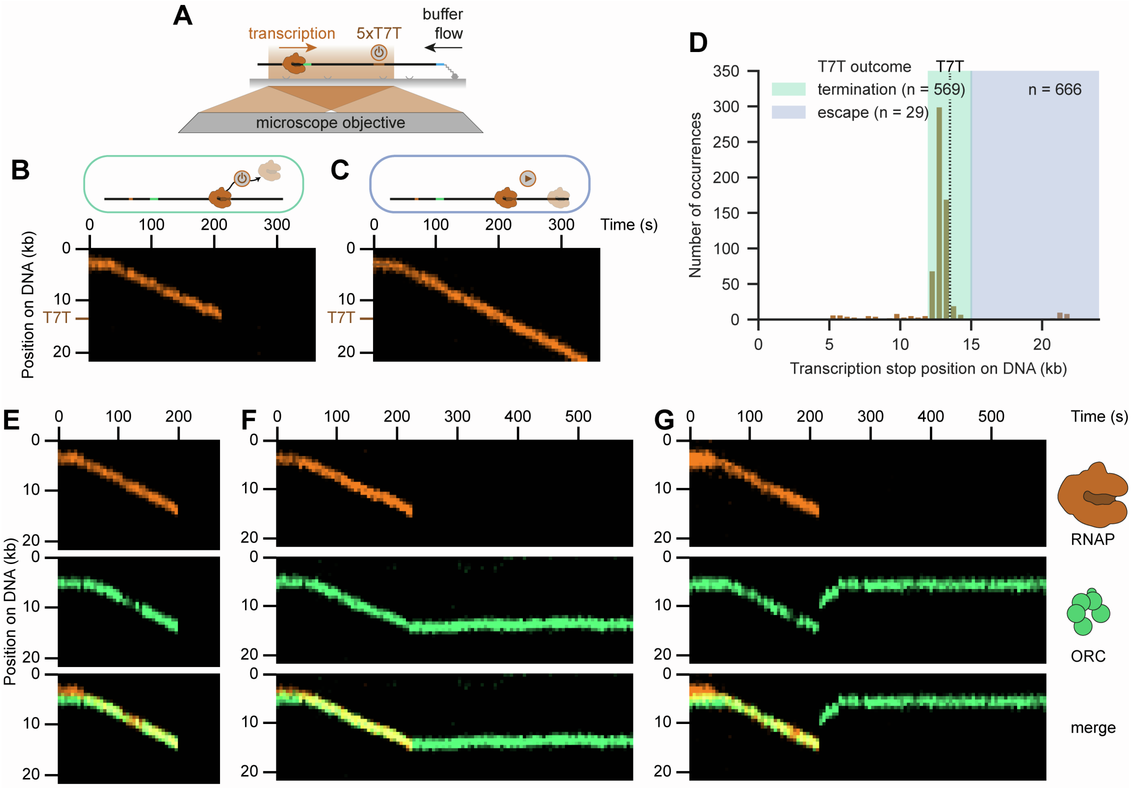


**Figure S6. ORC does not stably associate at random DNA sequences, Related to Figure 6**

(A) Schematic of the single-molecule transcription assay on 5xT7 terminator (T7T)-DNA. Time-coordinated transcription was set up as described in Figure 2A.

(B and C) Representative kymographs displaying transcription termination at the introduced 5xT7T site (B) with a small fraction escaping termination (C).

(D) Transcription is efficiently terminated by 5xT7T. RNAP transcription stop site distribution on 5xT7T-DNA.

(E–G) Representative kymographs showing that although the majority of displaced ORC (green) dissociated (E), ORC could remain stable at the 5xT7T site (F) or slide back to ARS1 after a 1D diffusion in search of the origin (G) upon RNAP (amber) transcription termination.

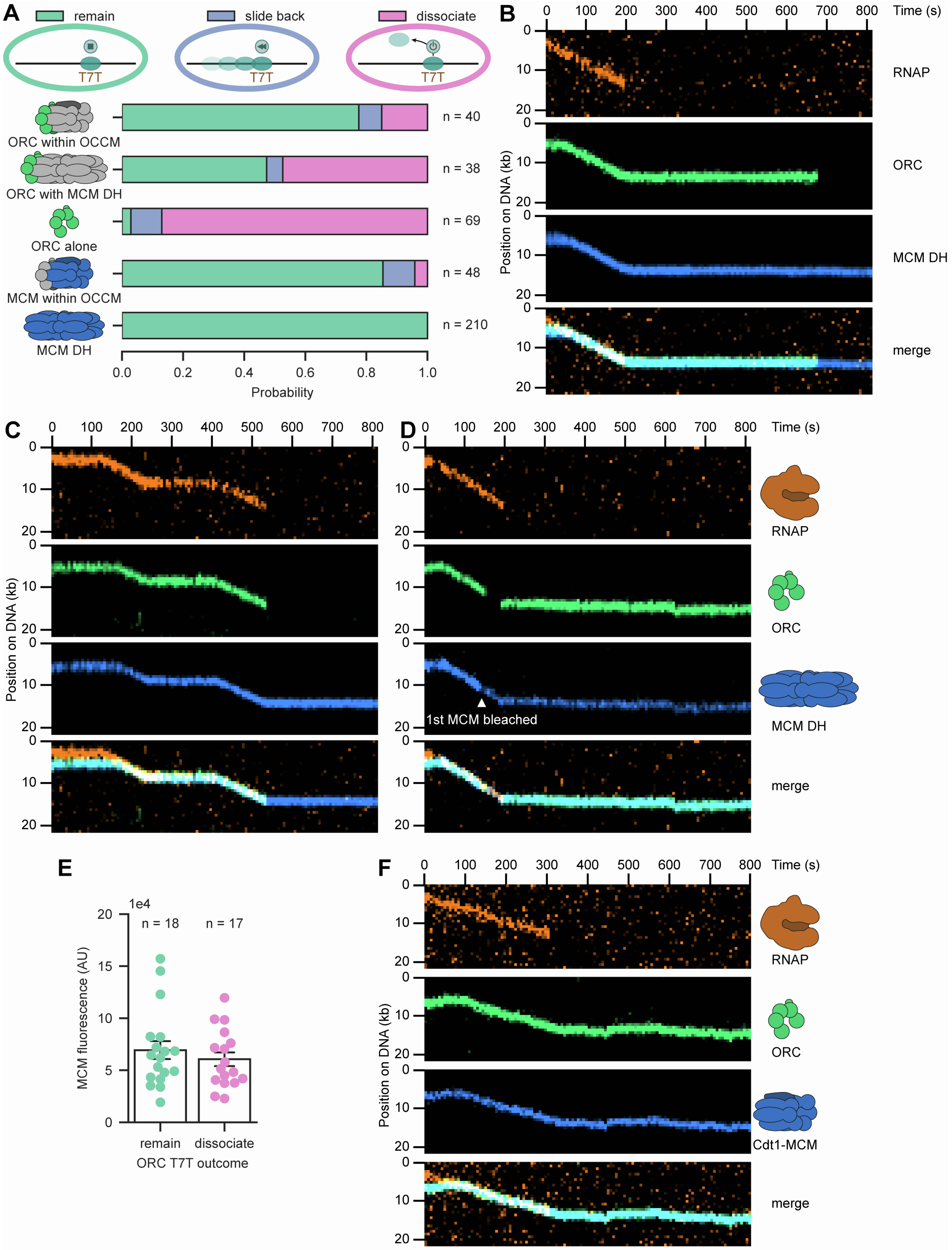


**Figure S7. MCMs stabilize ORC at new locations, Related to Figure 6**

(A) Quantification of the stability of displaced origin licensing factors at the 5xT7T site upon transcription termination.

(B–D) Representative kymographs showing that displaced MCM DHs (blue) always remained stable at the 5xT7T site while an associated ORC (green) could either remain stable (B and D) but also dissociate (C) upon RNAP (amber) transcription termination. The MCM signal and bleaching in (D) corresponds to an MCM DH and not an ORC-containing intermediate. MCM DHs were formed using ATP.

(E) Observed MCM fluorescence for MCM DHs associated with ORC molecules which remained or dissociated at the 5xT7T site upon transcription termination.

(F) Representative kymograph showing that displaced OCCM remained stable at the 5xT7T site, as judged by the presence of ORC (green) and Cdt1-MCM (blue), upon RNAP (amber) transcription termination. OCCM complexes were formed using ATPγS throughout the experiment.
