# Supplementary material for: Mobile origin-licensing factors confer resistance to conflicts with RNA polymerase
Source: Cell Rep. 2022 Mar 22;38(12):110531. doi: 10.1016/j.celrep.2022.110531 (PMC8961423; doi:10.1016/j.celrep.2022.110531)
Supplement: Document S2. Article plus supplemental information [file mmc8.pdf]

Cell Reports

Article

# Mobile origin-licensing factors confer resistance to conflicts with RNA polymerase

## Graphical abstract

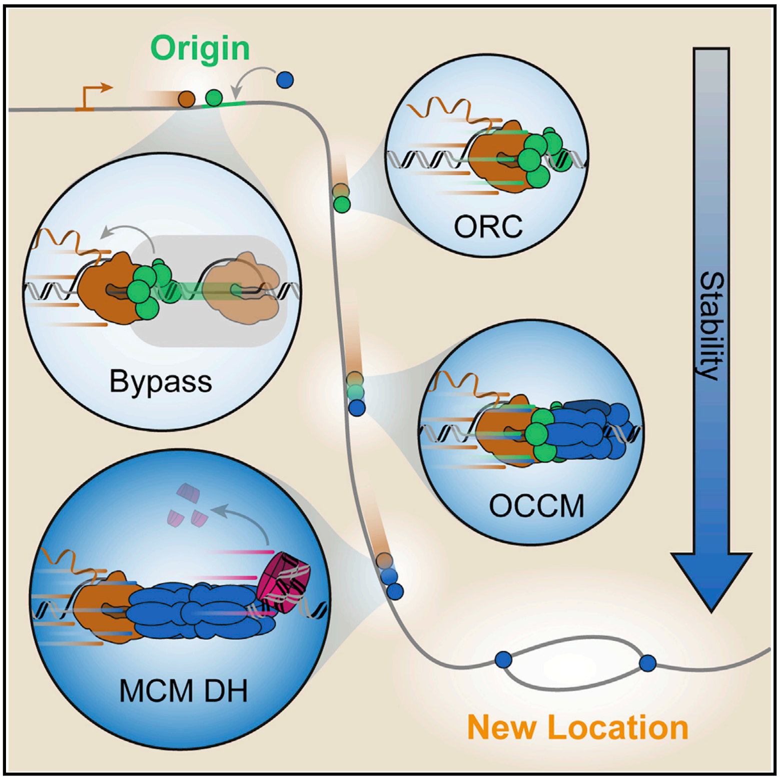


## Highlights

- RNA polymerase robustly pushes MCMs without slowing down
- Nucleosomes are ejected or pushed by MCMs during repositioning
- RNA polymerase can bypass ORC or activate ORC sliding and OCCM sliding
- Origin licensing continues after RNA polymerase encounters and relocalization

## Authors

Matthias J. Scherr, Syafiq Abd Wahab, Dirk Remus, Karl E. Duderstadt

## Correspondence

duderstadt@biochem.mpg.de

## In brief

Scherr et al. report a single-molecule imaging approach that provides a real-time view of encounters between RNA polymerase and replication origin-licensing factors. They show that origin-licensing factors can be pushed to new locations by RNA polymerase to support alternative origin specification pathways.

Scherr et al., 2022, Cell Reports *38*, 110531
March 22, 2022 © 2022 The Author(s).
https://doi.org/10.1016/j.celrep.2022.110531

CellPress

Cell Reports

CellPress
OPEN ACCESS

Article

# Mobile origin-licensing factors confer resistance to conflicts with RNA polymerase

Matthias J. Scherr,[1] Syafiq Abd Wahab,[2] Dirk Remus,[2] and Karl E. Duderstadt[1,3,4,*]
[1]Structure and Dynamics of Molecular Machines, Max Planck Institute of Biochemistry, Am Klopferspitz 18, 82152 Martinsried, Germany
[2]Memorial Sloan Kettering Cancer Center, Molecular Biology Program, 1275 York Avenue, New York, NY 10065, USA
[3]Physik Department, Technische Universität München, James-Franck-Straße 1, 85748 Garching, Germany
[4]Lead contact
*Correspondence: duderstadt@biochem.mpg.de
https://doi.org/10.1016/j.celrep.2022.110531

## SUMMARY

**Fundamental to our understanding of chromosome duplication is the idea that replication origins function both as sites where MCM helicases are loaded during the G1 phase and where synthesis begins in S phase. However, the temporal delay between phases exposes the replisome assembly pathway to potential disruption prior to replication. Using multicolor, single-molecule imaging, we systematically study the consequences of encounters between actively transcribing RNA polymerases (RNAPs) and replication initiation intermediates in the context of chromatin. We demonstrate that RNAP can push multiple licensed MCM helicases over long distances with nucleosomes ejected or displaced. Unexpectedly, we observe that MCM helicase loading intermediates also can be repositioned by RNAP and continue origin licensing after encounters with RNAP, providing a web of alternative origin specification pathways. Taken together, our observations reveal a surprising mobility in origin-licensing factors that confers resistance to the complex challenges posed by diverse obstacles encountered on chromosomes.**

## INTRODUCTION

All cellular life depends on the careful regulation of chromosome replication in space and time to ensure genome stability from one generation to the next. In eukaryotic cells, this process starts at origin sites located throughout chromosomes where the origin recognition complex (ORC), together with Cdc6 and Cdt1, loads the MCM helicase (Bleichert et al., 2017). To ensure chromosomes are replicated only once per cell cycle, loading and activation of MCM helicases is temporally separated into licensing and firing stages that take place during the G1 and S phases of the cell cycle, respectively (Siddiqui et al., 2013). The intricate sequence of conformational rearrangements performed during the loading process result in the formation of an MCM double-hexamer (MCM DH) encircling double-stranded DNA (dsDNA) (also known as the pre-RC (Evrin et al., 2009; Miller et al., 2019; Remus et al., 2009)) poised for activation by S-phase kinases and the formation of bidirectional replication forks.

The existence of origins, or specific genomic loci, that serve as start sites for replication is a cornerstone in our understanding of chromosome duplication, but defining universal characteristics of origins across the domains of life has proved challenging. The replicon model, which guided our initial understanding, postulated the existence of specific DNA sequence elements, termed replicators, that serve as start sites through engagement of an initiator protein (Jacob et al., 1963). Consistent with this model, highly refined DNA sequence elements have been discovered in bacterial origins (Bramhill and Kornberg, 1988; Kowalski and Eddy, 1989; Mackiewicz et al., 2004). In contrast to bacteria, eukaryotic origins are far more diverse and difficult to classify. In *Saccharomyces cerevisiae*, origins are largely defined by autonomously replicating sequence (ARS) elements, but in other eukaryotes no specific sequences have been found; instead, chromatin structure and post-translational modifications on histones are the defining features of origins (Prioleau and MacAlpine, 2016; Wang et al., 2021). Moreover, origin spacing and usage vary with changing demands throughout development in higher eukaryotes (Rausch et al., 2020). The large excess of MCMs compared with the number of origin sites further complicates classification and has become known as the MCM paradox (Donovan et al., 1997; Edwards et al., 2002). The observation of repetitive loading and spreading of MCMs from origin sites provides an explanation (Douglas et al., 2018; Edwards et al., 2002; Harvey and Newport, 2003; Powell et al., 2015); however, the mechanism of redistribution in the context of chromatin remains mysterious.

The temporal gap between licensing and firing provides a window of time during which dynamic events on the chromosome can influence origin specificity and replication initiation. Chromosome function critically depends on an ordered hierarchy of dynamic events over a broad range of time and length scales. To navigate the vast stretches of exposed chromatin and locate specific targets on the genome, factors use a combination of global diffusion interspersed with local sliding (Chen et al., 2014; Mirny et al., 2009). This trial-and-error search process involves more failure than success, resulting in frequent


This is an open access article under the CC BY license (http://creativecommons.org/licenses/by/4.0/).

non-specific encounters on short time scales. These events are essential for the assembly of macromolecular machineries, and the execution of complex regulatory programs on longer time scales, which in turn coordinate large-scale chromosome transformations, such as duplication, on time scales of cell cycle stages. Many questions remain about how these dynamic events, which are an intrinsic feature of chromosome life, influence the mechanics of origin licensing. Conflicts arising from transcription at sites of origin licensing have emerged as an acute example of how local dynamics can have potentially disastrous and far-reaching consequences for replication (Gros et al., 2015; Macheret and Halazonetis, 2018). Beyond the dynamics of individual factors, the rapidly evolving chromatin landscape can act to positively or negatively regulate these events (Azmi et al., 2017; Foss et al., 2019). Building a complete understanding and predictive models for how dynamic encounters regulate essential pathways, such as replication, depends on approaches that reveal the dynamics on the single complex level (Scherr et al., 2018).

The high temporal and spatial resolution offered by single-molecule approaches has helped to clarify the order of events and dynamic states formed during replication. Colocalization single-molecule spectroscopy assays with small ARS-containing substrates revealed that MCM hexamers are loaded sequentially as cracked rings, which subsequently close to form a final licensed double-hexamer (Ticau et al., 2015, 2017). Single-molecule imaging of long substrates has allowed for the spatial localization of replication intermediates relative to specific sequences and tracking of active and stalled replisomes (Duzdevich et al., 2015; Graham et al., 2017; Gruszka et al., 2020; Lewis et al., 2020; Sanchez et al., 2021; Sparks et al., 2019; Wasserman et al., 2019). These approaches have begun to reveal the dynamic intermediates that support robust replisome assembly and function. Nevertheless, how the order of events during origin licensing is altered during conflicts has remained unclear. In particular, there have been several demonstrations of relocation of replication start sites by transcription both *in vitro* and in cells (Gros et al., 2015; Macheret and Halazonetis, 2018; Powell et al., 2015), but the molecular pathways underlying these events have not been fully clarified.

We developed single-molecule assays to temporally and spatially resolve the dynamics of origin licensing during encounters with RNA polymerase (RNAP) in the context of chromatin. Unexpectedly, we observe that RNAP not only repositions MCM DHs, as suggested previously (Gros et al., 2015), but also can reposition the origin-licensing intermediates ORC and ORC-Cdc6-Cdt1-MCM (OCCM). Strikingly, in addition to relocation by pushing, RNAP is frequently able to bypass ORC. Bypass is specific to the ARS-bound conformation of ORC, with ORCs bound to random sequences rapidly repositioning upon encounters with RNAP. We further demonstrate that ORC and OCCM remain functional and continue origin licensing after these dynamic encounters with RNAP. Chromatin can positively or negatively regulate origin licensing (Azmi et al., 2017; Foss et al., 2019). Therefore, we evaluated how the chromatin landscape alters transcription resistance pathways. We observe that individual nucleosomes are readily repositioned, and three-way encounters involving RNAP, MCM DHs, and chromatin result in the formation of mobile supercomplexes that slow down only upon build-up of multiple nucleosomes. Taken together, our observations reveal a web of alternative origin specification pathways that confer an unexpected resilience to origin licensing. We propose that the mobility of origin-licensing factors confers stability to critical origin-licensing intermediates during encounters with diverse machineries operating on chromosomes.

## RESULTS

### MCM loading occurs at ARS1 and requires ATP hydrolysis

We developed a total internal reflection fluorescence (TIRF)-based single-molecule assay to directly visualize dynamic events during origin licensing in real time. Origin licensing was reconstituted using purified components in a stepwise manner (Figure 1A). First, 21-kb-long autonomously replicating sequence 1 (ARS1)-containing DNA molecules were immobilized with one end on the surface of functionalized coverslips via biotin-streptavidin-biotin interactions. Second, the purified licensing factors ORC, Cdc6, and Cdt1-MCM (Figure S1A) were introduced to load MCM onto dsDNA. Finally, the dynamics of licensing factors were temporally and spatially resolved on flow-stretched DNA molecules.

In the presence of ORC and Cdc6, fluorescently labeled MCMs readily colocalized with dsDNA with no detectable difference in loading efficiency compared with wild-type MCMs in ensemble assays (Figures S1B and S1C). Most MCM complexes loaded specifically at the replication origin ARS1, coincident with fluorescently labeled ORC, but a subpopulation of complexes was observed at alternative sites throughout the DNA (Figure 1B), in line with previous studies showing MCM loading in the absence of origin sequences *in vitro* (Gros et al., 2014; On et al., 2014). Robust loading of MCMs was verified by resistance to high-salt challenge, a characteristic of successful Cdt1 release and ring closure in conjunction with ATP hydrolysis (Evrin et al., 2009; Remus et al., 2009).

Despite still ongoing debates about the exact underlying mechanism of MCM DH formation, a loading intermediate of OCCM could be confirmed by various studies (Miller et al., 2019; Ticau et al., 2015; Yuan et al., 2017). The OCCM complex occurs prior to ATP hydrolysis, which triggers Cdc6 and Cdt1 release and, hence, stable association with dsDNA. To study OCCM dynamics, we performed MCM loading reactions in the presence of ATP$\gamma$S, halting origin licensing at the OCCM state. As expected, OCCM formation also occurs preferentially at ARS1 (Figure S1D); however, observed loading efficiencies were reduced (Figures 1C, S1B, and S1C). In line with previous studies (Evrin et al., 2009; Remus et al., 2009), only a small fraction of OCCM complexes survived high-salt challenge, confirming the incomplete loading of MCM around DNA in the OCCM (Figures 1C, S1B, and S1C).

Interestingly, besides the expected single MCM DH per origin, we also detect populations composed of two or more MCM DHs at the origin in the presence of ATP (Figures 1D and S1E). To rule out the possibility of either non-specific MCM oligomerization or parallel, independent loading events around ARS1, we performed the same experiment in the presence of ATP$\gamma$S. Strikingly, we almost exclusively observed single-step

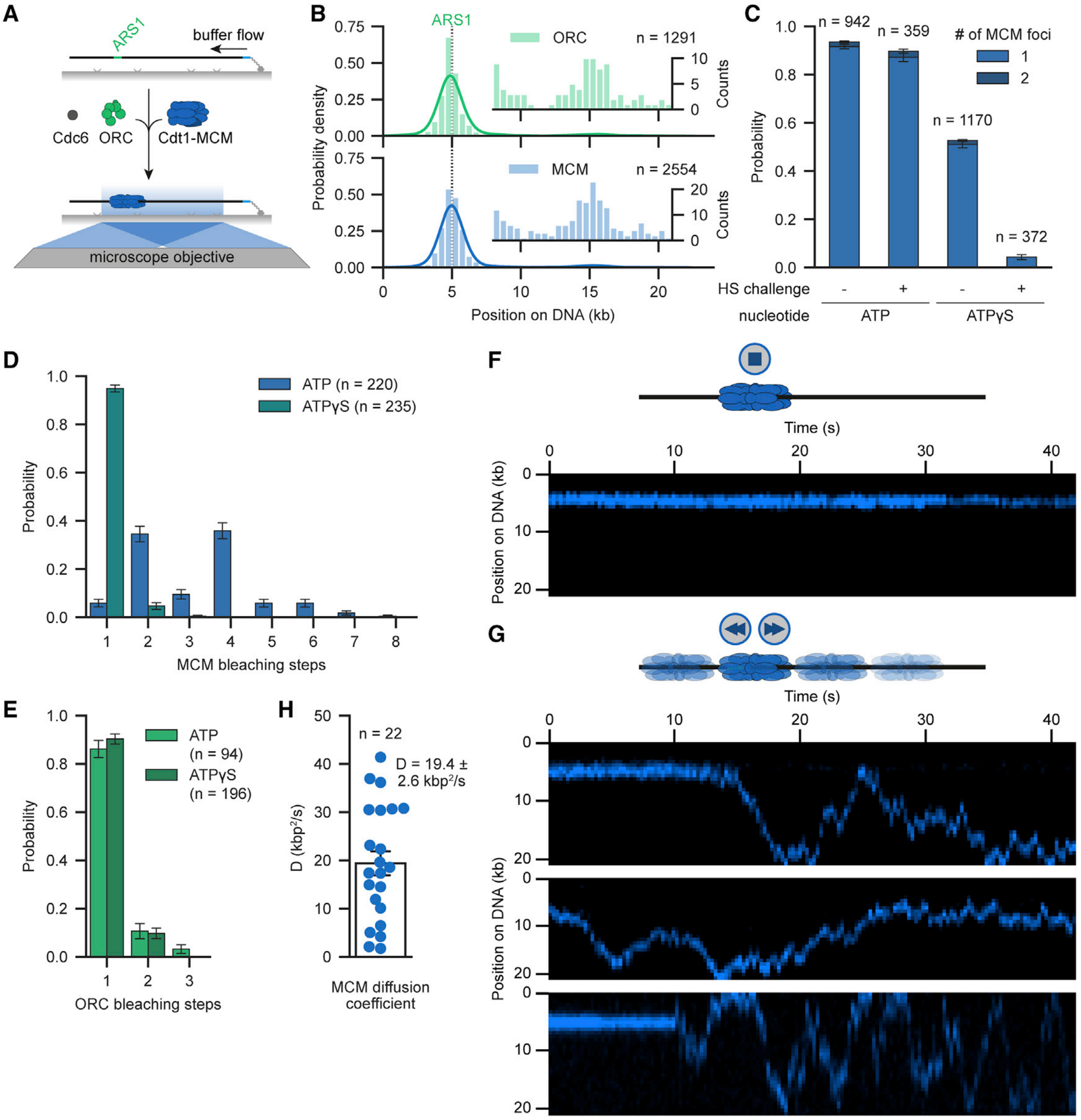


**Figure 1. MCM DHs load at ARS1 and can switch to a diffusive DNA binding mode**

(A) Schematic of the single-molecule helicase loading assay. ARS1-DNA (21 kb) was incubated with ORC, Cdc6, and Cdt1-MCM, washed, and imaged on flow-stretched DNA.

(B) ORC (green) and MCM (blue) binding distribution histogram on ARS1-DNA. Data from all experiments irrespective of ATP or ATPγS are shown. Lines represent the kernel density estimation (KDE). Insets show a close-up of residual binding downstream of ARS1. Results with ORC and MCM labeled are displayed (top). Merged results with labeled MCM and a combination of labeled and unlabeled ORC are displayed (bottom).

(C) Number of MCM foci on ARS1-DNA challenged with low or high salt (HS) after helicase loading in the presence of ATP or ATPγS.

(D and E) Distribution of MCM (D) and ORC (E) bleaching steps after helicase loading in the presence of ATP or ATPγS. ORC and MCM were both labeled.

(F and G) Representative kymographs showing MCM DH dynamics at 0.5 M NaCl. Most MCM DHs remained bound to ARS1 (F), but a subset switched to a diffusive DNA binding mode (G). MCM was labeled and ORC was unlabeled.

(H) MCM diffusion coefficients at 0.5 M NaCl. Bar plots in (C), (D), (E), and (H) display the mean and SEM. See also Figure S1.

photobleaching, consistent with the formation of a single OCCM per origin (Figures 1D and S1E). Single-step photobleaching was detected for ORC in both conditions (Figure 1E) with no correlation (pearson r = 0.012) between MCM and ORC bleaching (Figures S1F–S1H) in the ATP condition. Together, these data support a model in which a single ORC is competent for MCM DH formation (Gupta et al., 2021; Ticau et al., 2015) and potentially also for multiple rounds of MCM loading.

## MCM DH can switch to a diffusive DNA binding mode

Our data suggest that upon completion of origin licensing an MCM DH can be repositioned to permit the loading of an

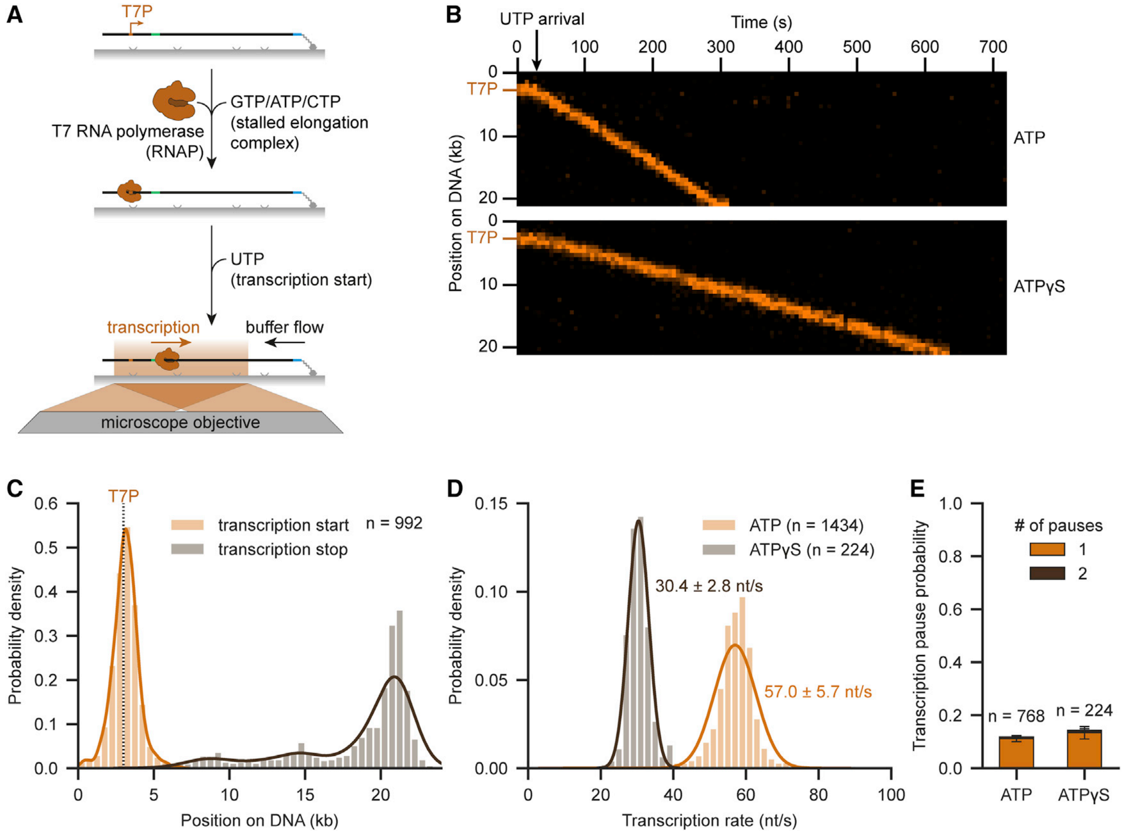


**Figure 2. Time-coordinated single-molecule transcription**

(A) Schematic of the time-coordinated transcription assay. Stalled T7 RNA polymerase (RNAP) elongation complexes were formed on 21 kb T7 promoter (T7P)-DNA in the presence of GTP, ATP, and CTP. Transcription was started and imaged after addition of UTP.

(B) Representative kymographs showing transcribing RNAP upon UTP arrival in the presence of ATP (top) or ATPγS (bottom).

(C) RNAP transcription start and stop site distribution histogram on T7P-DNA. Data from all experiments irrespective of ATP or ATPγS are shown. Lines represent the KDE.

(D) Transcription rate distribution in the presence of ATP or ATPγS. Values indicate the mean ± SD derived from a Gaussian fit (lines).

(E) Mean transcription pause probability on T7P-DNA in the presence of ATP or ATPγS. Error bars display SEM. See also Figure S2.

additional MCM DH at the origin (Figure 1D). EM studies have shown the formation of trains of MCMs on DNA under conditions that favor multiple loading events at or outside the origin (Douglas et al., 2018; Hill et al., 2020; Remus et al., 2009). Additionally, recent studies have also detected an ability of the Cdc45-MCM-GINS (CMG) helicase complex to slide along DNA (Douglas et al., 2018; Wasserman et al., 2019). Nevertheless, the dynamics of MCM diffusion have not been extensively characterized owing to few direct observations.

Remarkably, we observed very rare spontaneous sliding events at the completion of origin licensing under normal buffer conditions. To eliminate the influence of external forces, we tethered DNA at both ends in an orientation-specific manner to image MCM mobility in the absence of buffer flow for extended periods (Figure S1I). Whereas the majority of MCMs remained stationary (Figure 1F), continuous exposure to high salt increased the fraction of sliding MCMs to 1% (Figures 1G and S1J; Video S1) (Remus et al., 2009). We observed a wide range of MCM velocities from 2 to 40 kbp$^2$/s with a mean of 19.4 ± 2.6 kbp$^2$/s (±SEM; Figures 1G and 1H), as determined by linear extrapolation of calculated MSD (Figure S1K). Notably, MCM sliding under these conditions is an order of magnitude faster than that observed for CMG, albeit at lower ionic strength, but comparable that of other dsDNA-encircling proteins when exposed to high salt (Davidson et al., 2016; Stigler et al., 2016; Wasserman et al., 2019). We note that a slower, short-range diffusive mode of MCM DHs has also been reported by using optical tweezers, providing a comprehensive picture of these dynamics (Sanchez et al., 2021).

## Time-coordinated single-molecule transcription

To directly evaluate the consequences of encounters between RNAP and MCM DHs, we developed a time-coordinated, single-molecule transcription assay (Figure 2A). To this end, we introduced a promoter sequence, with the first thymidine occurring at position +16, upstream of ARS1, to permit initiation and formation of a single, stalled transcription elongation complex in the absence of UTP (Yin and Steitz, 2002). Site-specifically labeled T7 RNAP (AF488-, LD555-, and LD655-T7 RNAP; Figure S2A), engaged with DNAs containing the promotor in the absence of UTP in ensemble assays, confirming formation of stable elongation

complexes (Figure S2B). In our single-molecule setup, the majority of RNAP foci contained one molecule (Figure S2C) bound to the T7 promoter (Figures 2A and 2C). Upon addition of UTP, RNAP transcribed highly processively to the opposite DNA end (Figures 2B and 2C). We observed a mean transcription rate of 57.0 ± 5.7 nt/s, which is well in line with previous studies (Figure 2D) (Thomen et al., 2008).

Next, we evaluated the influence of ATPγS, required for OCCM formation, on transcription kinetics. Since synthesis depends only on hydrolysis of the beta bond, we did not expect disruption of activity when replacing ATP with ATPγS. However, the structural differences reduced the rate by ~50% (30.4 ± 2.8 nt/s; Figures 2B and 2D), but high processivity was observed for both ATP and ATPγS (Figures 2E and S2D). Pausing occurred sequence independently, and ~40% of molecules recovered with a mean duration of 146.1 ± 13.6 s (Figures S2E–S2H). Notably, all labeled RNAPs generated showed a similar behavior in terms of processivity, rate, and pausing in our experiments (Figures S2I–S2K).

## RNAP can robustly reposition MCM DHs

To directly visualize and determine the different outcomes of encounters between individual RNAPs and MCM DHs, we performed ensemble and single-molecule transcription experiments on licensed DNA with labeled RNAP and MCM (Figures 3A and S3A–S3C). Remarkably, approximately four out of five collisions between RNAP and MCM DHs resulted in robust repositioning, with most MCM DHs being displaced more than 10 kb to the DNA end (Figures 3B–3D). Nevertheless, we observed additional collision outcomes, including transcription pausing (1.4%) or stalling (3.5%) and RNAP ejection (13.8%) (Figures 3D and S3D–S3F). We never observed MCM ejection, showing the high robustness of the origin-licensing pathway and MCM DHs. Moreover, a preceding high-salt challenge did not change collision outcomes, demonstrating that MCM DHs loaded under physiological conditions are inherently prone to slide (Figure 3D).

Several studies, including our data above (Figure 1), suggest that multiple MCM DHs can be loaded at an origin. We, therefore, asked whether numerous MCM DHs might serve as a barrier for transcription using conditions that favor multiple MCM loading at non-ARS1 sites (see STAR Methods). Surprisingly, even multiple MCM DHs could be repositioned by a single RNAP (Figure 3E; Video S2). Transcription rates were not altered in the presence of a single MCM or MCM DHs and only reduced by ~20% with five or more MCM DHs (Figure 3F). Notably, the numbers of MCMs in each population reported should be considered an underestimate due to a small fraction of unlabeled and photobleached MCMs. Although transcription was more prone to pausing during encounters with multiple MCM DHs, the probability of recovery and duration remained unchanged (Figures S3G–S3I) compared with encounters with single MCMs at ARS1.

## RNAP can reposition MCM DHs together with multiple nucleosomes

We demonstrated that MCM DHs can be pushed onto bare DNA to facilitate transcription through licensed origins. To investigate MCM displacement in a more physiological context, we sought to directly observe MCM displacement in the presence of chromatin. We introduced a Widom601 nucleosome-positioning sequence into our DNA template and reconstituted chromatin using fluorescently labeled histone octamers at two different densities (low and high; Figures S4A and S4B). Low-density chromatin substrates with well-separable nucleosome foci revealed an enrichment of nucleosomes at the Widom601 sequence (Figure S4C). Furthermore, individual nucleosome foci showed one- and two-step photobleaching, consistent with correctly formed nucleosomes at our labeling efficiency (Figure S4D).

Next, we performed transcription on chromatin substrates in the presence or absence of pushed MCM DHs in front of RNAP (Figures 4A and S4E). Independent of MCM DH being in front of RNAP, we observed a similar global reduction in transcription distance with increasing number of downstream nucleosomes (Figures 4B and S4F). Collisions between RNAP and individual nucleosomes revealed continuous transcription through chromatin by either pushing or ejecting nucleosomes (Figures S4G–S4I), consistent with previous studies observing short-range transcription through nucleosomes (Hodges et al., 2009; O'Neill et al., 1993; Studitsky et al., 1994; Studitsky et al., 1997). Remarkably, individual nucleosomes were readily pushed over long distances to the DNA end (Figure S4J). However, roughly half of RNAP-nucleosome collisions resulted in RNAP ejection (6.9%), recovery from pausing (9.4%) or permanent stalling (30.6%) with the latter being increased by multiple nucleosomes (Figures S4K and S4L).

Individual nucleosomes did not present an obstacle for MCMs, as RNAP could also readily push MCM DHs through nucleosomes by nucleosome ejection or pushing (Figures 4C–4E; Video S3). Pushed MCM DHs were not affected by nucleosomes in about one-half the cases, whereas MCM pushing also paused transiently (11.1%) or stalled permanently (36.6%), similar to collisions between RNAP itself and nucleosomes (Figures 4F, 4G, and S4L). Importantly, collisions between MCM DHs and nucleosomes never triggered MCM unloading, again demonstrating the high robustness of the origin-licensing pathway. However, MCM repositioning was obstructed by larger numbers of nucleosomes with increased probability of stalling, but again not unloading (Figure 4G). RNAP-MCM complex rates remained mostly unaffected upon pushing individual nucleosomes with no slowly transcribing population (compare 0-25% quartiles in Figures 4H and S4M). Conversely, we observed a population of RNAP molecules (0-25% quartile) with substantially decreased transcription rates upon pushing individual nucleosomes (Figures S4H and S4M). Thus, our observations suggest that the DNA extruding from MCM DHs is more suitable for RNAP or that the absence of a slow transcription population could result from MCM DHs helping to destabilize nucleosomes.

## OCCM and ORC are repositioned or bypassed by RNAP

The remarkable resilience of MCM DHs during encounters with RNAP led us to wonder if other stages of the loading pathway might exhibit the same properties. MCM DH formation is a slow process with long-lived intermediates containing only one MCM prior to recruitment of the second MCM (Miller et al., 2019; Ticau et al., 2015). Thus, encounters between RNAP and loading intermediates are likely to occur during origin licensing.

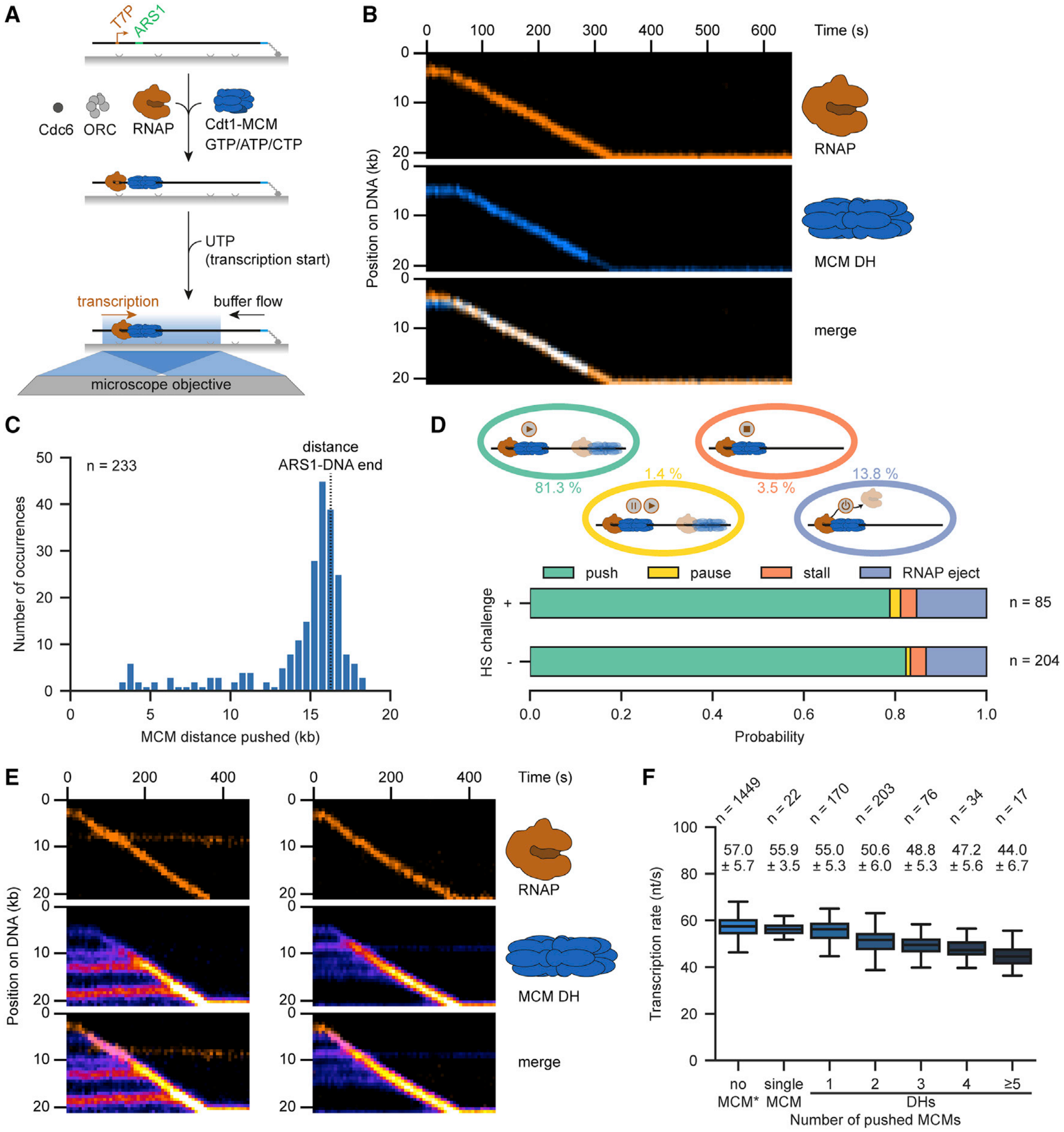


**Figure 3. RNAP can robustly reposition MCM DHs**

(A) Schematic of the RNAP-MCM DH collision assay. RNAP and MCM DHs were loaded onto 21-kb T7P-ARS1-DNA. Transcription was started and imaged after addition of UTP.

(B) Representative kymograph demonstrating that RNAP (amber) could push MCM DH (blue) upon collision.

(C) Distribution of MCM DH distance pushed by RNAP.

(D) Quantification of the outcomes of RNAP collisions with MCM DHs with (top) or without (bottom) a high-salt (HS) wash prior to transcription start. Displayed percentages represent the combined probability of both conditions.

(E) Representative kymographs demonstrating that RNAP (amber) can push multiple MCM DHs (fire LUT) over long distances.

(F) Boxplot of transcription rates in the absence (no MCM) or presence of a single MCM and (1 to ≥5) pushed MCM DHs. Values above the boxplots indicate the mean ± SD derived from a Gaussian fit. *Data displayed for no pushed MCMs were combined with data shown in Figure 2D. See also Figure S3.

To examine encounters between RNAP and the less stable OCCM intermediate, we performed MCM loading and transcription entirely in the presence of ATPγS (Figure 5A). Remarkably, two out of three RNAP-OCCM collisions led to robust OCCM repositioning (Figures 5B, 5C, and S5A). However, besides transcription pausing and stalling, a small fraction of OCCM was ejected (6.6%), which was never observed for MCM DHs (Figures 3D, 5B, and S5B). Although the OCCM complex appeared less stable than a MCM DH, we observed a higher probability of RNAP ejection upon collision with an OCCM (23.2%). Thus, OCCM might engage more strongly with ARS1, or ORC may present a greater obstacle to RNAP (Candelli et al., 2018).

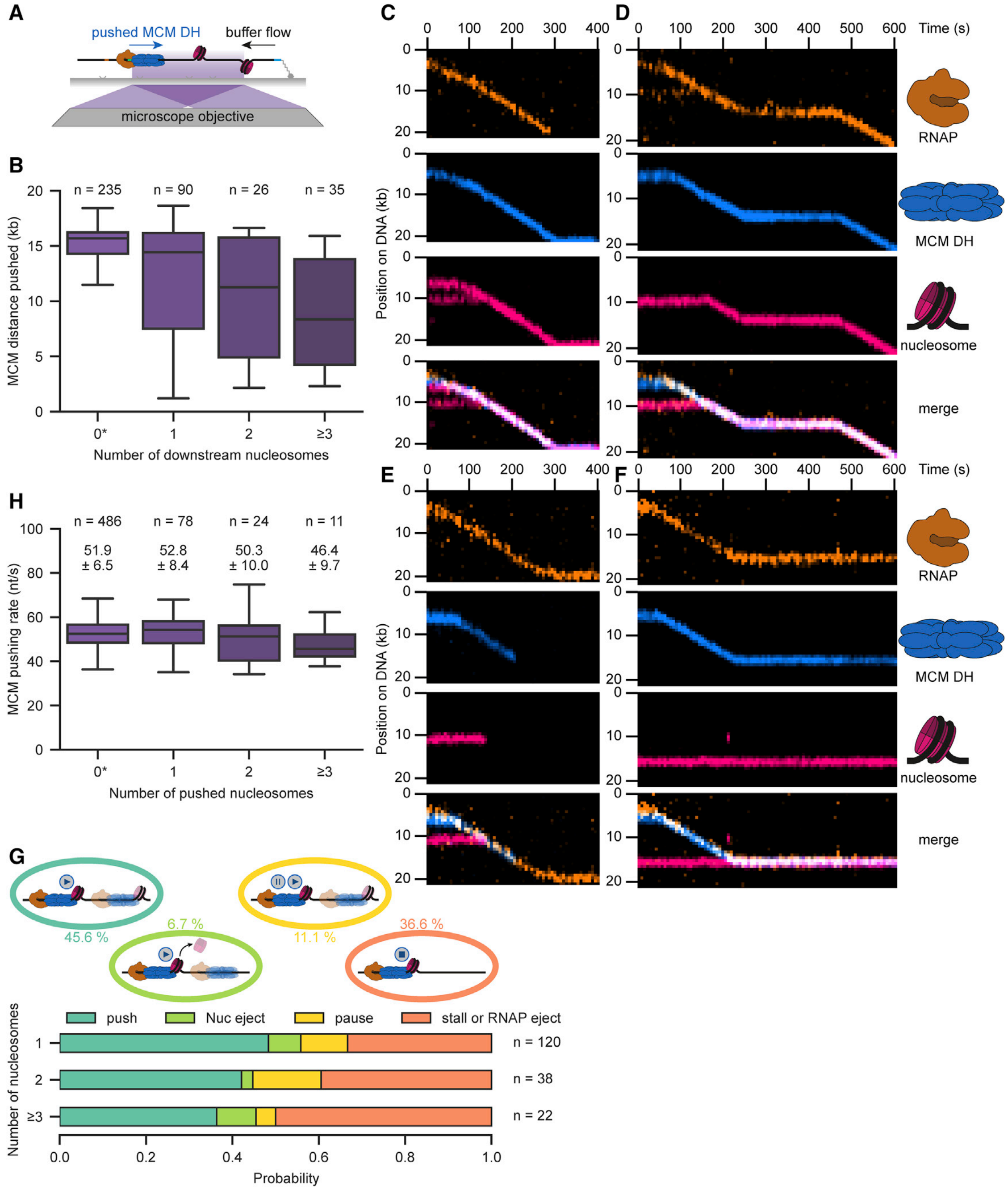


**Figure 4. RNAP can reposition MCM DHs together with multiple nucleosomes**

(A) Schematic of the MCM DH displacement assay in the presence of nucleosomes. Assay was performed as described in Figure 3A but on chromatinized DNA.

(B) Boxplot of the global MCM distance pushed in the absence (0) or presence (1 to ≥3) of nucleosomes downstream of ARS1.

(C–F) Representative kymographs demonstrating that RNAP (amber) could displace MCM DHs (blue) through one or multiple nucleosomes (pink) by nucleosome pushing (C and D) or ejection (E) besides transcription stalling (F) upon collision.

(G) Quantification of the outcomes of pushed MCM DH collisions with a total of 1, 2, or ≥3 nucleosomes. Displayed percentages represent the combined probability irrespective of the number of nucleosomes.

(H) Boxplot of MCM pushing rates in the absence (0) or presence of (1 to ≥3) pushed nucleosomes. Values above the boxplots indicate the mean ± SD derived from a Gaussian fit. *Data displayed for 0 pushed nucleosomes in (B) and (H) were combined with data shown in Figures 3C and 3F, respectively. See also Figure S4.

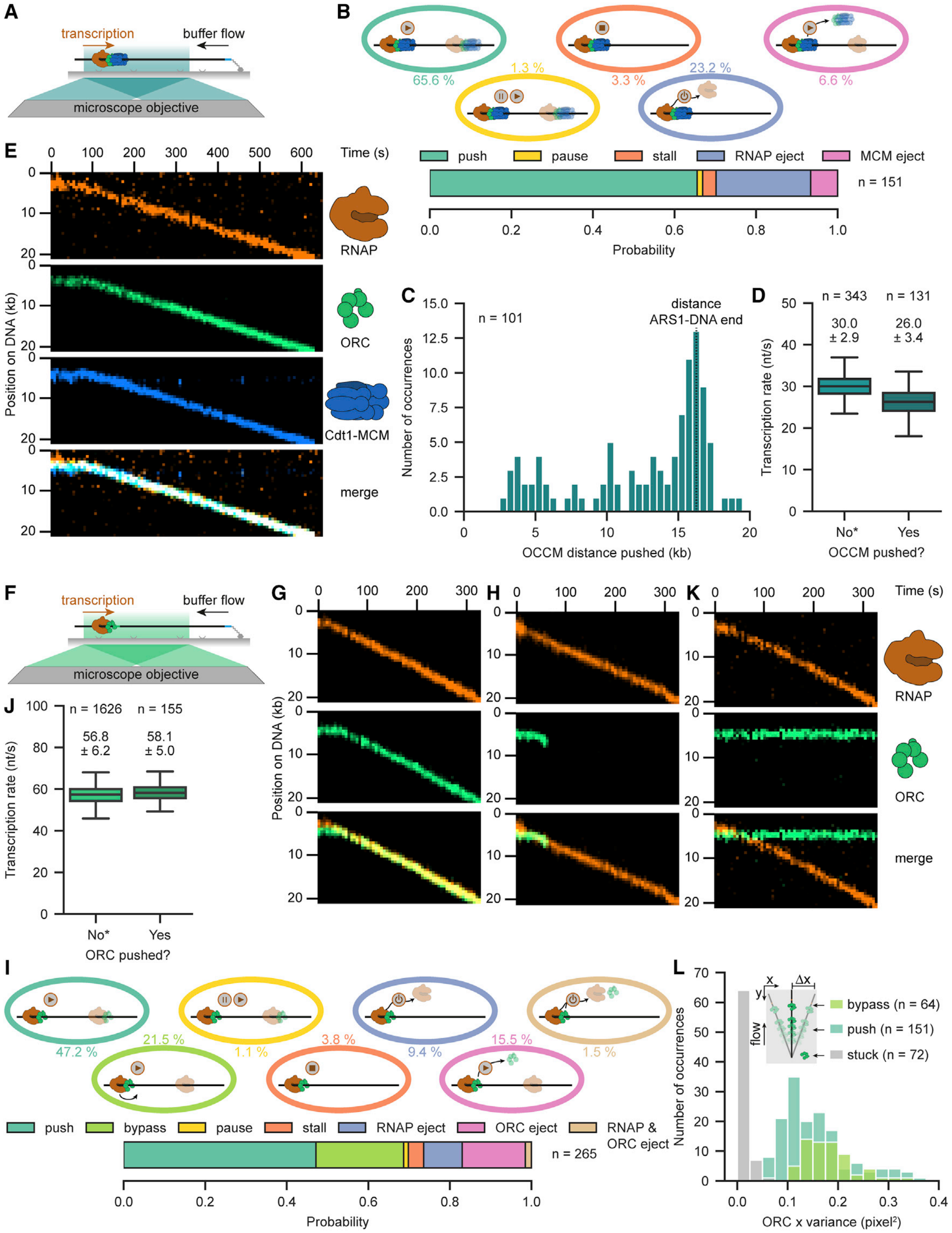


**Figure 5. OCCM and ORC are repositioned or bypassed by RNAP**

(A) Schematic of the RNAP-OCCM collision assay. Assay was performed as described in Figure 3A, but ATPγS was used instead of ATP in all steps.

(B) Quantification of the outcomes of RNAP collision with OCCM.

(C) Distribution histogram of OCCM distance pushed by RNAP.

(D) Boxplot of transcription rates in the absence or presence of a pushed OCCM.

(E) Representative kymograph demonstrating that OCCM stays intact when being pushed by RNAP (amber), as judged by the presence of ORC (green) and Cdt1-MCM (blue).

(legend continued on next page)

CellPress
OPEN ACCESS

Interestingly, pausing was almost three times more frequent when a single OCCM was pushed, comparable to three or more pushed MCM DHs (Figures 5D, S3G, and S5C). Pausing properties in terms of recovery and durations remained similar (Figures S5D and S5E). Although we demonstrated that OCCM complexes are also robustly displaced by RNAP, OCCM integrity was judged only by the presence of MCM. To exclude the possibility of OCCM disassembly, we additionally monitored ORC in three-color experiments containing labeled RNAP, ORC, and MCM. The majority of OCCM complexes stayed fully intact during encounters with RNAP and during displacement (Figures 5E and S5F). We also tested collisions between pushed OCCMs and nucleosomes (Figure S5G). Although around 17% of collisions led to OCCM loss, the majority stayed intact and were displaced (Figures S5H–S5J).

We next investigated the influence of transcription on ORC (Figure 5F). Surprisingly, although ORC is not topologically bound to DNA (unlike OCCM and MCM DH) and thus ejected more frequently (~17%), RNAP could also push ORC in almost one-half of RNAP-ORC collisions, predominantly to the DNA end (Figures 5G–5I and S5K; Video S4). Consistent with ORC being less stable, especially at non-ARS1 sites, transcription in the presence of ORC was not altered in terms of rate, pausing probability, and pausing properties (Figures 5J and S5L–S5N).

Unexpectedly, besides transcription pausing and stalling and RNAP ejection, we observed a major fraction (21.5%) of RNAPs bypassing ORC (Figures 5I and 5K; Video S5). Although we never observed bypassing for collisions between RNAP and MCM DHs, OCCM, or nucleosomes, RNAP-ORC bypass could represent an artifact by surface-stuck ORC or overlapping DNA. To exclude this possibility, we determined the variance in x direction (perpendicular to buffer flow) of stuck (not colocalizing with DNA), pushed, and bypassed ORC molecules. As expected, pushed and bypassed ORC show an order of magnitude higher mean variance (0.156 pixel$^2$) caused by DNA fluctuations than surface-stuck ORC molecules (0.015 pixel$^2$), demonstrating that bypassed ORCs are in fact bound to DNA (Figure 5L). A small fraction of ORC failed to stably engage with ARS1 but instead colocalized with RNAP at the T7 promoter. Interestingly, RNAP pushed ORCs in this fraction to ARS1 and subsequently bypassed them, demonstrating that ORC bypass occurs on the same DNA molecule (Figure S5O). Together, we demonstrate that origin-licensing intermediates OCCM and ORC are resilient to conflicts with RNAP by displacement or bypass mechanisms.

## Origin licensing continues after RNAP encounters

The unexpected stability of origin-licensing intermediates and their ability to be pushed or bypassed upon encountering transcribing RNAP offers many potential pathways for origin specification. To investigate these possibilities, we first tested whether displaced licensing factors remained after termination of transcription. To this end, we introduced five tandem T7 terminators (T7T) downstream of ARS1 (Figure S6A). As expected, transcription efficiently terminated at T7T (~95%), with only a small fraction escaping termination (Figures S6B–S6D). Next, we investigated the stability of displaced ORC at T7T. Consistent with previous reports that ORC does not stably associate with regions containing random DNA sequences (Duzdevich et al., 2015; Sanchez et al., 2021), we found that the majority of ORC molecules dissociated upon transcription termination (87%; Figures S6E and S7A). Nevertheless, a small population of molecules either remained near the termination site (3%; Figure S6F) or were pushed by flow back to the origin and rebound (Figure S6G).

In cells, we expect ORC diffusion to be limited and, hence, ORC to reside at alternative sites only through stabilization by other factors. Thus, we tested whether MCM DHs could stabilize associated ORC. MCM DHs always remained stable at T7T, in line with studies showing successful MCM DH activation at non-origin sites (Figures S7A–S7C) (Gros et al., 2015). Surprisingly, ORC associated with MCM DHs was far more stable, with equal populations remaining and dissociating at T7T. This indicates that ORC could piggyback to new locations on MCM DHs. We cannot fully exclude the possibility that the ORC that remained stable at T7T was part of a licensing intermediate (e.g., OCCM or MO complex). However, photobleaching was consistent with MCM DHs, and we detected no difference in mean MCM fluorescence between populations with remaining or dissociating ORC (Figures S7D and S7E). Finally, we determined the stability of the OCCM intermediate at T7T. The majority of OCCM complexes stayed intact and remained stably bound at T7T (78% and 85% for ORC and Cdt1-MCM, respectively; Figures S7A and S7F; Video S6).

To investigate whether ORC and OCCM complexes that survive encounters with RNAP remain functional and continue origin licensing, we introduced fresh Cdc6 and Cdt1-MCM having a different fluorescent label together with ATP to the products of the transcription collision experiments. Importantly, we used a DNA construct containing a second replication origin introduced right next to a single T7T. First, we evaluated OCCM complexes given their higher stability (Figure 6A). Since, to our knowledge, there have been no reports demonstrating conversion of ATPγS-stalled OCCM to MCM DH, we started by examining the fraction of molecules having no active transcription. We did not observe two-color MCM foci (1/229 molecules) as we would predict if OCCMs directly converted to MCM DHs by recruitment of new MCMs. We attribute this lack of conversion to the use of ATPγS to generate the stalled OCCM complexes. Nevertheless, to our

(F) Schematic of the RNAP-ORC collision assay. Assay was performed as described in Figure 3A, but only RNAP and ORC were loaded on DNA.
(G and H) Representative kymographs showing that ORC (green) could also be displaced by RNAP (amber, G) but was ejected more frequently (H).
(I) Quantification of the outcomes of RNAP collision with ORC.
(J) Boxplot of transcription rates in the absence or presence of a pushed ORC.
(K) Representative kymograph demonstrating that RNAP (amber) was able to bypass ORC (green) bound to ARS1.
(L) Distribution of x variance perpendicular to buffer flow for pushed, bypassed, and surface-stuck ORC molecules. Values above the boxplots in (D) and (J) indicate the mean ± SD derived from a Gaussian fit. *Data displayed for non-pushed OCCM and ORC in (D) and (J) were combined with data shown in Figure 2D-ATPγS and ATP condition, respectively. See also Figure S5.

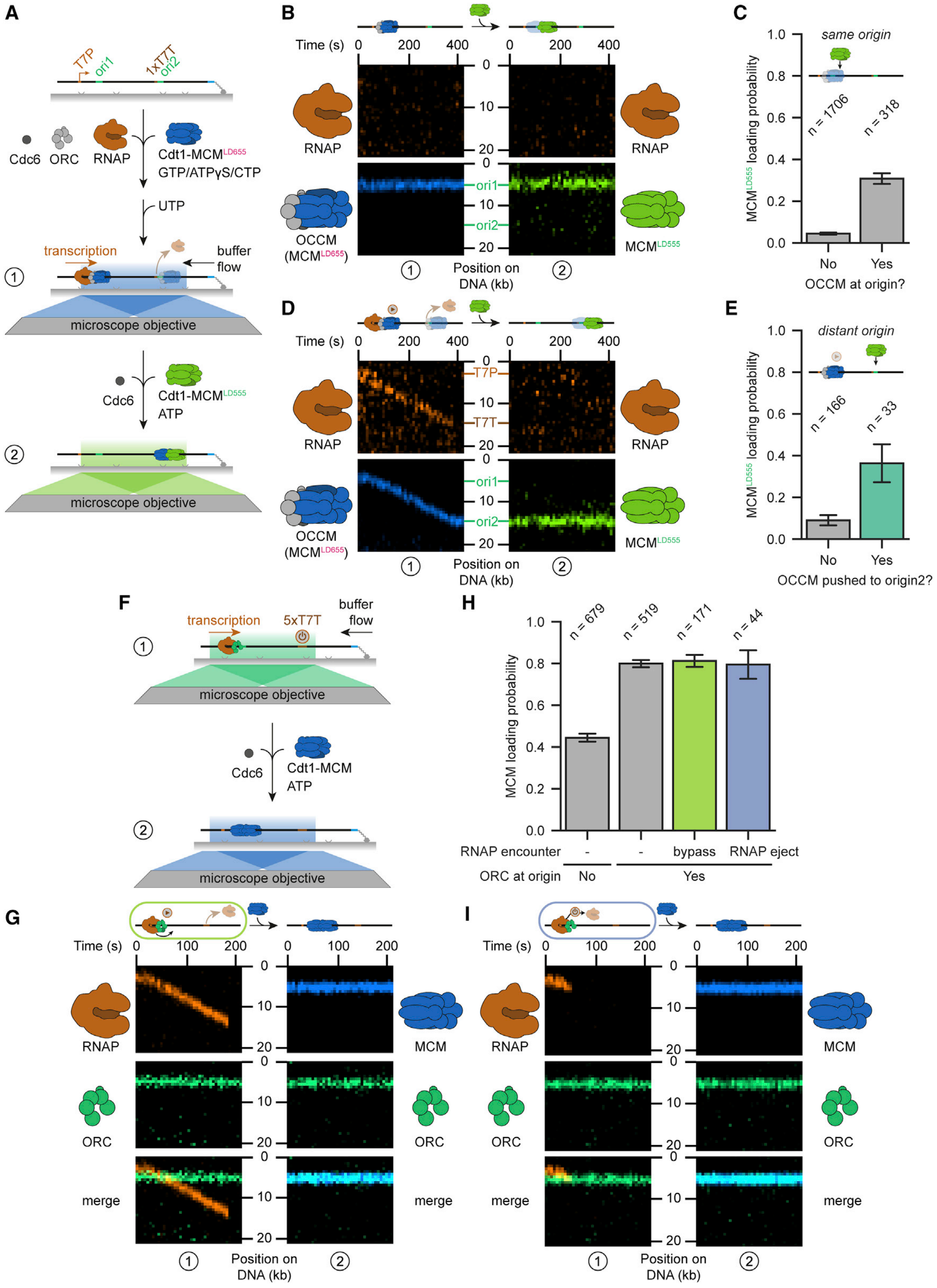


**Figure 6. Origin licensing continues after RNAP encounters**

(A) Schematic of the two-step assay to address OCCM functionality after encounters with RNAP. LD655-OCCM complexes were formed on 21-kb DNA containing one origin (ori1) near the T7 promoter (T7P) and a second origin (ori2) right downstream of a single T7 terminator (T7T). Encounters with RNAP were

(legend continued on next page)

great surprise, the recruitment of new MCMs was much more likely at origins that were occupied by OCCM (Figures 6B and 6C), revealing a mechanism whereby the MCM is lost, but the ORC within the OCCM remains and continues loading. Notably, since ORC was not included in the second incubation step, we did not observe any MCM loading at origins that were cleared of OCCM by RNAP (0/79 molecules), confirming that continued loading depends on the OCCM complex having been at the origin.

We then examined the faction of OCCMs that were relocated from the first origin to the second one and found a more than 4-fold increase in MCM recruitment at the second origin, demonstrating that relocation by RNAP did not disrupt function. Here again, the original MCM helicases that arrived at the second origin were lost, with new rounds of MCM loading dependent on the presence of the ORC, which remained active after arriving at the new location within the pushed OCCM complexes (Figures 6D and 6E).

Our attempts to relocate ORC to the termination site failed to yield a sufficient population for further investigation in a second incubation step, also with the addition of the second origin. We speculate that the presence of flow required to introduce fresh loading factors restricted ORC sampling, inhibiting rebinding to the second origin. Nevertheless, we could fully evaluate the functionality of ORC alone after encounters with RNAP by using bypass and ejection events in which ORC remained (Figure 6F). These experiments revealed that ORC remains equally functional after being bypassed by RNAP or after a collision where RNAP is ejected (Figures 6G–6I). Many unoccupied origins are rebound by ORC molecules during the second incubation with Cdt1-MCM, although no fresh ORC was included in the second incubation step. Nevertheless, MCM loading was highly enhanced if ORC was present at the start of the experiment. The continued functionality of ORC and OCCM after encounters with RNAP demonstrates that the dynamic events we have observed provide true resistance to transcription conflicts by allowing continuation of helicase loading.

## DISCUSSION

To clarify the consequences of dynamic challenges to origin licensing, we reconstituted the process at the single-molecule level with high temporal and spatial resolution. This allowed for direct observations of the departure of licensing factors, changes in the composition of loading intermediates, and tracking of the positions of individual factors at and around replication origins as a function of time. Our findings strongly support a model in which origin-licensing intermediates overcome transcription conflicts by repositioning in front of advancing RNAPs. We observe increased stability as the pathway progresses, with MCM DH being the most stable and ORC the least stable. Nevertheless, we find that RNAP can frequently bypass origin-bound ORC, compensating for its lower sliding stability. Finally, we demonstrate that origin licensing continues after RNAP encounters and relocalization. Taken together, our observations reveal numerous additional pathways for origin specification and resistance to transcription, which are summarized in Figure 7.

Origin-licensing factors conduct helicase loading in diverse and evolving local environments, each with unique challenges. Encounters with polymerases and translocases, known to operate at the same cell cycle stage, pose significant risks. Although some of the mechanisms that ensure genome integrity by overcoming conflicts at the replication fork are becoming clear (Branzei and Foiani, 2010; Sparks et al., 2019), the pathways of resistance at earlier stages of replication have not been well elaborated. Transcription is an acute example of an orthogonal process that can disrupt origin function (Looke et al., 2010; Macheret and Halazonetis, 2018; Tanaka et al., 1994). To reduce the frequency of conflicts, most origins have evolved in locations outside transcribed regions, but some overlap appears unavoidable (Edwards et al., 2002; Harvey and Newport, 2003). Moreover, defects in transcription termination (Gros et al., 2015; Mischo and Proudfoot, 2013), pervasive transcription (Jensen et al., 2013), and heterogeneous mRNA transcription (Pelechano et al., 2013) may lead to collisions between RNAP and origin-licensing factors even outside actively transcribed genes.

Our observations reveal that origin-licensing intermediates are not frequently disassembled during encounters with transcribing RNAPs but instead are mobilized and repositioned. Among the loading intermediates we evaluated, MCM DHs exhibited an extreme robustness consistent with the previously observed replication competence of MCM DHs laterally displaced by RNAP (Gros et al., 2015), the observation of trains of MCMs

visualized in the presence of ATPγS as described in Figure 5A (1). Subsequently, Cdc6 and Cdt1-LD555-MCM were added and incubated in the presence of ATP, and products were visualized (2).

(B) Representative kymograph showing continued origin licensing at origins occupied (ori1) by LD655-OCCM (blue) but not at free origins (ori2) as determined by loading of LD555-MCM (green) to the same origin in the absence of encounters with RNAP (amber).

(C) Quantification of the LD555-MCM loading probability at free origins compared with origins occupied by LD655-OCCM.

(D) Representative kymograph demonstrating continued origin licensing at distant origins (ori2) after LD655-OCCM (blue) was pushed by RNAP (amber) as determined by loading of LD555-MCM (green).

(E) Quantification of the LD555-MCM loading probability at distant origins (ori2) without or with LD655-OCCM being dropped off by RNAP pushing. All analyzed DNA molecules contained LD655-OCCM at ori1 but not at ori2 at the start of the experiment.

(F) Schematic of the two-step assay to address ORC functionality after encounters with RNAP. ORC was loaded on 5×T7T -DNA, and encounters with RNAP were visualized in the presence of ATP as described in Figure 5F (1). Subsequently, Cdc6 and Cdt1-MCM were added and incubated, and products were visualized (2).

(G) Representative kymograph showing continued origin licensing after RNAP (amber) bypassed ORC (green) as determined by loading of MCM (blue).

(H) Quantification of the MCM loading probability in the second incubation step (2) after RNAP encounters with ORC. MCM loading is highly enhanced if ORC was present at the start of the experiment.

(I) Representative kymograph demonstrating that origin licensing continued after RNAP (amber) was ejected upon encountering ORC (green) as determined by loading of MCM (blue). Bar plots in (C), (E), and (H) display the mean and SEM. See also Figures S6 and S7.

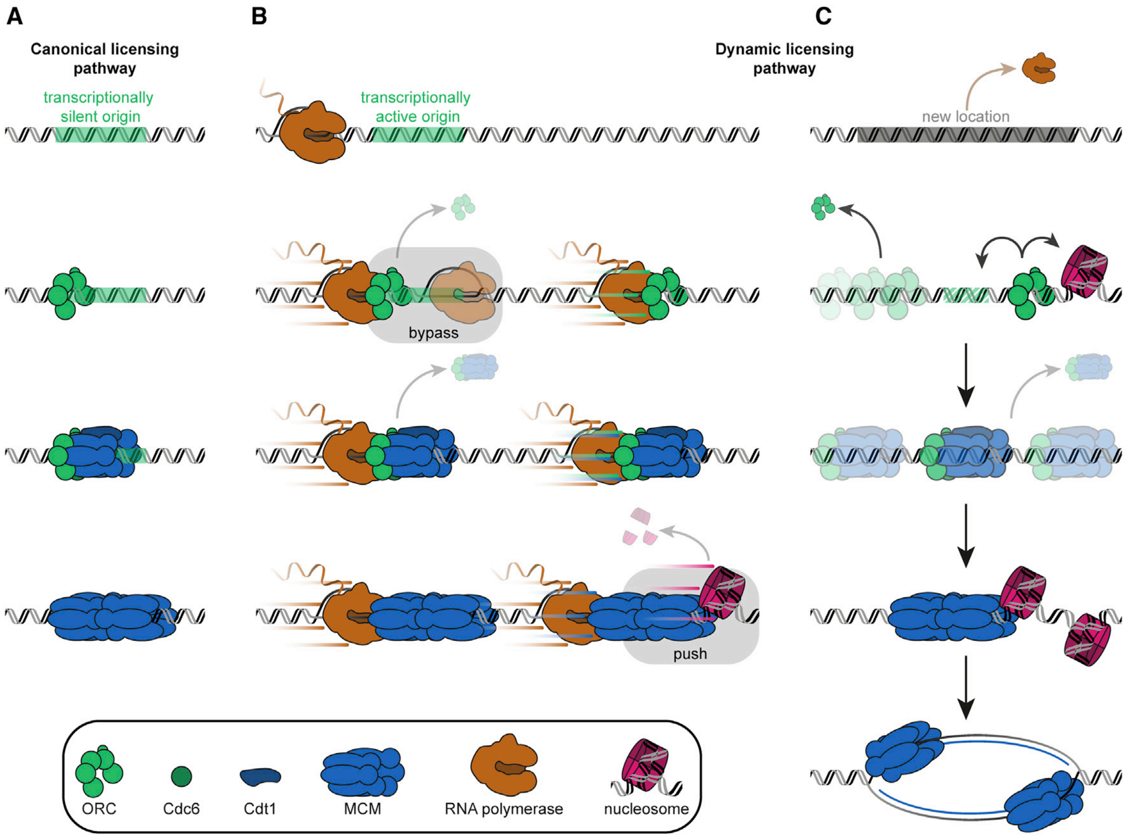


**Figure 7. Mobile origin-licensing factors confer resistance to transcription conflicts**

(A) Canonical origin-licensing pathway at transcriptionally silent origins. The MCM DH is loaded at the origin sequentially via multiple licensing intermediates.

(B) Dynamic origin-licensing pathway at transcriptionally active origins. Origin-licensing intermediates are repositioned by RNAP with increasing stability as the pathway progresses. RNAP can bypass ORC at the origin. Nucleosomes are pushed or ejected during repositioning.

(C) Origin licensing continues at new locations. Although ORC is unstable after relocation by RNAP, an interaction with an additional factor (e.g., a nucleosome) or sequence element could mediate continuation of helicase loading. OCCM remains competent to continue new rounds of MCM loading after transcription terminates. MCM firing takes place at a new location.

visualized by EM (Douglas et al., 2018), the evidence suggesting that MCMs can be pushed by advancing replication forks (Sedlackova et al., 2020), and the observation that an additional helicase is required to offload MCMs (Hill et al., 2020; Schauer et al., 2020). Structural studies have demonstrated that MCM DHs make extensive contacts with both strands of DNA duplex inside the axial channel (Abid Ali et al., 2017; Noguchi et al., 2017), inducing a slight bend in DNA. Our observations of spontaneous MCM DH sliding and minimal reduction in transcription rates even after RNAP collisions with multiple MCM DHs are therefore surprising. On the other hand, this type of DNA engagement likely explains our observation that spontaneous MCM DH sliding is a rare event at physiological salt conditions and may underlie the recent observation that MCMs aid in global genome organization by functioning as roadblocks to loop extrusion by cohesin (Dequeker et al., 2020). Thus, collisions between different protein machineries and MCM DHs are not equivalent and are likely influenced by the force exerted on the MCM DH by a colliding DNA translocase.

Our collision experiments unexpectedly showed that several loading intermediates formed prior to completion of MCM DH formation can be mobilized and repositioned. In particular, the OCCM complex, comprised of ORC, Cdc6, Cdt1, and a single MCM hexamer (Yuan et al., 2017), was readily mobilized and easily repositioned by RNAP. Despite its anticipated lower stability than the MCM DH, a large fraction of OCCMs remained after repositioning to new locations. In contrast, ORC was frequently ejected from DNA during encounters with RNAP, and most repositioned ORC molecules were unstable at new locations. Nevertheless, we observed several dynamic pathways unique to ORC. In particular, on frequent occasions RNAP bypassed ARS1-bound ORC, which was never observed for OCCM or MCM DH. Structural characterization of DNA-bound ORC has revealed extensive sequence-specific as well as large, non-sequence-specific ORC-DNA contact surfaces that likely facilitate ORC sliding (Hu et al., 2020; Jaremko et al., 2020; Li et al., 2018; Schmidt and Bleichert, 2020). For RNAP to pass, ORC would have to partially or fully disengage from the origin. Interestingly, several reports have demonstrated that ORC binds single-stranded DNA (Hoshina et al., 2013; Kawakami et al., 2019; Lee et al., 2000), allowing for the possibility that ORC could remain bound to the excluded single strand of the transcription

bubble and rebind the origin after passage of RNAP. It remains unclear whether the ability to engage DNA in multiple modes is conserved in metazoan ORC, which exhibits a significantly reduced DNA binding surface and greatly reduced DNA binding specificity compared with yeast ORC (Jaremko et al., 2020; Schmidt and Bleichert, 2020).

ORC and OCCM remain functional and continue origin licensing after encounters with RNAP, completing transcription resistance, and opening up alternative pathways of origin specification. After RNAP encounters ORC and is ejected or bypasses, ORC continues origin licensing with no reduction in activity. In contrast, as noted above, ORC is unstable after relocation by RNAP to new locations. This demonstrates that an interaction with an additional factor or sequence element is required for continued helicase loading by ORC at new locations. Consistent with this requirement, we observe continued origin licensing by OCCM after relocation by RNAP that relies on new rounds of MCM loading by the ORC delivered within the OCCM. ORC can also hitch a ride on sliding MCM DHs, opening up yet another possible pathway for stable relocation. Beyond interactions with MCMs, ORC is known to bind nucleosomes (De Ioannes et al., 2019; Kuo et al., 2012; Shen et al., 2010), and it has recently been reported that this interaction allows for origin licensing at non-origin sites (Li et al., 2021). ORC-DNA interactions could likewise serve to stabilize ORC at new locations. Yeast in particular contains many more ACS motifs than functional origins in its genome (Nieduszynski et al., 2006). Finally, more specialized origin-binding factors unique to individual organisms, e.g., Fkh1 (Hoggard et al., 2021), may assist in restarting origin licensing by ORC after relocation by RNAP.

Diffusive sliding has emerged as a vital and intrinsic feature of numerous factors that operate on chromosomes. One explanation for this common property is the benefit conferred when the chromosome is being searched. Our findings support the notion that sliding is equally important once sites have been located and downstream pathways have been activated. Under these circumstances, sliding serves an important additional function providing an intrinsic resistance to disruption, which allows pathways to proceed at new locations. Our choice of T7 RNA polymerase as an orthogonal machinery to challenge origin licensing removes the possibility of any specific contacts playing an important role while exerting forces comparable to eukaryotic RNA polymerase II (Galburt et al., 2007; Thomen et al., 2008). Therefore, our observations provide a broad framework for predicting the dynamic events and outcomes during conflicts with diverse families of polymerases and translocases beyond origin licensing.

Our observation that nucleosomes are frequently pushed during encounters between RNAPs and MCMs reveals a dramatic reshaping of local chromatin architecture at the origin. In higher eukaryotes, where sequence plays only a minor role in defining origins, hallmarks in chromatin likely assist in marking origins. Transplantation of these unique features could lead to ORC recruitment and further rounds of MCM loading at new sites. Moreover, if key chromatin hallmarks of relocated origins could be propagated to new generations, this may lead to the birth of new origins. MCM sliding could also have negative consequences, potentially leading to complete loss or reprogramming of critical gene expression patterns. The presence of MCM ahead of RNAP is likely to disrupt histone inheritance pathways by blocking engagement by the histone processing machinery that travels with the polymerase. Further studies are required to explore these potential outcomes.

We have demonstrated that the mobilization of origin-licensing factors provides resistance pathways to overcome challenges from orthogonal processes on the chromosome. However, this mobilization also provides further avenues for specialized regulation. Interestingly, recent work has uncovered a mechanistic link between transcriptional silencing and late replication wherein histone deacetylation can trigger transcription-mediated displacement of MCMs in rDNA repeats to modulate rDNA origin efficiency (Foss et al., 2019). Pathways like these may be part of larger programs in which mobilization of origin-licensing factors is leveraged to further regulate or augment downstream pathways. Therefore, we anticipate that the dynamics we see are likely to have significant implications for numerous essential pathways beyond replication.

## Limitations of the study

We have shown that origin-licensing intermediates are easily pushed to new locations by RNA polymerase, and remain active to continue helicase loading after these encounters, by reconstituting these events *in vitro*. While our findings suggest that these pathways could result from repositioning of several different origin-licensing intermediates on the pathway to MCM double-hexamer formation, current *in vivo* observations have reported only on the final outcome of these encounters without discriminating between distinct origin licensing intermediates. Furthermore, our use of ATP$\gamma$S to trap OCCM complexes prevents direct loading of a second MCM. Therefore, further studies are needed to clarify whether OCCM complexes relocated to random sites by RNA polymerase remain functional for direct loading of a second MCM hexamer. Although the T7 RNA polymerase used in our study exerts a similar force as RNA polymerase II (Galburt et al., 2007; Thomen et al., 2008), there may be differences in the dynamics observed with the larger polymerase. And finally, chromatin remodelers and chaperones were not included in our study, but are likely to play an important role in the dynamics of repositioning of origin licensing factors.

## STAR★METHODS

Detailed methods are provided in the online version of this paper and include the following:

- KEY RESOURCES TABLE
- RESOURCE AVAILABILITY
  - Lead contact
  - Materials availability
  - Data and code availability
- EXPERIMENTAL MODEL AND SUBJECT DETAILS
  - Yeast strains
  - Bacterial cells
- METHOD DETAILS
  - Protein purification and labeling
  - DNA substrates preparation

- Ensemble pulldown assays
- Single-molecule assays
- Single-molecule data analysis
- QUANTIFICATION AND STATISTICAL ANALYSIS

## SUPPLEMENTAL INFORMATION

Supplemental information can be found online at https://doi.org/10.1016/j.celrep.2022.110531.

## ACKNOWLEDGMENTS

We would like to thank Boris Pfander, Michael Sattler, and Lorenzo Galanti for many helpful comments. We also thank Stephen Bell for providing yeast strain yST163 and antibody SB49 and the Max Planck Institute of Biochemistry Core Facility for producing fluorescent peptides. This work was supported by a starting grant from the European Research Council (Project Number: 804098, REPLISOMEBYPASS) and the Max Planck Society. Work in the Remus lab is supported by NIGMS grants R01-GM127428 and R01GM107239.

## AUTHOR CONTRIBUTIONS

Conceptualization, M.J.S., D.R., and K.E.D; Methodology, M.J.S. and K.E.D.; Software, M.J.S. and K.E.D.; Investigation, M.J.S.; Writing – Original Draft, M.J.S., D.R., and K.E.D.; Writing – Review and Editing, M.J.S., D.R., and K.E.D.; Visualization, M.J.S. and K.E.D.; Resources, M.J.S. and S.A.; Supervision, D.R. and K.E.D.; Funding Acquisition, K.E.D.

## DECLARATION OF INTERESTS

The authors declare no competing interests.

## INCLUSION AND DIVERSITY

One or more of the authors of this paper self-identifies as a member of the LGBTQ+ community.

Received: January 18, 2021
Revised: December 10, 2021
Accepted: February 23, 2022
Published: March 22, 2022

## REFERENCES

Abid Ali, F., Douglas, M.E., Locke, J., Pye, V.E., Nans, A., Diffley, J.F.X., and Costa, A. (2017). Cryo-EM structure of a licensed DNA replication origin. Nat. Commun. *8*, 2241.

Aitken, C.E., Marshall, R.A., and Puglisi, J.D. (2008). An oxygen scavenging system for improvement of dye stability in single-molecule fluorescence experiments. Biophys. J. *94*, 1826–1835.

Azmi, I.F., Watanabe, S., Maloney, M.F., Kang, S., Belsky, J.A., MacAlpine, D.M., Peterson, C.L., and Bell, S.P. (2017). Nucleosomes influence multiple steps during replication initiation. Elife *6*, e22512.

Bleichert, F., Botchan, M.R., and Berger, J.M. (2017). Mechanisms for initiating cellular DNA replication. Science *355*, eaah6317.

Bramhill, D., and Kornberg, A. (1988). Duplex opening by dnaA protein at novel sequences in initiation of replication at the origin of the E. coli chromosome. Cell *52*, 743–755.

Branzei, D., and Foiani, M. (2010). Maintaining genome stability at the replication fork. Nat. Rev. Mol. Cell Biol. *11*, 208–219.

Candelli, T., Gros, J., and Libri, D. (2018). Pervasive transcription fine-tunes replication origin activity. Elife *7*, e40802.

Chen, I., Dorr, B.M., and Liu, D.R. (2011). A general strategy for the evolution of bond-forming enzymes using yeast display. Proc. Natl. Acad. Sci. U S A *108*, 11399–11404.

Chen, J., Zhang, Z., Li, L., Chen, B.C., Revyakin, A., Hajj, B., Legant, W., Dahan, M., Lionnet, T., Betzig, E., et al. (2014). Single-molecule dynamics of enhanceosome assembly in embryonic stem cells. Cell *156*, 1274–1285.

Davidson, I.F., Goetz, D., Zaczek, M.P., Molodtsov, M.I., Huis In 't Veld, P.J., Weissmann, F., Litos, G., Cisneros, D.A., Ocampo-Hafalla, M., Ladurner, R., et al. (2016). Rapid movement and transcriptional re-localization of human cohesin on DNA. EMBO J. *35*, 2671–2685.

De Ioannes, P., Leon, V.A., Kuang, Z., Wang, M., Boeke, J.D., Hochwagen, A., and Armache, K.J. (2019). Structure and function of the Orc1 BAH-nucleosome complex. Nat. Commun. *10*, 2894.

Dequeker, B.J.H., Brandão, H.B., Scherr, M.J., Gassler, J., Powell, S., Gaspar, I., Flyamer, I.M., Tang, W., Stocsits, R., Davidson, I.F., et al. (2020). MCM complexes are barriers that restrict cohesin-mediated loop extrusion. Preprint at bioRxiv. https://doi.org/10.1101/2020.10.15.340356.

Donovan, S., Harwood, J., Drury, L.S., and Diffley, J.F. (1997). Cdc6p-dependent loading of Mcm proteins onto pre-replicative chromatin in budding yeast. Proc. Natl. Acad. Sci. U S A *94*, 5611–5616.

Douglas, M.E., Ali, F.A., Costa, A., and Diffley, J.F.X. (2018). The mechanism of eukaryotic CMG helicase activation. Nature *555*, 265–268.

Duzdevich, D., Warner, M.D., Ticau, S., Ivica, N.A., Bell, S.P., and Greene, E.C. (2015). The dynamics of eukaryotic replication initiation: origin specificity, licensing, and firing at the single-molecule level. Mol. Cell *58*, 483–494.

Edelstein, A., Amodaj, N., Hoover, K., Vale, R., and Stuurman, N. (2010). Computer control of microscopes using microManager. Curr. Protoc. Mol. Biol. Chapter *14*, Unit14 20.

Edwards, M.C., Tutter, A.V., Cvetic, C., Gilbert, C.H., Prokhorova, T.A., and Walter, J.C. (2002). MCM2-7 complexes bind chromatin in a distributed pattern surrounding the origin recognition complex in Xenopus egg extracts. J. Biol. Chem. *277*, 33049–33057.

Evrin, C., Clarke, P., Zech, J., Lurz, R., Sun, J., Uhle, S., Li, H., Stillman, B., and Speck, C. (2009). A double-hexameric MCM2-7 complex is loaded onto origin DNA during licensing of eukaryotic DNA replication. Proc. Natl. Acad. Sci. U S A *106*, 20240–20245.

Foss, E.J., Gatbonton-Schwager, T., Thiesen, A.H., Taylor, E., Soriano, R., Lao, U., MacAlpine, D.M., and Bedalov, A. (2019). Sir2 suppresses transcription-mediated displacement of Mcm2-7 replicative helicases at the ribosomal DNA repeats. PLoS Genet. *15*, e1008138.

Freiburger, L., Sonntag, M., Hennig, J., Li, J., Zou, P., and Sattler, M. (2015). Efficient segmental isotope labeling of multi-domain proteins using Sortase A. J. Biomol. NMR *63*, 1–8.

Frigola, J., Remus, D., Mehanna, A., and Diffley, J.F. (2013). ATPase-dependent quality control of DNA replication origin licensing. Nature *495*, 339–343.

Galburt, E.A., Grill, S.W., Wiedmann, A., Lubkowska, L., Choy, J., Nogales, E., Kashlev, M., and Bustamante, C. (2007). Backtracking determines the force sensitivity of RNAP II in a factor-dependent manner. Nature *446*, 820–823.

Graham, J.E., Marians, K.J., and Kowalczykowski, S.C. (2017). Independent and stochastic action of DNA polymerases in the replisome. Cell *169*, 1201–1213.e17.

Gros, J., Devbhandari, S., and Remus, D. (2014). Origin plasticity during budding yeast DNA replication in vitro. EMBO J. *33*, 621–636.

Gros, J., Kumar, C., Lynch, G., Yadav, T., Whitehouse, I., and Remus, D. (2015). Post-licensing specification of eukaryotic replication origins by facilitated Mcm2-7 sliding along DNA. Mol. Cell *60*, 797–807.

Gruszka, D.T., Xie, S., Kimura, H., and Yardimci, H. (2020). Single-molecule imaging reveals control of parental histone recycling by free histones during DNA replication. Sci. Adv. *6*, eabc0330.

Gupta, S., Friedman, L.J., Gelles, J., and Bell, S.P. (2021). A helicase-tethered ORC flip enables bidirectional helicase loading. Preprint at bioRxiv. https://doi.org/10.7554/eLife.74282.

Harris, C.R., Millman, K.J., van der Walt, S.J., Gommers, R., Virtanen, P., Cournapeau, D., Wieser, E., Taylor, J., Berg, S., Smith, N.J., et al. (2020). Array programming with NumPy. Nature *585*, 357–362.

Harvey, K.J., and Newport, J. (2003). CpG methylation of DNA restricts prereplication complex assembly in Xenopus egg extracts. Mol. Cell Biol. *23*, 6769–6779.

He, B., Rong, M., Lyakhov, D., Gartenstein, H., Diaz, G., Castagna, R., McAllister, W.T., and Durbin, R.K. (1997). Rapid mutagenesis and purification of phage RNA polymerases. Protein Expr. Purif. *9*, 142–151.

Hill, F.R., van Oijen, A.M., and Duderstadt, K.E. (2018). Detection of kinetic change points in piece-wise linear single molecule motion. J. Chem. Phys. *148*, 123317.

Hill, J., Eickhoff, P., Drury, L.S., Costa, A., and Diffley, J.F.X. (2020). The eukaryotic replisome requires an additional helicase to disarm dormant replication origins. Preprint at bioRxiv. https://doi.org/10.1101/2020.09.17.301366.

Hodges, C., Bintu, L., Lubkowska, L., Kashlev, M., and Bustamante, C. (2009). Nucleosomal fluctuations govern the transcription dynamics of RNA polymerase II. Science *325*, 626–628.

Hoggard, T., Hollatz, A.J., Cherney, R.E., Seman, M.R., and Fox, C.A. (2021). The Fkh1 Forkhead associated domain promotes ORC binding to a subset of DNA replication origins in budding yeast. Nucleic Acids Res. *49*, 10207–10220.

Hoshina, S., Yura, K., Teranishi, H., Kiyasu, N., Tominaga, A., Kadoma, H., Nakatsuka, A., Kunichika, T., Obuse, C., and Waga, S. (2013). Human origin recognition complex binds preferentially to G-quadruplex-preferable RNA and single-stranded DNA. J. Biol. Chem. *288*, 30161–30171.

Hu, Y., Tareen, A., Sheu, Y.J., Ireland, W.T., Speck, C., Li, H., Joshua-Tor, L., Kinney, J.B., and Stillman, B. (2020). Evolution of DNA replication origin specification and gene silencing mechanisms. Nat. Commun. *11*, 5175.

Huisjes, N.M., Retzer, T.M., Scherr, M.J., Agarwal, R., Safaric, B., Minnen, A., and Duderstadt, K.E. (2021). Mars, a molecule archive suite for reproducible analysis and reporting of single molecule properties from bioimages. Preprint at bioRxiv. https://doi.org/10.1101/2021.11.26.470105.

Hunter, J.D. (2007). Matplotlib: a 2D graphics environment. Comput. Sci. Eng. *9*, 90–95.

Jacob, F., Brenner, S., and Cuzin, F. (1963). On the regulation of DNA replication in bacteria. Cold Spring Harb. Symp. Quant Biol. *28*, 329–348.

Jaremko, M.J., On, K.F., Thomas, D.R., Stillman, B., and Joshua-Tor, L. (2020). The dynamic nature of the human origin recognition complex revealed through five cryoEM structures. Elife *9*, e58622.

Jensen, T.H., Jacquier, A., and Libri, D. (2013). Dealing with pervasive transcription. Mol. Cell *52*, 473–484.

Kawakami, H., Muraoka, R., Ohashi, E., Kawabata, K., Kanamoto, S., Chichibu, T., Tsurimoto, T., and Katayama, T. (2019). Specific basic patch-dependent multimerization of Saccharomyces cerevisiae ORC on single-stranded DNA promotes ATP hydrolysis. Genes Cells *24*, 608–618.

Knoot, C.J., Purpero, V.M., and Lipscomb, J.D. (2015). Crystal structures of alkylperoxo and anhydride intermediates in an intradiol ring-cleaving dioxygenase. Proc. Natl. Acad. Sci. U S A *112*, 388–393.

Kowalski, D., and Eddy, M.J. (1989). The DNA unwinding element: a novel, cis-acting component that facilitates opening of the Escherichia coli replication origin. EMBO J. *8*, 4335–4344.

Kuo, A.J., Song, J., Cheung, P., Ishibe-Murakami, S., Yamazoe, S., Chen, J.K., Patel, D.J., and Gozani, O. (2012). The BAH domain of ORC1 links H4K20me2 to DNA replication licensing and Meier-Gorlin syndrome. Nature *484*, 115–119.

Larson, J., Kirk, M., Drier, E.A., O'Brien, W., MacKay, J.F., Friedman, L.J., and Hoskins, A.A. (2014). Design and construction of a multiwavelength, micromirror total internal reflectance fluorescence microscope. Nat. Protoc. *9*, 2317–2328.

Lee, D.G., Makhov, A.M., Klemm, R.D., Griffith, J.D., and Bell, S.P. (2000). Regulation of origin recognition complex conformation and ATPase activity: differential effects of single-stranded and double-stranded DNA binding. EMBO J. *19*, 4774–4782.

Lewis, J.S., Spenkelink, L.M., Schauer, G.D., Yurieva, O., Mueller, S.H., Natarajan, V., Kaur, G., Maher, C., Kay, C., O'Donnell, M.E., et al. (2020). Tunability of DNA polymerase stability during eukaryotic DNA replication. Mol. Cell *77*, 17–25. e15.

Li, N., Lam, W.H., Zhai, Y., Cheng, J., Cheng, E., Zhao, Y., Gao, N., and Tye, B.-K. (2018). Structure of the origin recognition complex bound to DNA replication origin. Nature *559*, 217–222.

Li, S., Wasserman, M.R., Yurieva, O., Bai, L., O'Donnell, M.E., and Liu, S. (2021). ORC binds and remodels nucleosomes to specify MCM loading onto DNA. Preprint at bioRxiv. https://doi.org/10.1101/2021.08.17.456647.

Looke, M., Reimand, J., Sedman, T., Sedman, J., Jarvinen, L., Varv, S., Peil, K., Kristjuhan, K., Vilo, J., and Kristjuhan, A. (2010). Relicensing of transcriptionally inactivated replication origins in budding yeast. J. Biol. Chem. *285*, 40004–40011.

Macheret, M., and Halazonetis, T.D. (2018). Intragenic origins due to short G1 phases underlie oncogene-induced DNA replication stress. Nature *555*, 112–116.

Mackiewicz, P., Zakrzewska-Czerwinska, J., Zawilak, A., Dudek, M.R., and Cebrat, S. (2004). Where does bacterial replication start? Rules for predicting the oriC region. Nucleic Acids Res. *32*, 3781–3791.

McKinney, W. (2010). Data structures for statistical computing in Python. Proc. 9th Python Sci. Conf. *445*, 56–61.

Miller, T.C.R., Locke, J., Greiwe, J.F., Diffley, J.F.X., and Costa, A. (2019). Mechanism of head-to-head MCM double-hexamer formation revealed by cryo-EM. Nature *575*, 704–710.

Mirny, L., Slutsky, M., Wunderlich, Z., Tafvizi, A., Leith, J., and Kosmrlj, A. (2009). How a protein searches for its site on DNA: the mechanism of facilitated diffusion. J. Phys. A Math. Theor. *42*, 434013.

Mischo, H.E., and Proudfoot, N.J. (2013). Disengaging polymerase: terminating RNA polymerase II transcription in budding yeast. Biochim. Biophys. Acta *1829*, 174–185.

Nieduszynski, C.A., Knox, Y., and Donaldson, A.D. (2006). Genome-wide identification of replication origins in yeast by comparative genomics. Genes Dev. *20*, 1874–1879.

Noguchi, Y., Yuan, Z., Bai, L., Schneider, S., Zhao, G., Stillman, B., Speck, C., and Li, H. (2017). Cryo-EM structure of Mcm2-7 double hexamer on DNA suggests a lagging-strand DNA extrusion model. Proc. Natl. Acad. Sci. U S A *114*, E9529–E9538.

O'Neill, T.E., Smith, J.G., and Bradbury, E.M. (1993). Histone octamer dissociation is not required for transcript elongation through arrays of nucleosome cores by phage T7 RNA polymerase in vitro. Proc. Natl. Acad. Sci. U S A *90*, 6203–6207.

On, K.F., Beuron, F., Frith, D., Snijders, A.P., Morris, E.P., and Diffley, J.F. (2014). Prereplicative complexes assembled in vitro support origin-dependent and independent DNA replication. EMBO J. *33*, 605–620.

Pelechano, V., Wei, W., and Steinmetz, L.M. (2013). Extensive transcriptional heterogeneity revealed by isoform profiling. Nature *497*, 127–131.

Powell, S.K., MacAlpine, H.K., Prinz, J.A., Li, Y., Belsky, J.A., and MacAlpine, D.M. (2015). Dynamic loading and redistribution of the Mcm2-7 helicase complex through the cell cycle. EMBO J. *34*, 531–543.

Prioleau, M.N., and MacAlpine, D.M. (2016). DNA replication origins-where do we begin? Genes Dev. *30*, 1683–1697.

Rausch, C., Weber, P., Prorok, P., Horl, D., Maiser, A., Lehmkuhl, A., Chagin, V.O., Casas-Delucchi, C.S., Leonhardt, H., and Cardoso, M.C. (2020). Developmental differences in genome replication program and origin activation. Nucleic Acids Res. *48*, 12751–12777.

Remus, D., Beuron, F., Tolun, G., Griffith, J.D., Morris, E.P., and Diffley, J.F. (2009). Concerted loading of Mcm2-7 double hexamers around DNA during DNA replication origin licensing. Cell *139*, 719–730.

Sanchez, H., McCluskey, K., van Laar, T., van Veen, E., Asscher, F.M., Solano, B., Diffley, J.F.X., and Dekker, N.H. (2021). DNA replication origins retain mobile licensing proteins. Nat. Commun. *12*, 1908.

Schauer, G.D., Spenkelink, L.M., Lewis, J.S., Yurieva, O., Mueller, S.H., van Oijen, A.M., and O'Donnell, M.E. (2020). Replisome bypass of a protein-based R-loop block by Pif1. Proc. Natl. Acad. Sci. U S A *117*, 30354–30361.

Scherr, M.J., Safaric, B., and Duderstadt, K.E. (2018). Noise in the machine: alternative pathway sampling is the rule during DNA replication. Bioessays *40*, 1700159.

Schindelin, J., Arganda-Carreras, I., Frise, E., Kaynig, V., Longair, M., Pietzsch, T., Preibisch, S., Rueden, C., Saalfeld, S., Schmid, B., et al. (2012). Fiji: an open-source platform for biological-image analysis. Nat. Methods *9*, 676–682.

Schmidt, J.M., and Bleichert, F. (2020). Structural mechanism for replication origin binding and remodeling by a metazoan origin recognition complex and its co-loader Cdc6. Nat. Commun. *11*, 4263.

Schneider, C.A., Rasband, W.S., and Eliceiri, K.W. (2012). NIH Image to ImageJ: 25 years of image analysis. Nat. Methods *9*, 671–675.

Sedlackova, H., Rask, M.B., Gupta, R., Choudhary, C., Somyajit, K., and Lukas, J. (2020). Equilibrium between nascent and parental MCM proteins protects replicating genomes. Nature *587*, 297–302.

Senavirathne, G., Lopez, M.A., Jr., Messer, R., Fishel, R., and Yoder, K.E. (2018). Expression and purification of nuclease-free protocatechuate 3,4-dioxygenase for prolonged single-molecule fluorescence imaging. Anal. Biochem. *556*, 78–84.

Shen, Z., Sathyan, K.M., Geng, Y., Zheng, R., Chakraborty, A., Freeman, B., Wang, F., Prasanth, K.V., and Prasanth, S.G. (2010). A WD-repeat protein stabilizes ORC binding to chromatin. Mol. Cell *40*, 99–111.

Siddiqui, K., On, K.F., and Diffley, J.F. (2013). Regulating DNA replication in eukarya. Cold Spring Harb. Perspect. Biol. *5*, a012930.

Sparks, J.L., Chistol, G., Gao, A.O., Raschle, M., Larsen, N.B., Mann, M., Duxin, J.P., and Walter, J.C. (2019). The CMG helicase bypasses DNA-protein cross-links to facilitate their repair. Cell *176*, 167–181.e21.

Stigler, J., Camdere, G.O., Koshland, D.E., and Greene, E.C. (2016). Single-molecule imaging reveals a collapsed conformational state for DNA-bound cohesin. Cell Rep. *15*, 988–998.

Studier, F.W. (2005). Protein production by auto-induction in high density shaking cultures. Protein Expr. Purif. *41*, 207–234.

Studitsky, V.M., Clark, D.J., and Felsenfeld, G. (1994). A histone octamer can step around a transcribing polymerase without leaving the template. Cell *76*, 371–382.

Studitsky, V.M., Kassavetis, G.A., Geiduschek, E.P., and Felsenfeld, G. (1997). Mechanism of transcription through the nucleosome by eukaryotic RNA polymerase. Science *278*, 1960–1963.

Tanaka, S., Halter, D., Livingstone-Zatchej, M., Reszel, B., and Thoma, F. (1994). Transcription through the yeast origin of replication ARS1 ends at the ABFI binding site and affects extrachromosomal maintenance of minichromosomes. Nucleic Acids Res. *22*, 3904–3910.

Thomen, P., Lopez, P.J., Bockelmann, U., Guillerez, J., Dreyfus, M., and Heslot, F. (2008). T7 RNA polymerase studied by force measurements varying cofactor concentration. Biophys. J. *95*, 2423–2433.

Ticau, S., Friedman, L.J., Champasa, K., Correa, I.R., Jr., Gelles, J., and Bell, S.P. (2017). Mechanism and timing of Mcm2-7 ring closure during DNA replication origin licensing. Nat. Struct. Mol. Biol. *24*, 309–315.

Ticau, S., Friedman, L.J., Ivica, N.A., Gelles, J., and Bell, S.P. (2015). Single-molecule studies of origin licensing reveal mechanisms ensuring bidirectional helicase loading. Cell *161*, 513–525.

Wang, W., Klein, K.N., Proesmans, K., Yang, H., Marchal, C., Zhu, X., Borrman, T., Hastie, A., Weng, Z., Bechhoefer, J., et al. (2021). Genome-wide mapping of human DNA replication by optical replication mapping supports a stochastic model of eukaryotic replication. Mol. Cell *81*, 2975–2988.e76.

Waskom, M.L. (2021). seaborn: statistical data visualization. J. Open Source Softw. *6*, 3021.

Wasserman, M.R., Schauer, G.D., O'Donnell, M.E., and Liu, S. (2019). Replication fork activation is enabled by a single-stranded DNA gate in CMG helicase. Cell *178*, 600–611.e16.

Worthington, A.S., and Burkart, M.D. (2006). One-pot chemo-enzymatic synthesis of reporter-modified proteins. Org. Biomol. Chem. *4*, 44–46.

Yin, J., Lin, A.J., Golan, D.E., and Walsh, C.T. (2006). Site-specific protein labeling by Sfp phosphopantetheinyl transferase. Nat. Protoc. *1*, 280–285.

Yin, Y.W., and Steitz, T.A. (2002). Structural basis for the transition from initiation to elongation transcription in T7 RNA polymerase. Science *298*, 1387–1395.

Yuan, Z., Riera, A., Bai, L., Sun, J., Nandi, S., Spanos, C., Chen, Z.A., Barbon, M., Rappsilber, J., Stillman, B., et al. (2017). Structural basis of Mcm2-7 replicative helicase loading by ORC-Cdc6 and Cdt1. Nat. Struct. Mol. Biol. *24*, 316–324.

CellPress
OPEN ACCESS

## STAR★METHODS

### KEY RESOURCES TABLE

| REAGENT or RESOURCE | SOURCE | IDENTIFIER |
|---|---|---|
| Antibodies | | |
| Anti-Orc6, mAb, mouse | Stephen Bell Lab (MIT) | SB49 |
| Anti-Cdc6, mAb, mouse | Abcam | Cat# ab20150; RRID: AB_445369 |
| Anti-Mcm4, mAb, mouse | Santa Cruz Biotechnology | Cat# sc-166036; RRID: AB_2012313 |
| THE His Tag Antibody, mAb, mouse | GenScript | Cat# A00186; RRID: AB_914704 |
| Bacterial and virus strains | | |
| *Escherichia coli* DH5α | Thermo Fisher Scientific | Cat# 18265017 |
| *Escherichia coli* BL21(DE3) | Agilent Technologies | Cat# 200131 |
| *Escherichia coli* BL21(DE3) star | Thermo Fisher Scientific | Cat# C601003 |
| *Escherichia coli* BL21(DE3) codon plus RIL | Agilent Technologies | Cat# 230245 |
| Chemicals, peptides, and recombinant proteins | | |
| SNAP-Surface Alexa Fluor 488 | New England Biolabs | Cat# S9129S |
| LD555 maleimide mono-reactive dye | Lumidyne Technologies | Cat# LD555-MAL |
| LD555-CoA | Lumidyne Technologies | Custom synthesis |
| LD655-CoA | Lumidyne Technologies | Custom synthesis |
| LD555-CH(10)LPETGG peptide | This study | N/A |
| SYTOX Orange Nucleic Acid Stain | Thermo Fisher Scientific | Cat# S11368 |
| Trolox | Sigma-Aldrich | Cat# 648471; CAS: 53188-07-1 |
| 3,4-Dihydroxybenzoic acid (PCA) | Sigma-Aldrich | Cat# 03930590; CAS: 99-50-3 |
| Protocatechuate 3,4-Dioxygenase from *Pseudomonas putida* (PCD) | (Senavirathne et al., 2018) | N/A |
| Streptavidin | Sigma-Aldrich | Cat# S4762 |
| Lambda DNA | New England Biolabs | Cat# N3011S |
| Herring sperm DNA | Thermo Fisher Scientific | Cat# 15634017 |
| NTP Bundle | Jena Bioscience | Cat# NU-1014L |
| ATP-γ-S | Sigma-Aldrich | Cat# 11162306001 |
| Poly FLAG Peptide | Bimake | Cat# B23112 |
| Calmodulin Affinity Resin | Agilent Technologies | Cat# 214303 |
| Anti-FLAG M2 Affinity Gel | Sigma-Aldrich | Cat# A2220 |
| Protino Glutathione Agarose 4B | Macherey-Nagel | Cat# 745500.10 |
| CHT Ceramic Hydroxyapatite | Bio-Rad | Cat# 1582200 |
| DYNAL Dynabeads KilobaseBINDER Kit | Thermo Fisher Scientific | Cat# 60101 |
| RNase Inhibitor, Murine | New England Biolabs | Cat# M0314L |
| Exonuclease I | New England Biolabs | Cat# M0293L |
| T4 DNA Ligase | New England Biolabs | Cat# M0202S |
| Restriction endonuclease NotI-HF | New England Biolabs | Cat# R3189S |
| Restriction endonuclease XbaI | New England Biolabs | Cat# R0145S |
| Restriction endonuclease HindIII-HF | New England Biolabs | Cat# R3104S |
| 3-Aminopropyltriethoxysilane (APTES) | Carl Roth | Cat# 2328.1 |
| SYLGARD 184 Silicone Elastomer Kit (Polydimethylsiloxane, PDMS) | VWR | Cat# DOWC634165S |
| Biotin-PEG-Succinimidyl Carbonate (MW 5,000) | Laysan Bio | Cat# Biotin-PEG-SC-5000-1g |
| mPEG-Succinimidyl Carbonate (MW 5,000) | Laysan Bio | Cat# MPEG-SC-5000-5g |
| SFP synthase | (Yin et al., 2006) | N/A |

(*Continued on next page*)

| Continued | | |
|---|---|---|
| REAGENT or RESOURCE | SOURCE | IDENTIFIER |
| Sortase A | (Freiburger et al., 2015) | N/A |
| ORC | (Frigola et al., 2013) | N/A |
| LD555-ORC | (Ticau et al., 2015) & This paper | N/A |
| Cdc6 | (Frigola et al., 2013) | N/A |
| Cdt1-MCM | (Frigola et al., 2013) | N/A |
| Cdt1-LD555/LD655-MCM | This paper | N/A |
| AF488/LD555/LD655-T7 RNAP | This paper | N/A |
| LD555-H3 histone octamers | This paper | N/A |
| Deposited data | | |
| Microscopy data (Part 1):<br>MCM_high-salt_diffusion_part1<br>OCCM_push_MCM_load | This paper | https://doi.org/10.17632/hn34wkg3wg.1 |
| Microscopy data (Part 2):<br>MCM_high-salt_diffusion_part2<br>Dye_lifetimes | This paper | https://doi.org/10.17632/gtz3bzw378.1 |
| Microscopy data (Part 3):<br>Photobleaching_licensed-DNA_ATP<br>Photobleaching_low-chromatin-DNA | This paper | https://doi.org/10.17632/btyb592ykb.1 |
| Microscopy data (Part 4):<br>Photobleaching_licensed-DNA_ATPgS<br>Photobleaching_stalled_RNAP RNAP-ORC-collision_MCM_load | This paper | https://doi.org/10.17632/mjgrth9b27.1 |
| Microscopy data (Part 5):<br>Transcription_naked-DNA_ATP_LD655<br>Transcription_naked-DNA_ATP_LD555<br>Transcription_naked-DNA_ATPgS_LD555<br>Transcription_naked-DNA_ATP_AF488<br>Transcription_naked-DNA_T7T_ATP<br>Transcription_ORC-DNA_ATP<br>Transcription_ORC-DNA_T7T_ATP<br>Transcription_high-chromatin-DNA<br>Transcription_overlicensed-DNA_ATP_LS | This paper | https://doi.org/10.17632/kwvdkvnjw5.1 |
| Microscopy data (Part 6):<br>Transcription_high-chromatin-licensed-DNA_ATP<br>Transcription_licensed-DNA_ATPgS_HS<br>Transcription_licensed-DNA_ATPgS_LS<br>Transcription_licensed-DNA_ATP_HS<br>Transcription_licensed-DNA_ATP_LS<br>Transcription_overlicensed-DNA_ATP_HS | This paper | https://doi.org/10.17632/mbbvr44txv.1 |
| Microscopy data (Part 7):<br>Transcription_low-chromatin-licensed-DNA_ATP<br>Transcription_low-chromatin-DNA | This paper | https://doi.org/10.17632/k5gnp6m64g.1 |
| Microscopy data (Part 8):<br>Transcription_licensed-DNA_T7T_ATP<br>Transcription_licensed-DNA_T7T_ATPgS<br>Transcription_low-chromatin-licensed-DNA_ATPgS | This paper | https://doi.org/10.17632/6jnjzjc482.1 |

(*Continued on next page*)

***Continued***

| REAGENT or RESOURCE | SOURCE | IDENTIFIER |
|---|---|---|
| Experimental models: Organisms/strains | | |
| ySD-ORC (ORC purification) | (Frigola et al., 2013) | N/A |
| yST163 (ORC purification) | (Ticau et al., 2015) | N/A |
| yJF38 (Cdt1-MCM purification) | (Frigola et al., 2013) | N/A |
| ySA4 (Cdt1-MCM purification) | This paper | N/A |
| Oligonucleotides | | |
| See Table S1 | Eurofins | N/A |
| Recombinant DNA | | |
| pSNAP-tag (T7)-2 | New England Biolabs | Cat# N9181S |
| pVP91A-pcaHG (PCD purification) | (Knoot et al., 2015) | Addgene Plasmid #113766 |
| Sfp pet29b C-terminal His Tag (SFP synthase purification) | (Worthington and Burkart, 2006) | Addgene Plasmid #75015 |
| pET29-Sortase 4M (Sortase A purification) | (Freiburger et al., 2015) | N/A |
| pGEX-6P-1_Cdc6 (Cdc6 purification) | (Frigola et al., 2013) | N/A |
| pBH161 | (He et al., 1997) | N/A |
| pMS145_SNAP-T7RNAP (T7 RNAP purification) | This paper | N/A |
| pMS173_ybbR-T7RNAP (T7 RNAP purification) | This paper | N/A |
| pMS184_H2A_H2B (histone octamers purification) | This paper | N/A |
| pMS186_H3-S11C_H4 (histone octamers purification) | This paper | N/A |
| SuperCos1 cosmid vector | Agilent Technologies | Cat# 251301 |
| pMSuperCos162_ARS1 | This paper | N/A |
| pMSuperCos165_T7P-ARS1 | This paper | N/A |
| pMSuperCos177_T7P-ARS1-Widom601 | This paper | N/A |
| pMSuperCos182_T7P-ARS1-Widom601-5xT7T | This paper | N/A |
| pMSuperCos195_T7P-ARS1-Widom601-1xT7T-ARS1 | This paper | N/A |
| Software and algorithms | | |
| Fiji | (Schindelin et al., 2012) | https://imagej.net/Fiji |
| ImageJ | (Schneider et al., 2012) | https://imagej.nih.gov/ij/ |
| Matplotlib v3.2.1 | (Hunter, 2007) | https://doi.org/10.5281/zenodo.3714460 |
| Micromanager v1.4 | (Edelstein et al., 2010) | https://micro-manager.org/ |
| Molecule Archive Suite (Mars) | (Huisjes et al., 2021) | https://github.com/duderstadt-lab |
| NumPy v1.18.5 | (Harris et al., 2020) | https://numpy.org/ |
| Pandas v1.0.4 | (McKinney, 2010) | https://doi.org/10.5281/zenodo.3862857 |
| Python scripts / Jupyter notebooks for analysis | This paper | https://github.com/duderstadt-lab/Born-to-slide |
| Seaborn v0.11.2 | (Waskom, 2021) | https://doi.org/10.5281/zenodo.5205191 |

## RESOURCE AVAILABILITY

### Lead contact

Further information and requests for resources and reagents should be directed to and will be fulfilled by the lead contact, Karl E. Duderstadt (duderstadt@biochem.mpg.de).

### Materials availability

Plasmids and strains generated in this study are available upon request. The only requirement is completion of a Materials Transfer Agreement (MTA).

### Data and code availability

- Raw microscopy data collected in this study have been deposited at Mendeley Data as described in the key resources table.

- Documentation for these tools can be found at https://duderstadt-lab.github.io/mars-docs/. Additional algorithms and scripts used for analysis are available at https://github.com/duderstadt-lab/Born-to-slide.
- Any additional information required to reanalyze the data reported in this paper is available from the lead contact upon request.

## EXPERIMENTAL MODEL AND SUBJECT DETAILS

### Yeast strains

Proteins purified from *Saccharomyces cerevisiae* were expressed in previously described strains ySD-ORC, yST163 and yJF38 as listed in the key resources table except for fluorescently labeled Cdt1-MCM. For this, strain ySA4 (MATa ade2-1 can1-100 pep4::kanMX bar1::hphNAT1 (hygromycinB) ura3::Mcm2-PGal1,10-Mcm3 (URA3) his3-11,15::Gal4-PGal1,10-Cdt1 (HIS3) trp1-1::Mcm5-PGal1,10-Mcm4 (TRP1) leu2-3,112::Mcm7-PGal1, 10-ybbR-Mcm6-FLAG (LEU2, clonNAT)) was generated from yJF38, using linearized plasmids with standard genetic and cloning procedures. Yeast cells were cultured at 30°C in YPD medium or on YPD plates for maintenance. For protein expression, yeast cells were grown in YP medium supplemented with 2% (v/v) raffinose to $OD_{600}$ = 1.2. Subsequently, yeast cells were arrested with α-factor at a final concentration of 150 ng/ml and protein expression was induced by addition of 2% (v/v) galactose.

### Bacterial cells

*Escherichia coli* DH5α ($F^-$ Φ80*lac*ZΔM15 Δ(*lac*ZYA-*arg*F) U169 *rec*A1 *end*A1 *hsd*R17($r_k^-$, $m_k^+$) *pho*A *sup*E44 *thi*- 1 *gyr*A96 *rel*A1 $\lambda^-$) were used for plasmid construction and propagation and were grown in LB medium or on LB plates containing respective antibiotics for selection. For protein expression, *Escherichia coli* BL21 (DE3) or derivatives thereof (BL21 (DE3) star or codon plus RIL) were transformed with plasmids for overexpression and grown in LB, TB or ZYP-5052 auto-induction medium (containing respective antibiotics for selection) to mid log phase at 37°C. Subsequently, the temperature was lowered to 18°C and protein expression was induced by addition of IPTG to a final concentration of 0.1-0.5 mM (except for auto-induction).

## METHOD DETAILS

### Protein purification and labeling

#### *Reagent preparation for protein labeling with SFP synthase*

SFP synthase recognizes a short peptide tag (here the 'ybbR-tag' was used) to which it covalently attaches a CoA-functionalized probe through a phosphopantetheinyl linker, allowing site-specific labeling of proteins. SFP synthase expression and purification was based on a previously described protocol (Yin et al., 2006). *E. coli* BL21(DE3) star (carrying Sfp pet29b C-terminal His Tag, a gift from Michael Burkart (Worthington and Burkart, 2006)) were grown in TB medium at 37°C to OD600 = 1.5 and expression was induced with 0.1 mM IPTG at 18°C for 18 hours. All subsequent purification steps were performed at 4°C. Cells were harvested by centrifugation (4000 x g, 10 min), resuspended in 20 mM Tris-HCl, pH 7.9, 500 mM NaCl, 5 mM imidazole, 1 mM DTT supplemented with 1 x protease inhibitor cocktail (2 μM pepstatin, 2 μM leupeptin, 1 mM PMSF, 1 mM benzamidine, 1 μg/ml aprotinin) and lysed by sonication. The cell lysate was cleared by centrifugation (48000 x g, 30 min) and applied to a HisTrap HP 5 ml (GE Healthcare) equilibrated in 20 mM Tris-HCl, pH 7.9, 500 mM NaCl, 5 mM imidazole, 1 mM DTT. The column was washed with 20 column volumes (CV) of the same buffer, and eluted on a 5-300 mM imidazole gradient in 20 mM Tris-HCl, pH 7.9, 500 mM NaCl, 1 mM DTT. SFP synthase was further purified on a HiLoad 16/600 Superdex 200 pg (GE Healthcare) equilibrated in 50 mM HEPES-NaOH, pH 7.5, 150 mM NaCl, 10 % (v/v) glycerol, 1 mM DTT. Peak fractions were pooled, concentrated with a MWCO 10000 Amicon Ultra Centrifugal Filter unit (Merck), frozen in aliquots in liquid $N_2$ and stored at −80°C.

LD555 and LD655 CoA-functionalized dyes were purchased from Lumidyne Technologies.

#### *Reagent preparation for protein labeling with sortase*

A previously developed, highly efficient version of Sortase A (Δ2-59, P94S/D160N/D165A/K196T) obtained from *Staphylococcus aureus* was used and essentially purified as described elsewhere (Chen et al., 2011; Freiburger et al., 2015). Sortase A was expressed in *E. coli* BL21(DE3) (transformed with pET29-Sortase 4M) in ZYP-5052 auto-induction medium (Studier, 2005) at 18°C. Cleared cell lysate was applied to a HisTrap HP 5 ml equilibrated in 50 mM Tris-HCl, pH 8, 500 mM NaCl, 0.02 % (v/v) $NaN_3$, 10 mM imidazole. Unspecific bound protein and nucleic acids were removed by washing with 50 mM Tris-HCl, pH 8, 0.02 % (v/v) $NaN_3$, 25 mM imidazole + 500 mM (wash 1) or 1000 mM (wash 2) NaCl. Sortase A was then eluted with 250 mM imidazole and dialyzed overnight against 50 mM Tris-HCl, pH 8, 150 mM NaCl, 1 mM DTT in the presence of TEV protease. The reaction mixture was incubated with Ni Sepharose HP beads (GE Healthcare) to recover untagged Sortase A from the supernatant. Sortase A was finally purified via gel filtration chromatography on a HiLoad 26/600 Superdex 75 pg (GE Healthcare), equilibrated with 50 mM Tris-HCl, pH 8, 150 mM NaCl, 0.02 % (v/v) $NaN_3$. Monomeric Sortase A was pooled, concentrated with a MWCO 10000 Amicon Ultra Centrifugal Filter unit, frozen in aliquots in liquid $N_2$ and stored at −80°C.

Generating a fluorescently labeled peptide to site-specifically label proteins at the N-terminus using Sortase A was based on a previously described protocol (Ticau et al., 2015) and further adapted. First, the peptide with the sequence H2N-CH(10)LPETGG-COOH was synthesized by solid-phase peptide synthesis using monomethoxytrityl-protected cysteine (Fmoc-Cys(Mmt)-OH) to prevent

oxidation. Second, the peptide was conjugated with a maleimide mono-reactive dye. For this, 3000 nmols crude product from peptide synthesis were deprotected, mixed with 1000 nmols LD555 maleimide mono-reactive dye in DMSO at pH 7 and incubated at room temperature overnight. Fluorescently labeled, full-length peptide was purified by HPLC on a Gemini 5 μm C6-Phenyl 110 Å LC column using a gradient between 0.1 % (v/v) TFA in $H_2O$ and 0.08 % (v/v) TFA in acetonitrile. Clean fractions containing full-length, labeled peptide (analyzed by mass spectrometry) were pooled, lyophilized and stored at −20°C.

#### ORC and LD555-ORC purification

*S. cerevisiae* ORC expression and purification was based on previously described protocols (Frigola et al., 2013; Ticau et al., 2015). Unlabeled ORC was expressed in yeast strain ySD-ORC (CBP tag at the N-terminus of Orc1). To produce fluorescently labeled ORC, UbSORT-FLAG-ORC (tags at the N-terminus of Orc1) was expressed in yST163. Note that the N-terminal ubiquitin is cleaved off in the cells, exposing three N-terminal glycines on Orc1 for Sortase A recognition (SORT-ORC). Cells were grown in 8 liters YP supplemented with 2 % (v/v) raffinose at 30°C. At $OD_{600}$ = 1.2, cells were arrested at G1 with α-factor at a final concentration of 150 ng/ml and incubated for another 3 hours. Subsequently, protein expression was induced by addition of 2% (v/v) galactose and incubated for another 4 hours. Cells were harvested by centrifugation (4000 x g, 10 min), washed once with 25 mM HEPES-KOH, pH 7.6, 1 M sorbitol and resuspended in 0.5 packed cell volumes of buffer A (25 mM HEPES-KOH, pH 7.6, 0.05 % (v/v) Nonidet P40 Substitute, 10 % (v/v) glycerol, 1 mM DTT) + 500 mM KCl supplemented with 1 x protease inhibitor cocktail and frozen dropwise in liquid $N_2$. Frozen cells were lysed in a SamplePrep Freezer/Mill (SPEX) and subsequently mixed with 0.5 packed cell volumes buffer A + 500 mM KCl supplemented with 1 x protease inhibitor cocktail. All subsequent steps were performed at 4°C unless stated otherwise. Thawed cell lysate was cleared by ultracentrifugation (235000 x g, 60 min).

Cleared cell lysate from ySD-ORC cells was supplemented with 2 mM $CaCl_2$ and incubated with 1.5 ml bed volumes (BV) calmodulin affinity resin equilibrated in buffer A + 500 mM KCl for 3 hours. The resin was washed with 20 BV buffer A + 200 mM KCl, 2 mM $CaCl_2$ and ORC was eluted 10 times with 1 BV of buffer A + 200 mM KCl, 2 mM EGTA, 1 mM EDTA. Fractions were pooled and applied to a HiTrap SP HP 1 ml (GE Healthcare), equilibrated in buffer B (50 mM HEPES-KOH, pH 7.6, 5 mM $Mg(OAc)_2$, 0.01 % (v/v) Nonidet P40 Substitute, 10 % (v/v) glycerol, 1 mM DTT) + 200 mM KCl. The column was washed with 10 CV buffer B + 200 mM KCl and ORC was subsequently eluted with buffer B + 500 mM KCl. Peak fractions were pooled and further purified on a Superdex 200 increase 10/300 gel filtration column (GE Healthcare) equilibrated in buffer B + 300 mM potassium glutamate (KGlu). Fractions containing stoichiometric ORC were pooled, concentrated with MWCO 50000 Amicon Ultra Centrifugal Filter unit, frozen in aliquots in liquid $N_2$ and stored at −80°C.

Cleared cell lysate from yST163 cells was incubated with 2 ml BV Anti-FLAG M2 Affinity Gel equilibrated with buffer A + 500 mM KCl for 3 hours. The resin was washed with 20 BV buffer A + 200 mM KCl and SORT-ORC was eluted 5 times with 1 BV buffer A + 200 mM KCl + 0.15 mg/ml poly FLAG peptide. Elution fractions were pooled and further purified on a HiTrap SP HP 1 ml as described above. To produce LD555-ORC, SORT-ORC was diluted 2-fold with buffer B + 10 mM $CaCl_2$, incubated with Sortase A and LD555-CH(10) LPETGG peptide at a 1:1.3:25 molar ratio for 8 min at 25°C. The reaction was terminated with 20 mM EDTA and purified on a Superdex 200 increase 10/300 gel filtration column equilibrated in buffer B + 300 mM KGlu, 10 mM imidazole. To remove unlabeled SORT-ORC, peak fractions were pooled and applied to a HiTrap Chelating HP 1 ml (GE Healthcare) charged with $Co^{2+}$ equilibrated in buffer B + 300 mM KGlu, 10 mM imidazole. The column was washed with 10 CV buffer B + 300 mM KGlu, 10 mM imidazole and LD555-ORC was eluted with 10 CV buffer B + 300 mM KGlu, 150 mM imidazole. Peak fractions were pooled, concentrated with MWCO 50000 Amicon Ultra Centrifugal Filter unit, frozen in aliquots in liquid $N_2$ and stored at −80°C. The labeling efficiency of LD555-ORC was ∼100% based on extinction coefficients of ORC and LD555.

#### Cdc6 purification

*S. cerevisiae* Cdc6 was purified similar as previously described (Frigola et al., 2013). *E. coli* BL21(DE) codon plus RIL were transformed with pGEX-6P-1_Cdc6, grown in LB to $OD_{600}$ = 0.6 at 37°C and subsequently induced with 0.5 mM IPTG for 5 hours at 18°C. All subsequent steps were performed at 4°C. Cells were harvested by centrifugation (4000 x g, 10 min), resuspended in buffer C (50 mM $K_2HPO_4/KH_2PO_4$, pH 7.5, 5 mM $MgCl_2$, 0.02% (v/v) Tween20, 1 mM DTT) + 150 mM KOAc, 2 mM ATP supplemented with 1 x protease inhibitor cocktail and lysed by sonication. The cell lysate was cleared by centrifugation (48000 x g, 30 min) and the supernatant was incubated with 2 ml BV Protino glutathione agarose 4B for 2 hours. Beads were washed with 20 BV buffer C + 150 mM KOAc, 2 mM ATP and finally diluted to a 50 % slurry with 1 BV of the same buffer. 150 U GST-tagged 3C protease were added and the mixture was incubated overnight. Cleaved Cdc6 was recovered from the flow-through. The final concentration of KOAc was then adjusted to 75 mM by diluting with buffer C + 2 mM ATP and incubated with 2 ml BV CHT ceramic hydroxyapatite resin equilibrated in buffer C + 75 mM KOAc, 2 mM ATP for 2 h. Subsequently, the resin was washed with 5 BV buffer C + 75 mM KOAc and 5 BV buffer C + 150 mM KOAc, 15 % (v/v) glycerol. Cdc6 was eluted 4 times with 1 BV of buffer C + 400 mM KOAc, 15 % (v/v) glycerol. Elution fractions were pooled, concentrated with MWCO 10000 Amicon Ultra Centrifugal Filter unit, frozen in aliquots in liquid $N_2$ and stored at −80°C.

#### Cdt1-MCM and Cdt1-LD555/LD655-MCM purification

Unlabeled *S. cerevisiae* Cdt1-MCM was expressed using yeast strain yJF38 (Frigola et al., 2013). To generate fluorescently labeled Cdt1-MCM, yeast strain ySA4 was generated. Strain ySA4 was modified from yJF38 to overexpress Cdt1 and Mcm2-7 subunits with a ybbR and 3xFLAG tag fused to the N- and C-terminus of Mcm6, respectively. Cells were grown in 6 liters YP supplemented with 2 % (v/v) raffinose at 30°C and arrested at G1 with α-factor at a final concentration of 150 ng/ml at $OD_{600}$ = 1.2. After 3 hours, protein expression was induced by addition of 2 % (v/v) galactose and incubated for another 4 hours. Cells were harvested by centrifugation (4000 x g, 10 min), washed once with cold 0.3 mM PMSF in $ddH_2O$, once with buffer D (100 mM HEPES-KOH, pH 7.6, 0.8 M Sorbitol,

10 mM $Mg(OAc)_2$, 750 mM KGlu) and finally resuspended in 1 packed cell volume of buffer D + 1 mM DTT supplemented with 1 x protease inhibitor cocktail and frozen dropwise in liquid $N_2$. Frozen cells were lysed in a SamplePrep Freezer/Mill and subsequently mixed with 1 packed cell volume buffer E (45 mM HEPES-KOH, pH 7.6, 0.02 % (v/v) Nonidet P40 Substitute, 5 mM $Mg(OAc)_2$, 10 % (v/v) glycerol, 1 mM ATP, 1 mM DTT) + 300 mM KGlu supplemented with 1 x protease inhibitor cocktail. All subsequent steps were performed at 4°C unless stated otherwise. Thawed cell lysate was cleared by ultracentrifugation (235000 x g, 60 min) and incubated with 0.5 ml BV Anti-FLAG M2 Affinity Gel equilibrated with buffer E + 300 mM KGlu for 3 hours. To remove nonspecifically bound protein, the resin was washed twice with 20 BV buffer E + 300 mM KGlu and twice with 20 BV buffer E + 100 mM KGlu. Cdt1-MCM was eluted 5 times with 1 BV buffer E + 100 mM KGlu, 0.5 mg/ml poly FLAG peptide. To produce Cdt1-LD555- or LD655-MCM, following FLAG elution, ybbR-tagged Cdt1-MCM was supplemented with 10 mM $MgCl_2$ and incubated with SFP synthase and LD555- or LD655-CoA at a 1:3:6 molar ratio for 2 hours at 30°C. Unlabeled or LD555/LD655-labeled Cdt1-MCM was further purified on a Superdex 200 increase 10/300 gel filtration column equilibrated in buffer E + 100 mM KOAc. Fractions containing stoichiometric Cdt1-MCM complex were pooled, concentrated with MWCO 50000 Amicon Ultra Centrifugal Filter unit, frozen in aliquots in liquid $N_2$ and stored at −80°C. Labeling efficiencies were estimated to be ∼90 % and 92 % for Cdt1-LD555- and LD655-MCM, respectively based on extinction coefficients of Cdt1-MCM and dyes.

#### AF488-, LD555- and LD655-T7 RNA polymerase purification

To site-specifically label T7 RNA polymerase (RNAP) at its N-terminus, plasmid pBH161 (He et al., 1997) was cut with NcoI and XhoI and the leader sequence was replaced with either SNAP- or ybbR-tag sequence. $His_6$-tagged SNAP26b sequence was amplified from pSNAP-tag (T7)-2 with oligonucleotides MS_144 and MS_145 (pMS145_SNAP-T7RNAP). The ybbR-tag followed by a $His_6$-tag and a GS-linker was added with annealed oligonucleotides MS_221 and MS_222 (pMS173_ybbR-T7RNAP).

*E. coli* BL21(DE3) star were transformed with pMS145_SNAP-T7RNAP or pMS173_ybbR-T7RNAP and grown in TB medium at 37°C to $OD_{600}$ = 1.5. The temperature was lowered to 18°C and T7 RNAP expression induced with 0.1 mM IPTG for 16 hours. All subsequent purification steps were performed at 4°C. Cells were harvested by centrifugation (4000 x g, 10 min), resuspended in buffer F (20 mM $K_2HPO_4/KH_2PO_4$, pH 8, 10 % (v/v) glycerol, 1 mM DTT) + 300 mM KCl, 10 mM imidazole supplemented with 1 x protease inhibitor cocktail and lysed by sonication. The cell lysate was cleared by centrifugation (48000 x g, 30 min) and applied to a HisTrap HP 5 ml equilibrated in buffer F + 300 mM KCl, 10 mM imidazole. After sample application, the column was washed with 20 CV buffer F + 300 mM KCl, 10 mM imidazole, 5 CV buffer F + 1000 mM KCl and 10 CV buffer F + 50 mM KCl, 20 mM imidazole. T7 RNAP was eluted on a 20-300 mM imidazole gradient in buffer F + 50 mM KCl. Peak fractions were pooled and applied to two HiTrap Heparin HP 5 ml (GE Healthcare), equilibrated in buffer F + 50 mM KCl. The columns were washed with 10 CV buffer F + 50 mM KCl and protein was eluted on a 50–1000 mM KCl gradient in buffer F. Peak fractions were pooled, concentrated with a MWCO 50000 Amicon Ultra Centrifugal Filter unit (Merck) and applied to a HiLoad 16/600 Superdex 200 pg (GE Healthcare) equilibrated in buffer G (25 mM HEPES-KOH, pH 8, 150 mM KCl, 10 % (v/v) glycerol, 5 mM DTT). Peak fractions were pooled, spin concentrated and directly used for labeling.

To produce AF488-T7 RNAP, SNAP-T7 RNAP was labeled with a 5-fold molar excess of SNAP-Surface Alexa Fluor 488 in buffer G at 30°C for 2 hours. To produce LD555- and LD655-T7 RNAP, ybbR-T7 RNAP was mixed with SFP synthase and LD555-CoA or LD655-CoA at a 1:2:5 molar ratio, respectively, in buffer G + 10 mM $MgCl_2$ and incubated at 30°C for 2 hours. Labeled T7 RNAP was further purified on a Superdex 200 increase 10/300 gel filtration column equilibrated in buffer G. Peak fractions were pooled, dialyzed against 25 mM HEPES-KOH, pH 8, 150 mM KCl, 50 % (v/v) glycerol, 0.05 % (v/v) Tween20, 1 mM EDTA, 10 mM DTT and stored at −20°C. Labeling efficiencies were estimated to be ∼94 %, 88 % and 87 % for AF488-, LD555-, and LD655-T7 RNAP, respectively based on extinction coefficients of T7 RNAP and dyes.

#### LD555-H3 histone octamers purification

Synthetic genes coding for *S. cerevisiae* histones H2A/H2B and H3/H4 (Eurofins) were cloned into pETDuet-1 (pMS184_H2A_H2B) and pCDFDuet-1 (both Novagen), respectively. Site-specific labeling at H3 was achieved by changing serine11 to cysteine by site-directed mutagenesis in pCDFDuet_H3_H4 (pMS186_H3-S11C_H4). *E. coli* BL21(DE3) codon plus RIL were co-transformed with pMS184_H2A_H2B and pMS186_H3-S11C_H4 and grown in ZYP-5052 auto-induction medium at 37°C to $OD_{600}$ = 0.8. The temperature was lowered to 18°C and growth continued for another 18 hours. All subsequent purification steps were performed at 4°C. Cells were harvested by centrifugation (4000 x g, 10 min), resuspended in buffer H (20 mM HEPES-NaOH, pH 7.6, 10 % (v/v) glycerol, 1 mM EDTA) + 800 mM NaCl, 2 mM DTT, supplemented with 1 x protease inhibitor cocktail and lysed by sonication. The cell lysate was cleared by centrifugation (48000 x g, 30 min) and applied to two HiTrap Heparin HP 5 ml equilibrated in buffer H + 800 mM NaCl, 2 mM DTT. The columns were washed with 15 CV buffer H + 800 mM NaCl, 2 mM DTT and histone octamers were eluted on an 800–2000 mM NaCl gradient in buffer H + 2 mM DTT. Peak fractions were pooled, spin concentrated with a MWCO 10000 Amicon Ultra Centrifugal Filter unit and applied to a HiLoad 16/600 Superdex 200 pg equilibrated in buffer H + 2000 mM NaCl, 2 mM DTT. Peak fractions containing histone octamers were pooled and spin concentrated. Concentrated histone octamers were treated with 10 mM TCEP for 2 hours and DTT was removed with a HiTrap Desalting 5 ml (GE Healthcare) equilibrated in buffer H + 2000 mM NaCl. Subsequently, histone octamers were immediately mixed with a 50-fold molar excess of LD555 maleimide mono-reactive dye. After 20 hours, the reaction was quenched with 10 mM DTT and labeled histone octamers were purified on a Superdex 200 increase 10/300 gel filtration column equilibrated in buffer H + 2000 mM NaCl, 1 mM DTT. Peak fractions were pooled, spin concentrated, frozen in aliquots in liquid $N_2$ and stored at −80°C. Labeling efficiency was estimated to be ∼1.5 dye molecules per histone octamer based on extinction coefficients.

### *Protocatechuate 3,4-Dioxygenase purification*

Single-molecule fluorescence experiments rely on continuous emission of photons from a single fluorophore. The maximum observation time is mainly defined by the total amount of emitted photons which is limited by photobleaching. Since dissolved oxygen is a main reason for photobleaching, enzymatic oxygen scavenging systems have been shown to significantly improve dye lifetime during single-molecule experiments (Aitken et al., 2008). One described enzymatic oxygen scavenging system relies on Protocatechuate 3,4-Dioxygenase (PCD) catalyzed oxidation of 3,4-Dihydroxybenzoic acid (PCA) under oxygen consumption. PCD from *Pseudomonas putida* was basically expressed and purified as previously described (Senavirathne et al., 2018). *E. coli* BL21(DE3) carrying pVP91A-pcaHG (a gift from John Lipscomb (Knoot et al., 2015)) were grown in ZYP-5052 auto-induction medium containing 10 mg/L Fe(II)$SO_4$ at 18°C. Cleared cell lysate was applied to a HisTrap HP 5 ml equilibrated in 50 mM $Na_2HPO_4$/$NaH_2PO_4$, pH 8, 500 mM NaCl, 10 % (v/v) glycerol, 10 mM imidazole, washed with 20 mM imidazole and PCD was eluted with 120 mM imidazole. PCD containing fractions were pooled and further purified via gel filtration chromatography on a HiLoad 26/600 Superdex 200 pg (GE Healthcare), equilibrated with 50 mM Tris-HCl, pH 7.5, 100 mM NaCl, 10 % (v/v) glycerol, 0.1 mM EDTA. Peak fractions were pooled, concentrated to ~20 mg/ml with a MWCO 10000 Amicon Ultra Centrifugal Filter unit, frozen in aliquots in liquid $N_2$ and stored at −80°C. PCD activity (in U/ml) was determined by PCA conversion measured by decreasing absorption at 290 nm.

## DNA substrates preparation

### *pMSuperCos plasmids construction*

The SuperCos1 cosmid vector was used as template to construct all pMSuperCos plasmids. In brief, both cos recognition sequences were removed and replaced by a multiple cloning site of unique restriction enzymes (XbaI-XhoI-SpeI-AsiSI-NheI-FseI-NotI). Subsequently, sequences from λ phage were amplified by PCR and cloned into the plasmid as follows: 177-3007 between XhoI and SpeI, 3008-10142 between AsiSI and NheI, 10143-21084 between NheI and FseI. The resulting plasmid was 27 kb in length containing ~21 kb λ phage sequence. To construct pMSuperCos162_ARS1, yeast origin ARS1 was cloned into BamHI site at position 5506 in the phage sequence. This plasmid was further modified by inserting the T7 phi10 promoter sequence with the first thymidine occurring at position +16 between SpeI and AsiSI site using annealed oligonucleotides MS_180 and MS_181 (pMSuperCos165_T7P-ARS1). A single Widom601 nucleosome positioning sequence was then inserted at NheI site (pMSuperCos177_T7P-ARS1-Widom601). Five tandem T7 terminator sequences (pMSuperCos182_T7P-ARS1-Widom601-5xT7T) or a single T7 terminator sequence immediately followed by a shortened version of ARS1 starting with the ACS right downstream of the terminating base (pMSuperCos195_T7P-ARS1-Widom601-1xT7T-ARS1) were inserted at EcoNI site at position 13514 in the phage sequence.

### *Biotinylated linear DNA preparation for ensemble assays*

For ensemble helicase / RNAP loading and collision assays, biotinylated 5 kb DNA substrates were prepared by PCR using oligonucleotides MS_226 and MS_227 with pMSuperCos162_ARS1 (5kb-ARS1 DNA) or pMSuperCos165_T7P-ARS1 (5kb-T7P-ARS1 DNA) as template. The PCR product was purified on an agarose gel using QIAquick Gel Extraction Kit (Qiagen) and stored in aliquots at −20°C.

### *Biotinylated linear DNA preparation for single-molecule assays*

For single-molecule TIRF assays all DNA substrates were restriction digest fragments (21-22 kb in length) of different pMSuperCos plasmids as indicated (also see key resources table). Plasmids were isolated from *E. coli* DH5α using a Plasmid Maxi Kit (Qiagen). 100 μg plasmid were digested with 100 U XbaI, NotI-HF and HindIII-HF in 1 x CutSmart buffer for 7 hours at 37°C. The resulting XbaI-NotI fragment was separated from the plasmid backbone on a 10-40 % (w/v) sucrose gradient in 20 mM Tris-HCl, pH 7.5, 1 M NaCl, 5 mM EDTA using a SW41 Ti rotor (Beckman Coulter) at 30000 rpm, 20°C for 22 h. Fractions containing pure XbaI-NotI fragment were precipitated with ethanol at −20°C and DNA was reconstituted in 10 mM Tris-HCl, pH 8, 0.1 mM EDTA. DNA handles were prepared by annealing equimolar amounts of oligonucleotides MS_200 with MS_201 and MS_202 with MS_203 in 30 mM HEPES-KOH, pH 7.5, 100 mM KOAc, heating to 95°C for 5 min and cooling to 4°C at −1°C/min. Annealed DNA handles were mixed with the purified XbaI-NotI fragment at a molar ratio of 15:1 and ligated with T4 DNA Ligase in 1 x T4 DNA Ligase buffer at 16°C overnight. Excess DNA handles were removed on a Sephacryl S-1000 SF Tricorn 10/300 gel filtration column (GE Healthcare) equilibrated in 10 mM Tris-HCl, pH 8, 300 mM NaCl, 1 mM EDTA. Peak fractions were pooled, precipitated with ethanol and reconstituted in 10 mM Tris-HCl, pH 8, 1 mM EDTA. Small aliquots of the final DNA substrates were snap frozen in liquid $N_2$ and stored at −80°C. All final DNA substrates for single-molecule assays are functionalized with biotin at NotI site and an 18 bp ssDNA overhang at XbaI site which was used for orientation specific doubly-tethering where indicated.

### *Biotinylated, chromatinized linear DNA preparation for single-molecule assays*

Biotinylated, linear DNA was prepared from pMSuperCos177_T7P-ARS1-Widowm601 as described above. 0.5 μg DNA were incubated with a 50- (“low density”) or 75-fold (“high density”) molar excess of LD555-H3 histone octamers in a total volume of 20 μl buffer H + 2000 mM NaCl, 1 mM DTT on ice for 2 hours. Subsequently, nucleosomes were reconstituted by salt gradient dialysis as follows: The reaction mix was transferred into Slide-A-Lyzer MINI Dialysis Unit, 3.5K MWCO (Thermo Fisher Scientific) and put in a beaker containing 0.5 l buffer H + 2000 mM NaCl, 1 mM DTT. Using a continuous pump flow system at 3 ml/min, buffer H + 50 mM NaCl, 1 mM DTT was continuously added to the dialysis beaker, mixed and removed for 20 hours. Finally, the reaction mix was dialyzed against fresh buffer H + 50 mM NaCl, 1 mM DTT for another 2 hours. Biotinylated, chromatinized linear DNA was stored at 4°C.

## Ensemble pulldown assays

### *Ensemble helicase and RNA polymerase loading assay*

For each reaction, 0.2 pmol biotinylated DNA (5 kb-ARS1 or 5kb-ARS1-T7P DNA) were tethered to 50 μg Dynabeads M-280 Streptavidin using DYNAL Dynabeads KilobaseBINDER Kit according to manufacturer's instructions. For helicase loading, 0.2 pmol tethered DNA were mixed with 1 pmol ORC, 1 pmol Cdc6 and 2 pmol Cdt1-MCM in 20 μl assay buffer (30 mM HEPES-KOH, pH 7.6, 8 mM $Mg(OAc)_2$, 0.05 % (v/v) Tween20, 0.1 mg/ml BSA, 5 mM DTT) + 200 mM KOAc, 3 mM ATP. For RNAP loading, 0.2 pmol tethered DNA were mixed with 2 pmol T7 RNAP in 20 μl assay buffer + 200 mM KOAc, 1 U/μl RNase inhibitor and 0.6 mM of each NTP as indicated. Samples were incubated at 30°C, 1250 rpm for 20 min in a thermoshaker. The supernatant was collected and beads were washed once with 100 μl assay buffer containing either 200 mM KOAc (low salt wash) or 500 mM NaCl (high salt wash) and once with 100 μl assay buffer + 200 mM KOAc. Retained proteins were eluted in 1 x SDS loading buffer at 95°C, 1250 rpm for 5 min. Samples were run on a 4-12% Bis-Tris SDS gel and visualized by Western Blot using protein specific primary antibodies for Orc6, Cdc6, Mcm4 or His Tag to detect T7 RNAP (see key resources table).

### *Ensemble RNA polymerase - helicase collision assay*

Collision assays were set up in two consecutive steps. First, helicase loading was performed on 5kb-T7P-ARS1 DNA as described above (without elution). Second, 0.2 pmol licensed DNA were incubated with 2 pmol T7 RNAP in 20 μl assay buffer + 200 mM KOAc, 1 U/μl RNase inhibitor, 3 mM ATP and 0.6 mM GTP/CTP/UTP as indicated and incubated for another 20 min at 30°C, 1250 rpm in a thermoshaker. The supernatant was collected and beads were washed twice with 100 μl assay buffer + 200 mM KOAc, 1 U/μl RNase inhibitor. Retained proteins were eluted and samples analyzed as described for loading assays.

## Single-molecule assays

### *PEG-biotin microscope slides preparation*

Glass coverslips (22 × 22 mm, Marienfeld) were cleaned with a Zepto plasma cleaner (Diener Electronic) and incubated in acetone containing 2 % (v/v) 3-aminopropyltriethoxysilane for 5 min. Silanized coverslips were rinsed with $ddH_2O$, dried and baked at 110°C for 30 min. Coverslips were then covered with a fresh solution of 0.4 % (w/v) Biotin-PEG-Succinimidyl Carbonate (MW 5,000) and 15 % (w/v) mPEG-Succinimidyl Carbonate (MW 5,000) in fresh 0.1 M $NaHCO_3$ and incubated overnight at room temperature. Coverslips were rinsed with $ddH_2O$, dried and incubated again with in a fresh Biotin-PEG/mPEG solution as described above. Functionalized PEG-Biotin microscope slides were again washed and dried and finally stored under vacuum.

### *Flow cell preparation*

A functionalized PEG-Biotin microscope slide was covered with 0.2 mg/ml streptavidin in blocking buffer (20 mM Tris-HCl, pH 7.5, 50 mM NaCl, 2 mM EDTA, 0.2 mg/ml BSA, 0.005 % (v/v) Tween20) for 30 min. To assemble a flow cell, a polydimethylsiloxane block was placed on top of the previously washed and dried slide, generating a 0.5 mm wide and 0.1 mm high flow cell with a polyethylene tube (inner diameter 0.58 mm) inserted on either end. The assembled flow cell was rinsed with blocking buffer for 5 min. Biotinylated linear DNA was then tethered to the slide surface at 5 pM in blocking buffer for 15 min in absence of buffer flow and the flow cell was subsequently washed with blocking buffer. In case biotinylated, chromatinized linear DNA was used, blocking buffer was supplemented with 0.4 mg/ml herring sperm DNA to remove free histone octamers. In experiments using doubly-tethered DNA, the flow cell was flushed with 100 nM oligonucleotide MS_204 in blocking buffer at 100 μl/min for 12 min. To further reduce non-specific protein binding to the slide surface, the flow cell was washed with assay buffer + 2 mg/ml casein (+ 500 U/ml Exonuclease I for doubly-tethered DNA) and incubated for 40 min.

### *Single-molecule helicase loading assay*

To achieve helicase loading, unless stated otherwise, 0.25 nM (LD555-)ORC, 4 nM Cdc6 and 10 nM Cdt1-LD655-MCM in assay buffer + 200 mM KOAc, 3 mM ATP(γS) were introduced to a prepared flow cell and incubated for 25 min. To favor multiple MCM loading also occurring at non-ARS1 sites, ORC concentration and incubation time were increased to 1 nM and 35 min, respectively. The flow cell was washed with 150 μl assay buffer + 0.6 mM ATP(γS) containing either 200 mM KOAc (low salt wash) or 500 mM NaCl (high salt wash) followed by 300 μl assay buffer + 200 mM KOAc, 0.6 mM ATP(γS) supplemented with an oxygen scavenging system (OSS; consisting of 1 mM Trolox, 2.5 mM PCA, 0.21 U/ml PCD) (Aitken et al., 2008). Subsequently, imaging was started at a constant flow of 50 μl/min. DNA was post stained with 50 nM SYTOX Orange in the same buffer as during imaging.

### *Single-molecule spontaneous helicase sliding assay*

Helicases were loaded as described above using a prepared flow cell with doubly-tethered DNA. Upon helicase loading, the flow cell was washed with 300 μl assay buffer + 500 mM NaCl, 0.6 mM ATP supplemented with an OSS. To eliminate any external force applied by buffer flow, the flow was stopped before imaging (at higher resolution; see imaging conditions) was started. DNA was post stained with 50 nM SYTOX Orange in the same buffer as during imaging.

### *Single-molecule transcription assay*

To visualize RNAP on all DNA molecules in a synchronous manner, transcription assays were set up in two major steps. First, T7 RNAP was loaded on DNA to form stalled elongation complexes. To achieve this, the downstream region of the T7 promoter was designed to not contain dT until position +16. By omitting UTP in the initially supplied NTP mix, T7 RNAP can initiate transcription, switch to its stable elongation mode but is then stalled at position +15 (a distance which should not to support stable loading of a second T7 RNAP) (Yin and Steitz, 2002). Second, after removing unbound T7 RNAP from solution, transcription was continued by supplying all NTPs, which allowed synchronous observation of one transcription event per DNA molecule.

CellPress
OPEN ACCESS

For this, 5 nM labeled T7 RNAP (AF488-, LD555- and LD655-T7 RNAP were used interchangeably) in assay buffer + 200 mM KOAc, 40 U/ml RNase inhibitor, 0.6 mM GTP/CTP/ATP(γS) were introduced to a prepared flow cell and incubated for 25 min. Unbound T7 RNAP washed out with 300 μl assay buffer + 200 mM KOAc, 40 U/ml RNase inhibitor, 0.6 mM GTP/CTP/ATP(γS) supplemented with an OSS. To continue transcription, the flow cell was flushed at 50 μl/min with assay buffer + 200 mM KOAc, 40 U/ml RNase inhibitor, 0.6 mM each NTP (containing ATP or ATPγS) supplemented with an OSS and imaging was started immediately. DNA was post stained with 50 nM SYTOX Orange in the same buffer as during imaging.

#### *Single-molecule RNA polymerase and origin licensing factor collision assay*

Collision assays were set up similar as described for helicase loading and transcription assay. Unless stated otherwise, 0.25 nM (LD555-)ORC, 4 nM Cdc6, 10 nM Cdt1-LD655-MCM and 5 nM labeled T7 RNAP in assay buffer + 200 mM KOAc, 40 U/ml RNase inhibitor, 3 mM ATP(γS), 0.6 mM GTP/CTP were introduced to a prepared flow cell and incubated for 25 min. The flow cell was washed with 150 μl assay buffer + 40 U/ml RNase inhibitor, 0.6 mM GTP/CTP/ATP(γS) containing either 200 mM KOAc (low salt wash) or 500 mM NaCl (high salt wash) followed by 300 μl assay buffer + 200 mM KOAc, 40 U/ml RNase inhibitor, 0.6 mM GTP/CTP/ATP(γS) supplemented with an OSS. To continue transcription, the flow cell was flushed at 50 μl/min with assay buffer + 200 mM KOAc, 40 U/ml RNase inhibitor, 0.6 mM each NTP (containing ATP or ATPγS) supplemented with an OSS and imaging was started immediately. DNA was post stained with 50 nM SYTOX Orange in the same buffer as during imaging.

To test whether ORC and OCCM complexes remain functional and continue origin licensing after encounters with T7 RNAP, LD555-ORC binding (in ATP) and LD655-OCCM formation (in ATPγS) with subsequent transcription were performed as described above. Transcription was imaged at for 3.5 min and 7 min for ORC and OCCM complexes, respectively. Subsequently, 4 nM Cdc6 and 10 nM Cdt1-LD655-MCM (ORC functionality) or Cdt1-LD555-MCM (OCCM functionality) in assay buffer + 200 mM KOAc, 3 mM ATP were immediately introduced to the flow cell and incubated for 15 min. The flow cell was washed with 300 μl assay buffer + 200 mM KOAc 0.6 mM ATP supplemented with an OSS and imaging was continued at 50 μl/min. DNA was post stained as described above.

#### *Assays to determine the stoichiometry of fluorescently labeled proteins*

To determine the stoichiometry of fluorescently labeled proteins, photobleaching experiments were performed. All experiments were essentially performed as described above, except that the frame rate was increased ~10 fold (see imaging conditions) and buffers were not supplemented with OSS to allow photobleaching during the standard imaging time. T7 RNAP bleaching experiments were performed with stalled elongation complexes at the T7 promoter.

#### *Imaging conditions*

Single-molecule assays were performed using an RM21 micromirror TIRF microscope from Mad City Labs (MCL, Madison, Wisconsin, USA) with custom modifications as previously described (Larson et al., 2014) equipped with an Apo N TIRF 60 x oil-immersion TIRF objective (NA 1.49, Olympus) in a temperature-controlled room at 22.5 ± 0.5°C. Alexa Fluor 488, LD555 / SYTOX Orange and LD655 dyes were excited with a 488 nm, 532 nm and 637 nm laser (OBIS 488 nm LS 120 mW, OBIS 532 nm LS 120 mW and OBIS 637 nm LX 100 mW, Coherent), respectively. Residual scattered light from excitation was removed and signals were separated with appropriate emission filter sets (ET520/40 m and ZET532/640 m, Chroma). Emission light was split at 635 nm (T635lpxr, Chroma) and collected on an iXon Ultra 888 EMCCD camera (Andor). All proteins were visualized sequentially every 5-10 s (except for helicase sliding and photobleaching assays performed at 2-4 fps) with a 200 ms integration time for 10-20 min. During imaging, all microscope parts were controlled using Micromanager v1.4 for ImageJ (Edelstein et al., 2010; Schneider et al., 2012) and custom BeanShell scripts.

To determine the lifetimes of the dyes used in this study, single dyes were bound to the surface and excess dye was removed. All dyes were then imaged under identical conditions as describe above. The number of fluorescent dyes was determined for each frame (Molecule Archive Suite peak finder; see raw data processing). To determine the time after which half of single dyes have bleached (b), the number of detected peaks (y) versus frames (x) were fit with $y = a \cdot 0.5^{\frac{x}{b}}$. These experiments revealed that half of the single LD555 and LD655 dyes bleached after ~290 and ~150 frames, respectively (see Figure S1I). Notably, this lifetime is much longer than our observation time to determine collision outcomes and accounts for less than 1.5 % (LD555) or 3 % (LD655) of our observations if single dyes are visualized. Thus, we did not account for photobleaching in our collision analysis. Since AF488 had significantly faster photobleaching kinetics, it was thus not used to determine collision outcomes.

### Single-molecule data analysis

#### *Raw data processing and organization in Molecule Archives*

All single-molecule raw data were processed in Fiji (Schindelin et al., 2012) using Molecule Archive Suite (Mars) commands (Huisjes et al., 2021). First, individual channels were split and corrected for the laser beam profile to lower position-specific differences in fluorescence intensity. Second, to generate Single Molecule Archives, individual molecules were tracked with subpixel resolution with simultaneous integration of fluorescence intensity (inner radius: 2 pixels, outer radius: 4 pixels - for subtracting local background). Stuck molecules on the slide surface in the LD655 channel were used to accurately overlay post-stained DNA videos and to correct all channels for stage drift. Third, all individually separable DNA molecules were fit and checked for colocalization with individual molecule trajectories and finally organized into one combined DNA Molecule Archive. Correct tracking and colocalization with DNA were further evaluated manually.

For transcription experiments, protein position on DNA versus time was fit with a kinetic change-point algorithm (Hill et al., 2018). Individual regions were assigned to distinguish between different pushed proteins and numbers within one transcription trajectory.

#### *Collision outcomes*

Collision outcomes were determined manually by evaluating trajectories and raw videos. Proteins with starting positions closer than ~1 kb were excluded from collision analysis. Collisions were analyzed when proteins approached to < 0.5 kb and classified as follows: Push – displacement > 2 kb; bypass – displacement < 2 kb with transcription > 4 kb; pause – displacement > 2 kb with transient pause within < 2 kb upon collision; stall – displacement < 2 kb with permanent pause; eject – displacement < 2 kb and loss of protein signal. To analyze collisions of two MCM or nucleosome foci, survival of both foci were evaluated by the total fluorescence intensity after collision (which approximately corresponds to the sum of individual intensities prior to collision). Note that 3-color experiments with nucleosome collisions were analyzed independent of AF488 trajectories.

#### *Stability at T7 termination site*

Stability at T7 termination site was determined manually by evaluating trajectories and raw videos. Stability of MCM DHs and origin licensing intermediates was analyzed as follows: Once transcription-driven displacement stopped permanently at the T7 termination site (DNA region 12 – 15 kb considered), trajectories were classified into three groups. (1) Dissociate – protein stayed on DNA for less than 120 s. (2) Remain – protein stayed within less than 2 kb of T7 termination site for more than 120 s. (3) Slide back – protein stayed on DNA for more than 120 s but did not stay within less than 2 kb of T7 termination site for more than 120 s. Note that AF488 was not used to determine collision outcomes due to significantly faster photobleaching kinetics.

#### *Spatial-temporal protein dynamics and kinetics*

Above described Molecule Archives contained all information required for subsequent data analysis. Data was further analyzed directly from Molecule Archives with custom python scripts and Jupyter notebooks.

Protein loading sites were determined by their initial position on DNA. Transcription and pushed distances were calculated by subtracting the protein loading site from the maximum detected position on DNA. Reported transcription rates correspond to burst rates which excluded region of transcription pauses (definition see below). To determine burst transcription rates, first, poorly fitted segments derived from fitting with the kinetic change-point algorithm were excluded (standard deviation > 10 nt/s or rate < -10 nt/s). Second, all remaining segments showing a > 3-fold reduced velocity compared to the mean of non-pause segments were marked as pause segments. Finally, burst transcription rates were calculated from the time-weighted average of non-pause segments. Pausing probability was derived from pauses occurring until position 19 kb on DNA (DNA substrates with termination sites were not analyzed for pausing probability) with segment lengths of > 20 s. Upon pausing, if transcription failed to resume within the observation time, the pause was classified as permanent, otherwise as transient.

Diffusion coefficients (D) were calculated with $D = \frac{<x>^2}{2t}$ where $<x>^2$: mean squared displacement in $kbp^2$ and t: time in s. Population variance ($\sigma^2$) was calculated with $\sigma^2 = \frac{\sum_{i=1}^{N}(x_i - \mu)^2}{N}$ where $x_i$: value of $i^{th}$ element, $\mu$: population mean and N: population size.

#### *Fluorescently labeled protein stoichiometry*

Labeled ORC, MCM, T7 RNAP and nucleosome stoichiometry on DNA was determined by photobleaching experiments as described above. Subsequently, photobleaching steps were fit with kinetic change-point algorithm (Hill et al., 2018). In transcription-helicase collision assays, the number of MCM DHs in an MCM foci was estimated based on their initial fluorescence intensity and the mean fluorescence intensity of one MCM DH obtained from photobleaching analysis. A similar procedure was applied to estimate the number of nucleosomes to detect more than one nucleosome within the resolution limit (mostly in “high density” chromatin).

## QUANTIFICATION AND STATISTICAL ANALYSIS

The number of observations (n) analyzed is indicated in the figure or figure legends. All single-molecule experiments were conducted three or more times independently. Errors reported in this study represent the estimated standard error of the mean (SEM) determined from 10,000 cycles of bootstrapping except for errors of rates which represent the standard deviation (SD) derived from a Gaussian fit as mentioned in the figure legends or text. Python packages NumPy, pandas, matplotlib and seaborn were used for all statistical analysis (Harris et al., 2020; Hunter, 2007; McKinney, 2010; Waskom, 2021). A detailed representation of combined datasets to generate each figure panel is outlined in the supplied Jupyter notebooks.

**Cell Reports, Volume *38***

## Supplemental information

# Mobile origin-licensing factors confer resistance to conflicts with RNA polymerase

**Matthias J. Scherr, Syafiq Abd Wahab, Dirk Remus, and Karl E. Duderstadt**

# Supplemental Information

**Table S1. Sequences of oligonucleotides used in this study, Related to STAR methods.**

| Name | Sequence (5´–3´) |
|---|---|
| MS_144 | GGT TTT CCA TGG GTT CCA GCC ATC ACC ACC ACC ATC ATG GCA GTT CGA TGG ACA AAG ATT GCG AAA TGA AAC G |
| MS_145 | GGT TTT CTC GAG TCC CAG ACC CGG TTT ACC CAG AC |
| MS_180 | [PHO]CTA GTC CCG CGA AAT TAA TAC GAC TCA CTA TAG GGA GAC CAC AAC GGT TTG CGA T |
| MS_181 | [PHO]CGC AAA CCG TTG TGG TCT CCC TAT AGT GAG TCG TAT TAA TTT CGC GGG A |
| MS_200 | [PHO]GGC CGG TGA GTG TGT TTG AGT TGA TTT TGT GTG G |
| MS_201 | [BITEG]CCA CAC AAA ATC AAC TCA AAC ACA CTC ACC |
| MS_202 | [PHO]CTA GGG TGA GTG TGT TTG AGT TGA TTT TGT GTG GGC GTC GGT AAG TGA GAG G |
| MS_203 | CCA CAC AAA ATC AAC TCA AAC ACA CTC ACC |
| MS_204 | [BITEG]CCT CTC ACT TAC CGA CGC |
| MS_221 | [PHO]CAT GGA TAG CCT GGA GTT CAT CGC CTC GAA GTT AGC CGG CAG TTC CCA CCA TCA CCA CCA TCA TGG TGG TGG TGG CTC TC |
| MS_222 | [PHO]TCG AGA GAG CCA CCA CCA CCA TGA TGG TGG TGA TGG TGG GAA CTG CCG GCT AAC TTC GAG GCG ATG AAC TCC AGG CTA TC |
| MS_226 | [BITEG]GCT GCT GCG TGT GGA TGA G |
| MS_227 | CCG CGT GCC TGA GTG TTC |

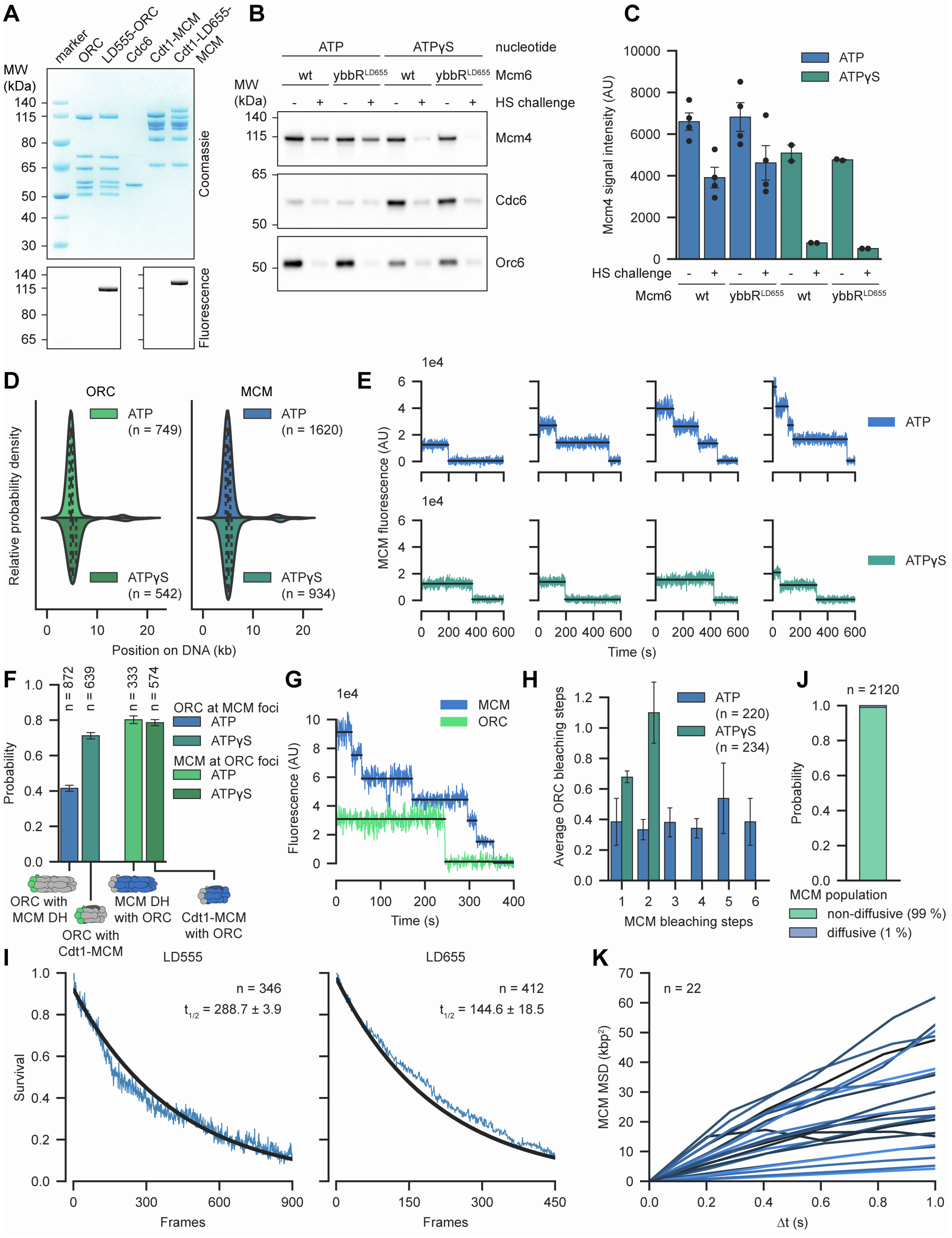

**Figure S1. Additional characterization of MCM loading and diffusion, Related to Figure 1**

(A) SDS-PAGE analysis of purified licensing factors ORC, LD555-ORC, Cdc6, Cdt1-MCM and Cdt1-LD655-MCM by Coomassie Blue staining and fluorescence detection.

(B and C) Ensemble helicase loading assay on bead-bound linear ARS1-DNA with low- or high salt (HS) challenge after licensing in the presence of ATP or ATPγS. (B) Eluted protein was analyzed by Western Blot using protein specific primary antibodies for Orc6, Cdc6 and Mcm4. (C) Integration of Mcm4 signals showed no difference in loading efficiency for Cdt1-MCM containing wildtype or ybbR-LD655 Mcm6 (n = 4 and n = 2 for ATP and ATPγS condition, respectively).

(D) ORC and MCM binding distribution by KDE on ARS1-DNA after helicase loading in the presence of ATP or ATPγS. Lines represent the quartiles of the distribution with the middle line corresponding to the median. Data depicted is the same as shown in Figure 1B.

(E) Example MCM bleaching trajectories after helicase loading in the presence of ATP or ATPγS.

(F) Fraction of MCM foci colocalizing with ORC and ORC foci colocalizing with MCM after helicase loading in the presence of ATP or ATPγS.

(G) Example bleaching trajectory of ATP-loaded, colocalizing MCM and ORC showing six- and one-step bleaching, respectively.
(H) Average number of ORC molecules colocalizing with MCM foci containing one to six (ATP) or one and two (ATPγS) MCM after helicase loading in the presence of ATP or ATPγS.
(I) Lifetimes of single, surface bound LD555 (left) and LD655 (right) dyes. Data from two independent experiments are shown. These were used to determine the number of frames after which half of the dyes bleached which is displayed as mean ± SD as derived from the fit (black lines).
(J) Observed populations of MCM DHs in the presence of 0.5 M NaCl showed 99 % and 1 % in non-diffusive and diffusive DNA binding mode, respectively.
(K) Plot showing the mean squared displacement (MSD) vs Δt of 22 diffusing MCM DHs in the presence of 0.5 M NaCl. Bar plots in (C), (F), and (H) display the mean and SEM.

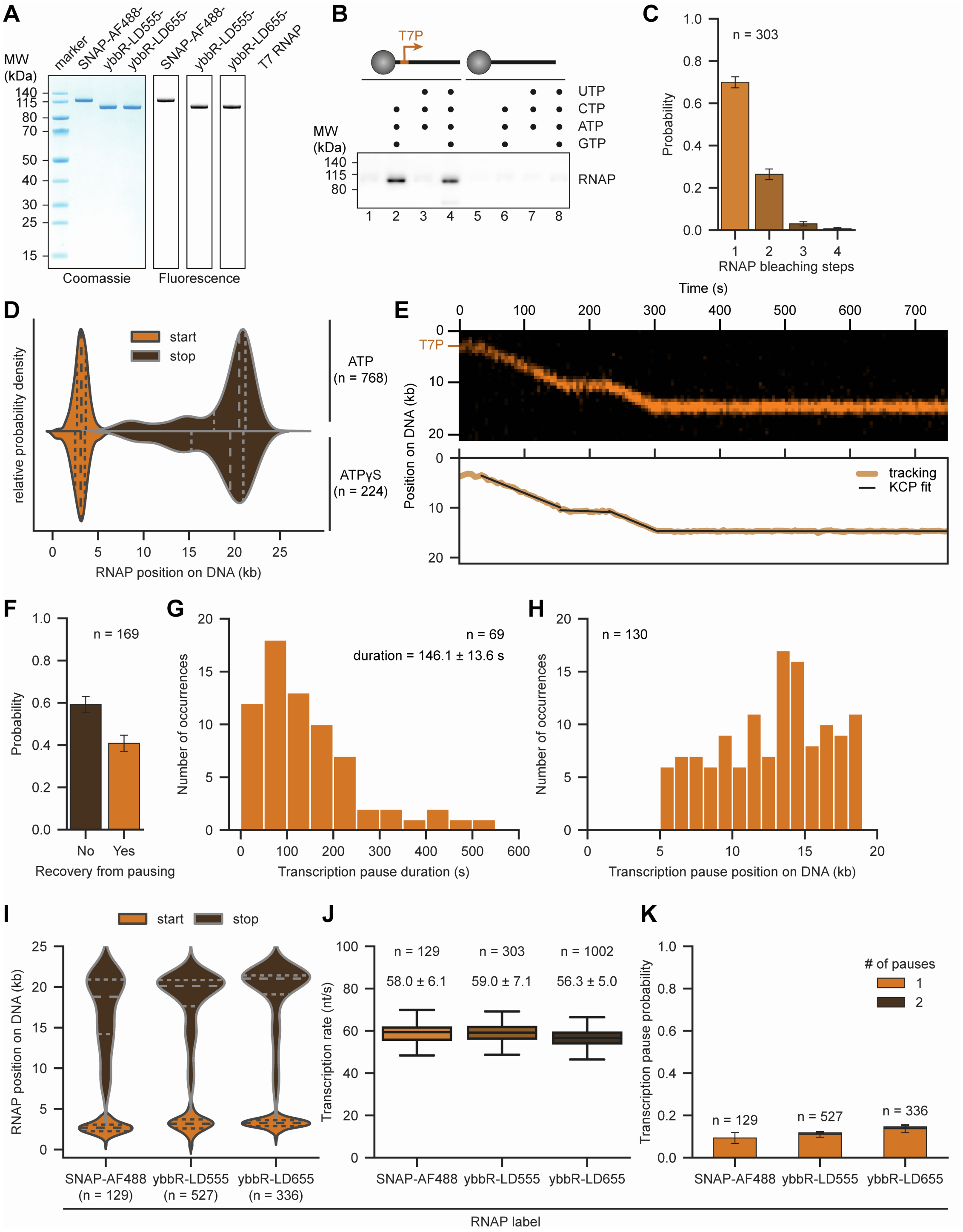


**Figure S2. Additional characterization of the time-coordinated single-molecule transcription assay, Related to Figure 2**

(A) SDS-PAGE analysis of purified SNAP-AF488-, ybbR-LD555- and ybbR-LD655-T7 RNAP by Coomassie Blue staining and fluorescence detection.

(B) Ensemble RNAP loading assay on bead-bound linear DNA with (lanes 1-4) or without (lanes 5-8) an integrated T7P. Formation of stable RNAP elongation complexes were only observed in the presence of an integrated T7P and GTP, ATP and CTP (lanes 2 and 4) but not in the absence of T7P or GTP (lanes 1, 3, 5-8). Eluted RNAP was analyzed by Western Blot using a primary antibody against His tag present in RNAP.

(C) Distribution of RNAP bleaching steps for those loaded at T7P as stalled elongation complexes.

(D) RNAP transcription start and stop sites distribution by KDE on T7P-DNA in the presence of ATP or ATPγS. Lines represent the quartiles of the distribution with the middle line corresponding to the median. Data depicted are the same as shown in Figure 2C.

(E) Representative kymograph (top) with corresponding tracking result (bottom) showing transcribing RNAP with one recovered and one permanent pause detected by a fit with the kinetic change-point (KCP) algorithm (black line).

(F) Fraction of permanent and recovered transcription pauses determined by recovery from pausing within the imaging time.
(G) Distribution of transcription pause durations determined from recovered pauses. Values indicate the mean pause duration ± SEM.
(H) Distribution of transcription pause positions on DNA of permanent and recovered pauses.
(I–K) Transcription start and stop sites (I), rates (J) and pause probability (K) do not differ between SNAP-AF488-, ybbR-LD655- and ybbR-LD655-T7 RNAP. (I) Lines in the violin plot represent the quartiles of the distribution with the middle line corresponding to the median. (J) Values above the box plots indicate the mean ± SD in the presence of ATP derived from a Gaussian fit. Data depicted in (I), (J), and (K) are the same as shown in Figure 2C, 2D, and 2E, respectively. Bar plots in (C), (F), and (K) display the mean and SEM.

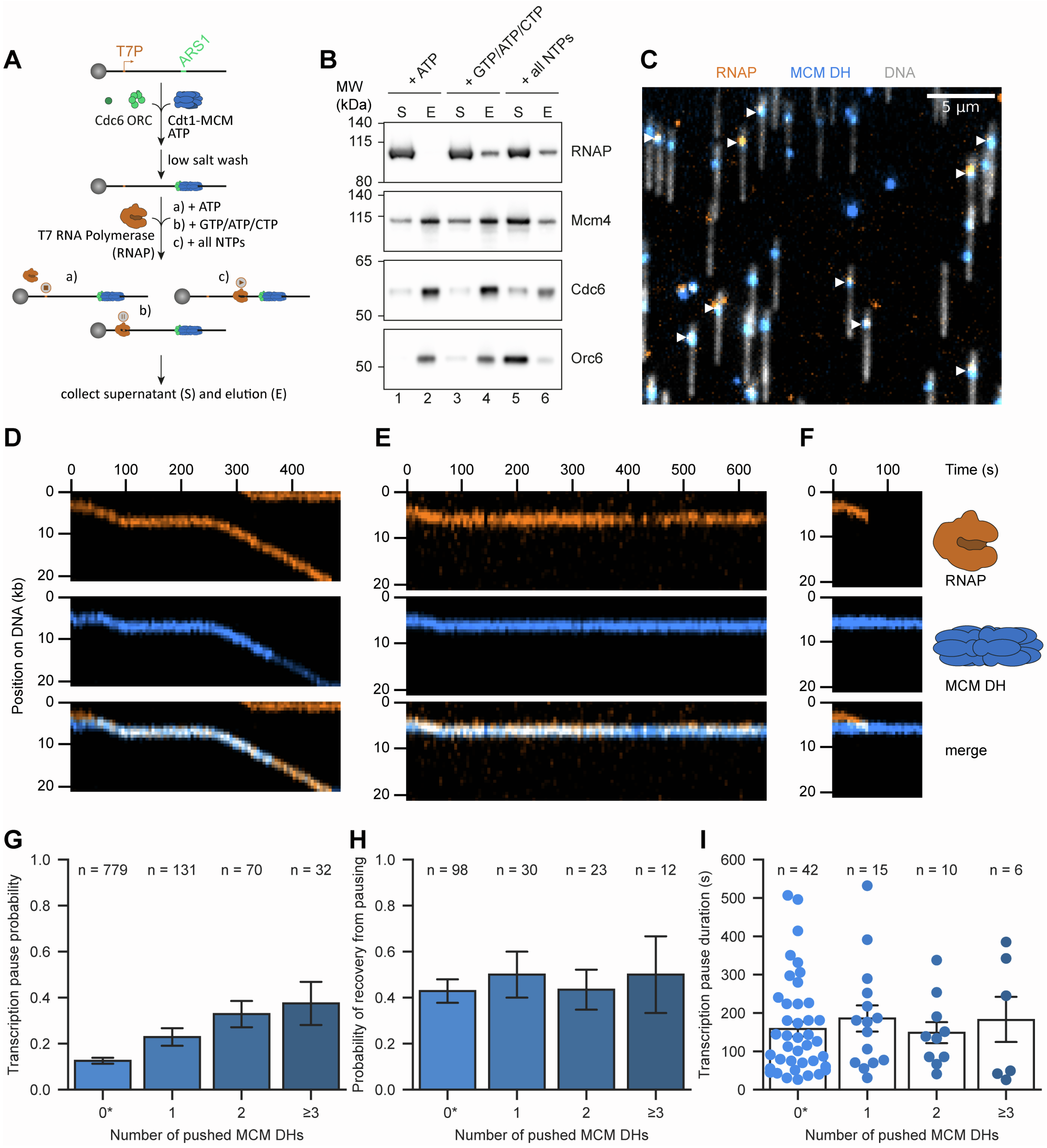


**Figure S3. Additional characterization of transcription-driven MCM DH repositioning, Related to Figure 3**

(A) Schematic of the ensemble RNAP-MCM DH collision assay. MCM DHs were loaded on bead-bound T7P-ARS1-DNA and unbound protein was removed. The reaction was split and incubated with RNAP in the presence of ATP, GTP/ATP/CTP or all NTPs. Finally, the supernatant (S) and elution (E) were collected, corresponding to displaced and DNA-bound protein, respectively.

(B) Western Blot analysis of an ensemble RNAP-MCM DH collision assay as described in (A). In the presence of ATP alone, RNAP could not engage with T7P (lanes 1+2) while in the presence of GTP/ATP/CTP, RNAP formed a stalled elongation complex (lanes 3+4). Both conditions did not show extensive MCM, Cdc6 or ORC displacement from DNA. In the presence of all NTPs, allowing for active transcription, MCM and ORC were efficiently displaced from linear DNA (lanes 5+6).

(C) Example of a partial field of view, showing simultaneous loading of RNAP (amber) and MCM DHs (blue) to the same DNA molecule (gray) as marked by white arrows.

(D–F) Representative kymographs showing recovery from transcription pausing (D), transcription stalling (E) or RNAP ejection (F) upon collision between RNAP (amber) and MCM DH (blue).

(G–I) Influence of MCM DHs on transcription pausing. Transcription pause probability (G), probability of recovery from pausing (H) and transcription pause duration (I) in the absence (0) or presence of (1 to ≥5) pushed MCM DHs. Only DNA molecules with one MCM foci located at ARS1 were analyzed. Bar plots display the mean and SEM. *Data displayed for 0 pushed MCM DHs in (G), (H), and (I) were combined with data shown in panels 2E, S2F, and S2G (ATP condition only), respectively.

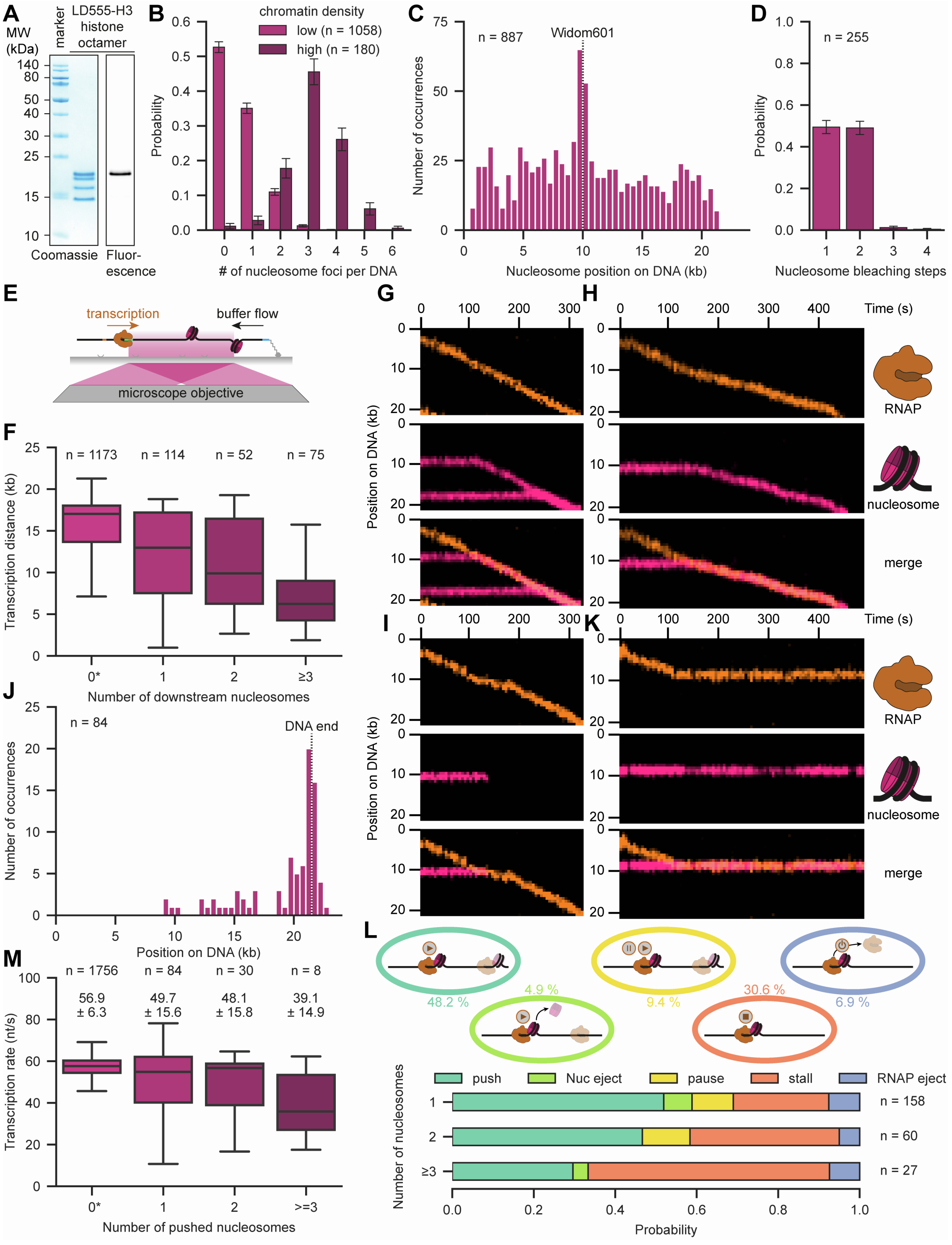


**Figure S4. RNA polymerase can transcribe through individual nucleosomes, Related to Figure 4**

(A) SDS-PAGE analysis of purified LD555-H3 histone octamers by Coomassie Blue staining and fluorescence detection.

(B) Number of nucleosome foci per DNA in low- high density chromatin.

(C) Nucleosome binding distribution histogram on T7P-ARS1-Widom601-DNA for low-density chromatin.

(D) Distribution of nucleosome bleaching steps in low-density chromatin.

(E) Schematic of the single-molecule transcription assay in the presence of nucleosomes. Time-coordinated transcription was set up as described in Figure 2A but on chromatinized DNA.

(F) Boxplot of the global transcription distance in the absence (0) or presence of (1 to ≥3) nucleosomes downstream of T7P. MCMs were not present on molecules analyzed.

(G–I) Representative kymographs demonstrating that RNAP (amber) could transcribe through one or multiple nucleosomes (pink) by nucleosome pushing (G and H) or ejection (I).

(J) Distribution of DNA positions of pushed nucleosomes after transcription stop. Only DNA molecules with one nucleosome foci were considered.

(K) Representative kymograph displaying transcription stalling upon collision between RNAP (amber) and nucleosome (pink).

(L) Quantification of the outcomes of RNAP collisions with a total of 1, 2 or ≥3 nucleosomes. Displayed percentages represent the combined probability irrespective of the number of nucleosomes.

(M) Boxplot of transcription rates in the absence (0) or presence of (1 to ≥3) pushed nucleosomes. Values above the box plots indicate the mean ± SD derived from a Gaussian fit. Bar plots in (B) and (D) display the mean and SEM. *Data displayed for 0 downstream and pushed nucleosomes in (F) and (M) were combined with data shown in Figure 2C and 2D, respectively.

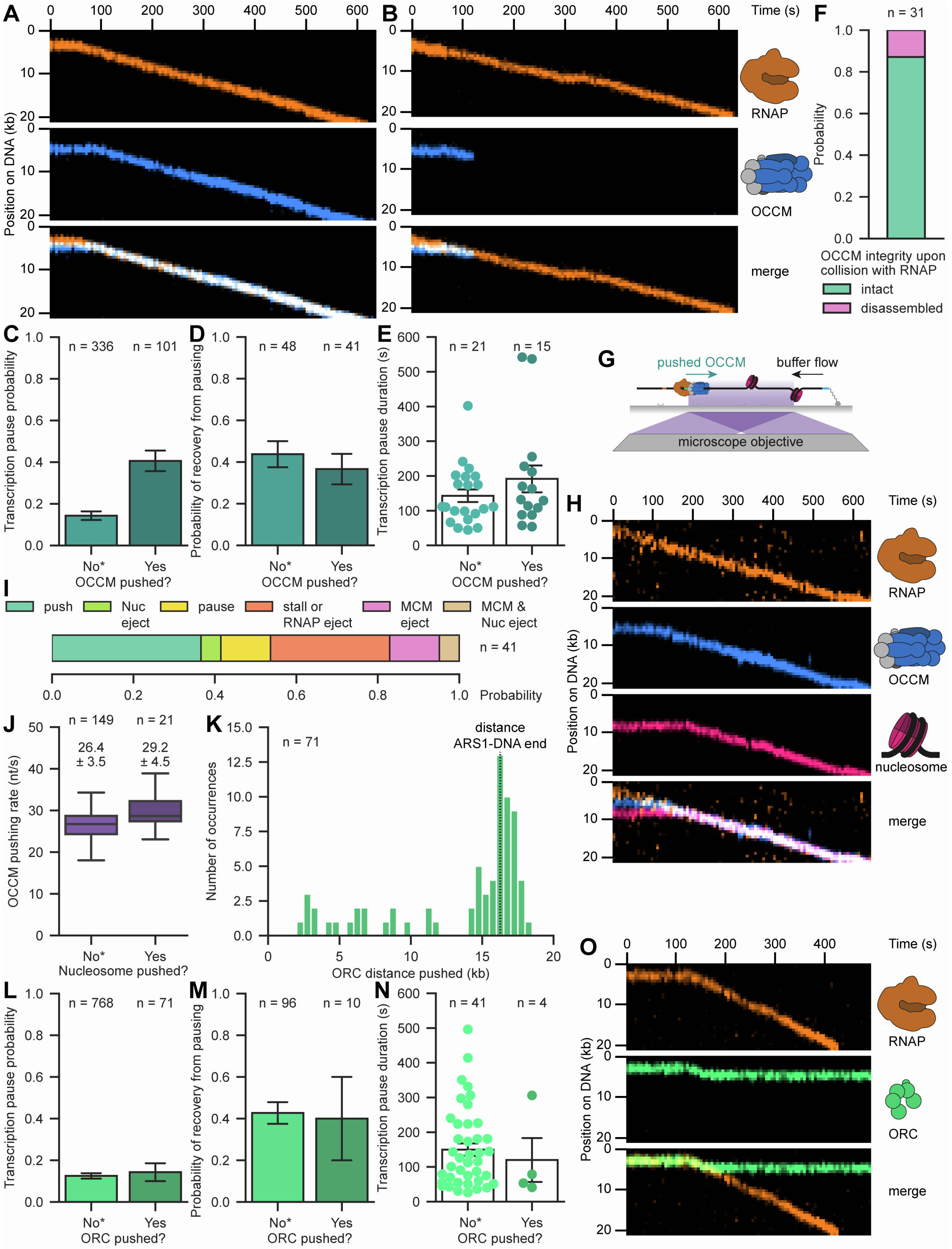

**Figure S5. Additional characterization of transcription-driven OCCM and ORC displacement, Related to Figure 5**

(A and B) Representative kymographs showing that RNAP (amber) could push OCCM (blue) upon collision (A) but OCCM was ejected in some cases (B).

(C–E) Influence of OCCM on transcription pausing. Transcription pause probability (C), probability of recovery from pausing (D) and transcription pause duration (E) in the absence or presence of OCCM. *Data displayed for non-pushed OCCM in (C), (D), and (E) were combined with data shown in Figure 2E, S2F, and S2G (ATPγS condition only), respectively.

(F) OCCM integrity upon collision with RNAP. Probability of OCCM staying intact or being disassembled as judged by the presence of ORC and Cdt1-MCM.

(G) Schematic of the single-molecule OCCM displacement assay in the presence of nucleosomes. OCCM was loaded and pushed by RNAP as described in Figure 5A but on chromatinized DNA.

(H) Representative kymograph demonstrating that RNAP (amber) could displace OCCM (blue) through nucleosomes (pink) by nucleosome pushing.
(I) Quantification of the outcomes of pushed OCCM collisions with nucleosomes.
(J) Boxplot of OCCM pushing rates in the absence or presence of nucleosomes. Values above the box plots indicate the mean ± SD derived from a Gaussian fit. *Data displayed for non-pushed nucleosomes were combined with data shown in Figure 5D.
(K) Distribution histogram of ORC distance pushed by RNAP.
(L–N) Influence of ORC on transcription pausing. Transcription pause probability (L), probability of recovery from pausing (M) and transcription pause duration (N) in the absence or presence of ORC. *Data displayed for no pushed ORC in (L), (M), and (N) were combined with data shown in Figure 2E, S2F, and S2G (ATP condition only), respectively.
(O) Representative kymograph displaying that ORC (green) which colocalized with RNAP (amber) could initially be pushed to ARS1 where it then was bypassed by RNAP. Bar plots in (C), (D), (E), (L), (M), and (N) display the mean and SEM.

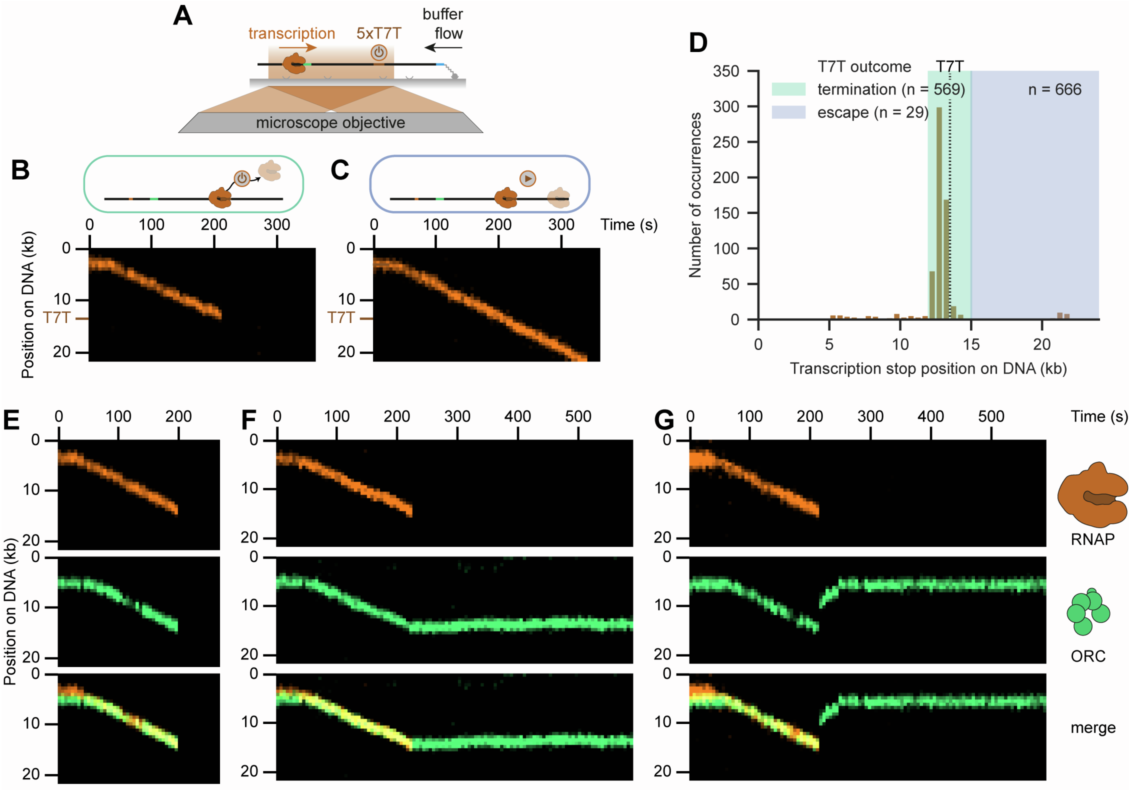


**Figure S6. ORC does not stably associate at random DNA sequences, Related to Figure 6**

(A) Schematic of the single-molecule transcription assay on 5xT7 terminator (T7T)-DNA. Time-coordinated transcription was set up as described in Figure 2A.

(B and C) Representative kymographs displaying transcription termination at the introduced 5xT7T site (B) with a small fraction escaping termination (C).

(D) Transcription is efficiently terminated by 5xT7T. RNAP transcription stop site distribution on 5xT7T-DNA.

(E–G) Representative kymographs showing that although the majority of displaced ORC (green) dissociated (E), ORC could remain stable at the 5xT7T site (F) or slide back to ARS1 after a 1D diffusion in search of the origin (G) upon RNAP (amber) transcription termination.

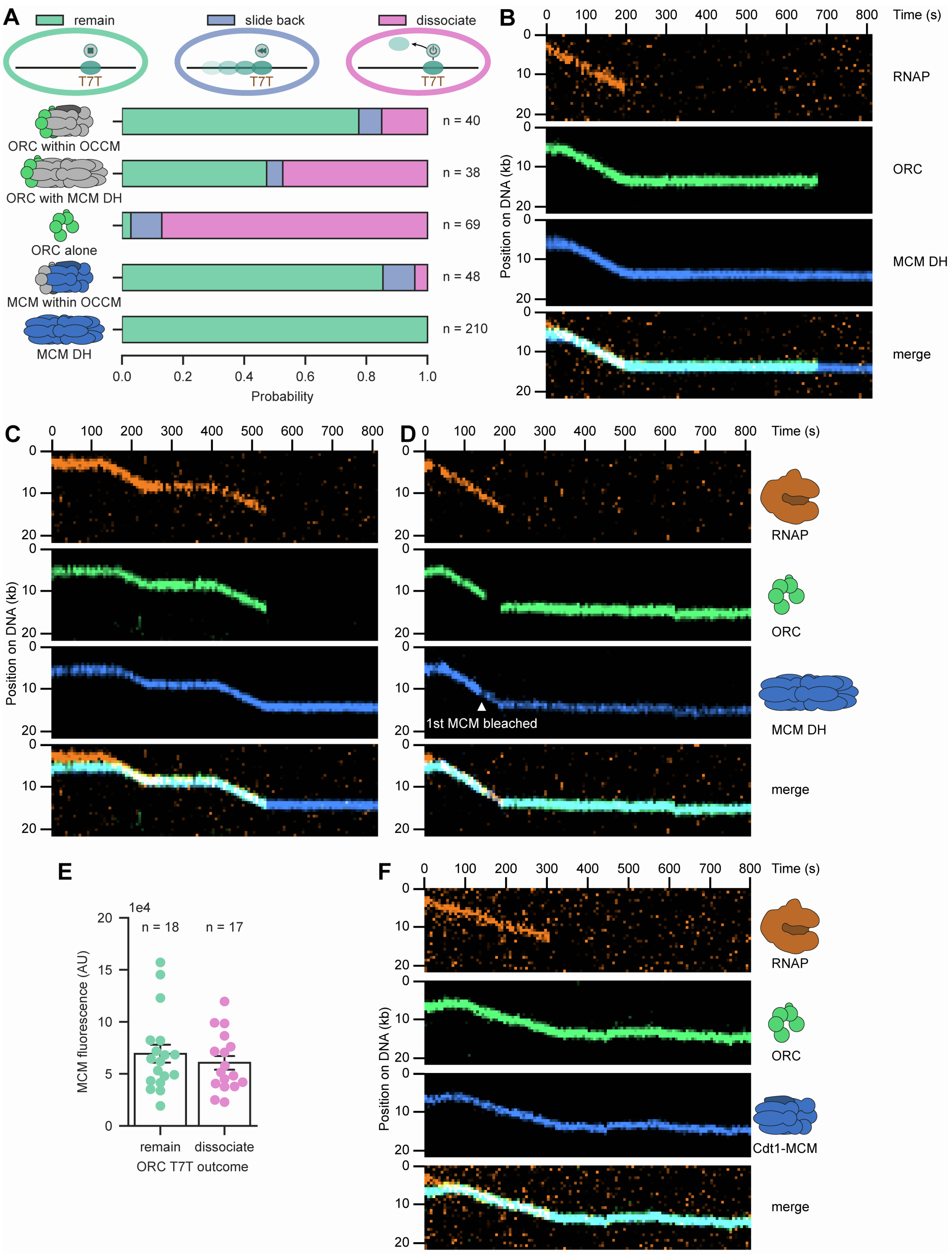


**Figure S7. MCMs stabilize ORC at new locations, Related to Figure 6**

(A) Quantification of the stability of displaced origin licensing factors at the 5xT7T site upon transcription termination.

(B–D) Representative kymographs showing that displaced MCM DHs (blue) always remained stable at the 5xT7T site while an associated ORC (green) could either remain stable (B and D) but also dissociate (C) upon RNAP (amber) transcription termination. The MCM signal and bleaching in (D) corresponds to an MCM DH and not an ORC-containing intermediate. MCM DHs were formed using ATP.

(E) Observed MCM fluorescence for MCM DHs associated with ORC molecules which remained or dissociated at the 5xT7T site upon transcription termination.

(F) Representative kymograph showing that displaced OCCM remained stable at the 5xT7T site, as judged by the presence of ORC (green) and Cdt1-MCM (blue), upon RNAP (amber) transcription termination. OCCM complexes were formed using ATPγS throughout the experiment.
